# *Martharaptor greenriverensis*, a New Theropod Dinosaur from the Lower Cretaceous of Utah

**DOI:** 10.1371/journal.pone.0043911

**Published:** 2012-08-29

**Authors:** Phil Senter, James I. Kirkland, Donald D. DeBlieux

**Affiliations:** 1 Department of Biological Sciences, Fayetteville State University, Fayetteville, North Carolina, United States of America; 2 Utah Geological Survey, Salt Lake City, Utah, United States of America; University of Pennsylvania, United States of America

## Abstract

**Background:**

The Yellow Cat Member of the Cedar Mountain Formation (Early Cretaceous, Barremian?) of Utah has yielded a rich dinosaur fauna, including the basal therizinosauroid theropod *Falcarius utahensis* at its base. Recent excavation uncovered a new possible therizinosauroid taxon from a higher stratigraphic level in the Cedar Mountain Formation than *F. utahensis*.

**Methodology/Principal Findings:**

Here we describe a fragmentary skeleton of the new theropod and perform a phylogenetic analysis to determine its phylogenetic position. The skeleton includes fragments of vertebrae, a scapula, forelimb and hindlimb bones, and an ischium. It also includes several well-preserved manual unguals. Manual and pedal morphology show that the specimen is distinct from other theropods from the Cedar Mountain Formation and from previously described therizinosauroids. It is here named as the holotype of a new genus and species, *Martharaptor greenriverensis*. Phylogenetic analysis places *M. greenriverensis* within Therizinosauroidea as the sister taxon to *Alxasaurus* + Therizinosauridae, although support for this placement is weak.

**Conclusions/Significance:**

The new specimen adds to the known dinosaurian fauna of the Yellow Cat Member of the Cedar Mountain Formation. If the phylogenetic placement is correct, it also adds to the known diversity of Therizinosauroidea.

## Introduction

The Cedar Mountain Formation (Lower Cretaceous) of Utah, USA, preserves a rich theropod paleofauna. The paleofauna of the Ruby Ranch Member includes a large carnosaur similar to *Acrocanthosaurus* [1]. The paleofauna of the lower sequence of the Yellow Cat Member includes the basal therizinosauroid *Falcarius utahensis* [2]–[4], the troodontid *Geminiraptor suarezarum* [5], an unnamed velociraptorine dromaeosaurid [6], and the dromaeosaurine dromaeosaurid *Yurgovuchia doellingi* [6]. The paleofauna of the upper sequence of the Yellow Cat Member includes the coelurosaur *Nedcolbertia justinhofmanni* [7], the dromaeosaurine dromaeosaurid *Utahraptor ostrommaysorum* [8], and an unnamed eudromaeosaur [6].

Here we describe a new theropod specimen from the upper Yellow Cat Member.

The specimen is from the Hayden-Corbett Quarry, Utah state Loc. # Gr.287v, approximately eight miles southeast of the city of Green River, Utah. The specimen is a possible therizinosauroid.

The clade Therizinosauroidea is part of the clade Coelurosauria within the dinosaurian clade Theropoda. Therizinosauroids are known from the Lower and Upper Cretaceous of Asia and North America [9]–[23]. Therizinosauroids range in size from the approximately collie-sized *Beipiaosaurus inexpectus* [9] to the gigantic *Therizinosaurus cheloniformis*, which towered over contemporaneous tyrannosaurids [24]. They exhibit dental specializations for herbivory [2], [9], as do several other coelurosaurian groups [23], [25].

One possible therizinosauroid, *Eshanosaurus deguchiianus*, has been reported from the Lower Jurassic of China [26]. However, the specimen consists only of a single dentary bone, and of its 11 putatively therizinosauroid character states that were reexamined in a recent study [27], all but three (high tooth count, tooth roots wider than crowns, morphology of lateral dentary ridge) are also known in Sauropodomorpha [27]. If the specimen is therizinosauroid, it establishes a ghost lineage ∼65 million years long for Therizinosauroidea [27] and ∼35 million years long for Coelurosauria. We therefore urge caution in the interpretation of *E. deguchiianus* as a therizinosauroid until more of its anatomy is discovered.

## Methods

### Phylogenetic Analysis

We entered data from the new specimen into an updated version (Appendix S1, S2) of a phylogenetic data matrix of Coelurosauria from a recent study [6]. Updates consist of corrections to character 212 (wide distal expansion of scapula) for several therizinosauroid OTUs. Phylogenetic analysis was performed with PAUP 4.0 for Windows [28]. A heuristic search with 1000 random addition-sequence replicates was performed, with no limit to “maxtrees.”

### Nomenclatural Acts

The electronic version of this document does not represent a published work according to the International Code of Zoological Nomenclature (ICZN), and hence the nomenclatural acts contained in the electronic version are not available under that Code from the electronic edition. Therefore, a separate edition of this document was produced by a method that assures numerous identical and durable copies, and those copies were simultaneously obtainable (from the publication date noted on the first page of this article) for the purpose of providing a public and permanent scientific record, in accordance with Article 8.1 of the Code. The separate print-only edition is available on request from PLoS by sending a request to PLoS ONE, 1160 Battery Street, Suite 100, San Francisco, CA 94111, USA along with a check for $10 (to cover printing and postage) payable to “Public Library of Science”.

In addition, this published work and the nomenclatural acts it contains have been registered in ZooBank, the proposed online registration system for the ICZN. The ZooBank LSIDs (Life Science Identifiers) can be resolved and the associated information viewed through any standard web browser by appending the LSID to the prefix “http://zoobank.org/”. The LSID for this publication is: urn:lsid:zoobank.org:pub:12E26311-C182-40D0-8F6F-7FE4B343B261.

**Figure 1:**
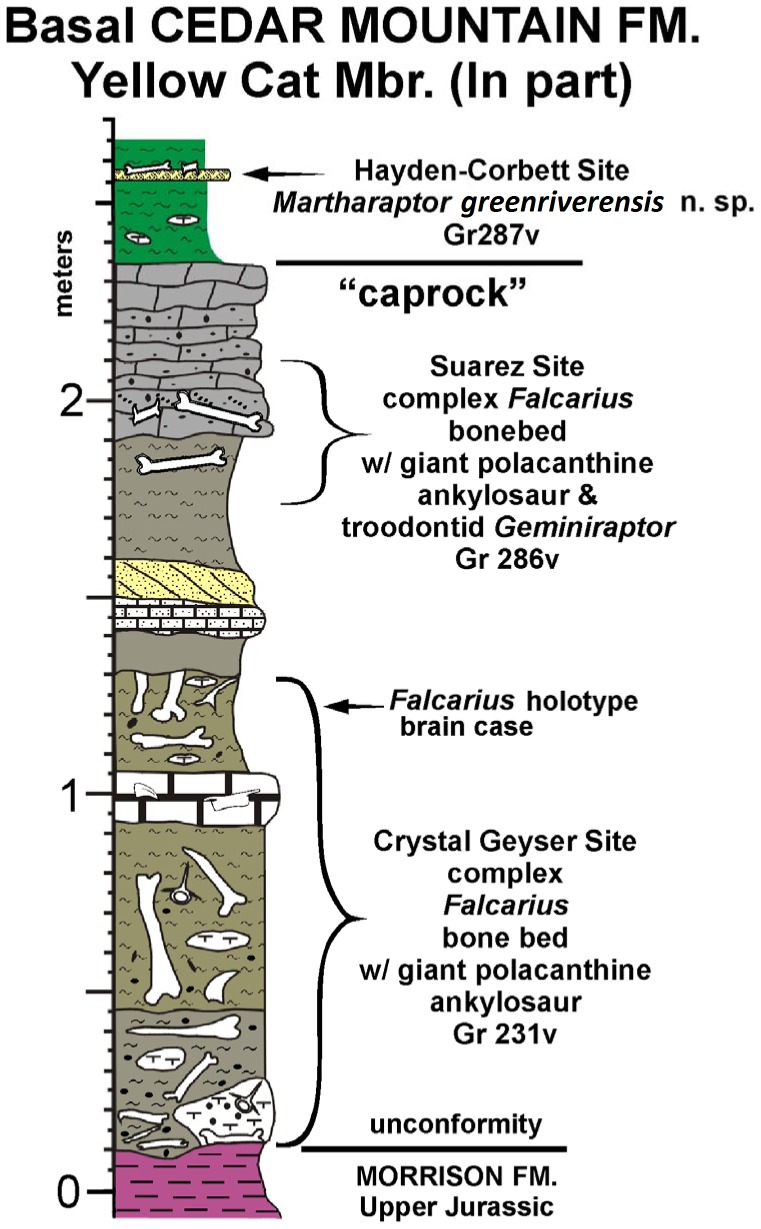
Stratigraphy of the Hayden-Corbett Site (Gr287v). Stratigraphic section of the basal Cedar Mountain Formation of eastern Utah, showing that *Martharaptor* is from a bed stratigraphically higher than those yielding *Falcarius*.

## Results

### Systematic Paleontology

Systematic hierarchy.

Dinosauria Owen, 1841 [29]

Saurischia Seeley, 1887 [30]

Theropoda Marsh, 1881 [31]

Coelurosauria von Huene, 1914 [32]

Therizinosauroidea Maleev, 1954 [33]

*Martharaptor* gen nov.

urn:lsid:zoobank.org:act:9F589210-60D8-4971-95A5-6ACE4D0BA361

*Martharaptor greenriverensis* sp. nov.

urn:lsid:zoobank.org:act:5065191E-53CD-4B48-8C95-8DC7E405F9CB

#### Holotype

The holotype specimen is UMNH VP 21400 (Natural History Museum of Utah, Salt Lake City, Utah).

#### Etymology

The species name refers to the city of Green River in Emery County, Utah. The genus name honors Martha Hayden, who co-discovered the site and has served as the assistant to three successive state paleontologists of Utah over a period of about 25 years.

**Figure 2:**
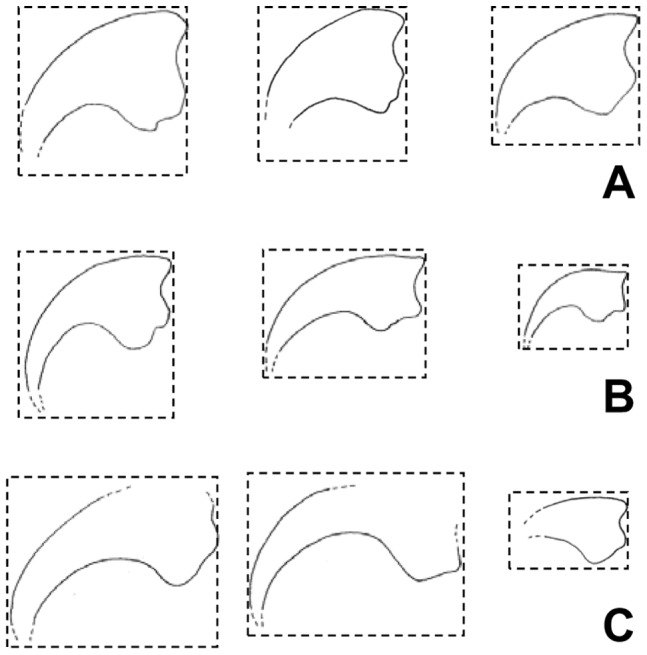
Outlines of manual unguals I (left), II (middle), and III (right) of *Martharaptor greenriverensis* and two other basal therizinosauroids. (A)–*Martharaptor greenriverensis*. (B)–*Falcarius utahensis*, after Zanno [3] (C)–*Beipiaosaurus inexpectus*, drawn from photos by the senior author from a cast of IVPP (Institute of Vertebrate Paleontology and Paleoanthropology, Beijing, China) V 11559. Outlines are to scale within sub-figure A and within sub-figure C but not within sub-figure B or between sub-figures. Note that *M. greenriverensis* differs from *F. utahensis* and *B. inexpectus* in overall ungual shape and flexor tubercle morphology, and differs from *B. inexpectus* in the relative size of the ungual of digit III. The size of the ungual of digit III relative to the others cannot be determined for *F. utahensis*, because no known specimen preserves all three unguals.

#### Geological setting

The Hayden-Corbett Site (Gr287v) is at the top of an approximately 20 cm thick sandstone, interpreted to represent a crevasse splay deposit, in the Yellow Cat Member of the Cedar Mountain Formation approximately 8 miles southeast of Green River, Utah in the immediate vicinity of the Suarez and Crystal Geyser sites, both of which are bone beds yielding abundant specimens of the basal therizinosauroid *Falcarius.* A gravelly, calcareous sandstone often hosting a stromatolitic layer at its top that divides the Yellow Cat Member into an upper and lower sequence [34], [35] has been informally designated the caprock in this area. The Hayden-Corbett site is above this regional marker bed, whereas both the Suarez and Crystal Geyser Quarries are below the caprock (Fig. 1). The upper Yellow Cat Member has been dated at about 124 Ma (early Aptian) based on detrital zircons [36].

**Table 1:** Measurements of bones of the holotype of *Martharaptor greenriverensis*.

Bone	Figure	Dimension	Measurement
Cervical neural arch	3A	Total length	72.70
		Prezygapophysis: transverse width	13.76
Cranial dorsal vertebra	3B–E	Centrum: length	62.07
		Centrum: transverse width	20.5
		Centrum: height of cranial face	41.47*
		Centrum: height of caudal face	45.77
Distal caudal centrum	3F, G	Length	32.41*
		Height	17.6*
Presumed ulna	3H	Length	159*
		Diameter	40.36
Radius	3I	Length	118.11*
		Shaft: diameter at proximalend, in plane of flattened distaltip	22.39
		Shaft: diameter at proximalend, perpendicular to plane offlattened distal tip	22.98
		Distal tip: greatest breadth	56.88
		Distal tip: width perpendicular to greatest breadth	10.74
Scapula	3J, K	Greatest length of proximal fragment	150.03*
		Shaft: width	32.01
		Length of distal fragment	186.3*
Ischium	3L	Length	98.65*
Possible distal pubis	3M	Greatest length	104.18*
		Width perpendicular to greatest length	5019*
Presumed metacarpal I	4A	Length	19.15*
		Distal transverse width	23.3
Presumed manual phalanx I-1	4B	Length	22.40*
		Proximal height	18.95
		Proximal transverse width	19.12
Penultimate manual phalanx	4C	Length	25.4*
		Distal height	20.1
		Distal transverse width	18.23
Penultimate manual phalanx	4D	Length	25.48*
		Distal height	16.23*
		Distal transverse width	15.95
Manual phalanx	4E	Length	18.77*
		Distal height	18.22
		Distal transverse width	19.06
Manual phalanx	4F	Length	22.09*
		Distal height	14.38*
		Distal transverse width	14.96*
Manual ungual (digit I)	4G	Height of articular facet	43.07
		Height of proximal end including flexor tubercle	43.07
		Length perpendicular to articular facet	55.09*
		Greatest transverse width	15.93
Manual ungual (digit I)	4H	Length approximately perpendicular to articular facet	33.25*
		Height approximately parallel to articular facet	28.58*
Manual ungual (digit II)	4I	Height of articular facet	28.76
		Height of proximal end including flexor tubercle	37.93
		Length perpendicular to articular facet	44.44*
		Greatest transverse width	16.18
Manual ungual (digit II)	4J	Height of articular facet	29.09
		Height of proximal end including flexor tubercle	36.53
		Length of proximal fragment perpendicular to articular facet	40.04*
		Greatest transverse width	15.90
		Greatest length of distal fragment	18.25*
Manual ungual (digit III)	4K	Height of articular facet	24.55
		Height of proximal end including flexor tubercle	36.21
		Length perpendicular to articular facet	49.95*
		Greatest transverse width	14.03
Manual ungual (digit III)	4L	Height of articular facet	24.92
		Height of proximal end including flexor tubercle	35.65
		Length perpendicular to articular facet	51.05*
		Greatest transverse width	9.56
Metatarsal I	5A	Greatest length	46.41*
		Distal width	19.09
		Distal depth	15.03
Metatarsal II	5B	Length	55.42*
		Proximal transverse width	31.22
		Proximal depth	30.89
Metatarsal II	5D	Length	37.76*
		Distal transverse width	30.72
		Distal depth	38.73
Presumed metatarsal III	5C	Length	23.22*
		Proximal transverse width	25.22*
		Proximal depth	29.98
Metatarsal IV	5E	Length	50.48*
		Distal transverse width	29.24*
		Distal depth	28.07*
Pedal phalanx	6A	Length	20.62*
		Proximal transverse width	27.84
		Proximal depth	24.52
Pedal phalanx	6B	Length	31.14*
		Proximal transverse width	29.03
		Proximal depth	23.32
Pedal phalanx	6C	Length	27.20.41*
		Proximal transverse width	25.17*
		Proximal depth	27.51
Pedal phalanx	6D	Length	41.98*
		Proximal transverse width	23.98*
		Proximal depth	30.89
Pedal phalanx	6E	Length	32.21*
		Proximal transverse width	26.15
		Proximal depth	32.88
Pedal phalanx	6F	Length	46.17*
		Proximal transverse width	25.43*
		Proximal depth	19.71*
Pedal phalanx	6G	Length	30.54*
		Distal transverse width	25.81
		Distal depth	20.64
Pedal phalanx	6H	Length	25.15*
		Distal transverse width	27.37
		Distal depth	18.71*
Pedal phalanx	6I	Length	25.86*
		Proximal transverse width	28.59
		Proximal depth	25.09
Pedal phalanx	6J	Length	16.87*
		Proximal transverse width	23.45
		Proximal depth	20.51*
Pedal phalanx	6K	Length	27.67*
		Distal transverse width	25.61*
		Distal depth	20.00*
Pedal ungual (digit I)	6L	Length, approximately perpendicular to articular facet	29.88*
		Height approximately parallel to articular facet	26.95*
Pedal ungual	6M	Height of articular facet	26.75
		Height of proximal end including flexor tubercle	29.01*
		Length perpendicular to articular facet	26.99*
		Greatest transverse width	13.63

Measurements are in mm. For cases in which the total measurement cannot be given because part of the bone is missing, the measurement of the preserved portion is given and marked with an asterisk. For pedal bones, “depth” refers to the dorsoplantar dimension.

The bones of the holotype of *M. greenriverensis* were found disarticulated. However, there is no indication that more than one individual is present in the sample. All the skeletal material, including the surface “float” that drew attention to the skeleton, was found in an area of less than two square meters, and most of it is from an excavated mudstone block that is less than one square meter in lateral area and less than 0.2 m deep. There is no duplication of elements, the sizes of the bones are consistent with their having come from a single individual, and there are no morphological indicators that multiple taxa are present. We are therefore confident that the material can all be assigned to a single individual.

**Figure 3:**
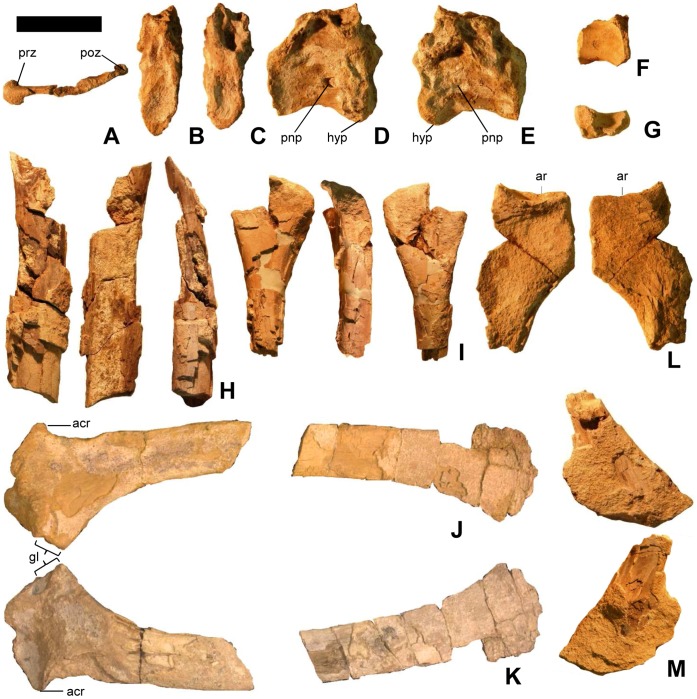
Vertebrae, scapula, forelimb bones, and pelvic bones of *Martharaptor greenriverensis* (UMNH VP 21400). (A)–Partial cervical neural arch, dorsal view. (B–E)–Cranial dorsal centrum in cranial (B), caudal (C), right lateral (D), and left lateral (E) views. (F–G)–Distal caudal centrum in lateral (F) and ventral (G) views. (H)–Possible ulna. (I)–Possible radius. (J–K)–Left scapula in lateral (J) and medial (K) views. (L)–Proximal end of ischium. (M)–Possible distal end of pubis Scale bar = 50 mm. acr  =  acromium process, ar  =  acetabular rim, gl  =  glenoid, hyp  =  hypapophysis, poz  =  postzygapophysis, pnp  =  pneumatopore, prz  =  prezygapophysis.

#### Diagnosis

Theropod dinosaur with the following combination of character states: cervical prezygapophyses not flexed; cranial dorsal vertebrae with hypapophyses and a single pair of pneumatopores; manual unguals without proximodorsal lips and with prominent flexor tubercles and strong curvature; manual unguals in which total length perpendicular to the articular facet is subequal to total height parallel to the articular facet; ungual of manual digit III nearly as large as that of digit II; distal end of scapula expanded; proximal end of ischium laterally compressed; metatarsal I proximally attenuated and distally reduced in transverse width relative to the other metatarsals; all metatarsals distally non-ginglymoid; fourth metatarsal distally attenuated immediately proximal to condyles; pedal unguals laterally compressed and strongly curved; first pedal ungual smaller than the others.

**Figure 4:**
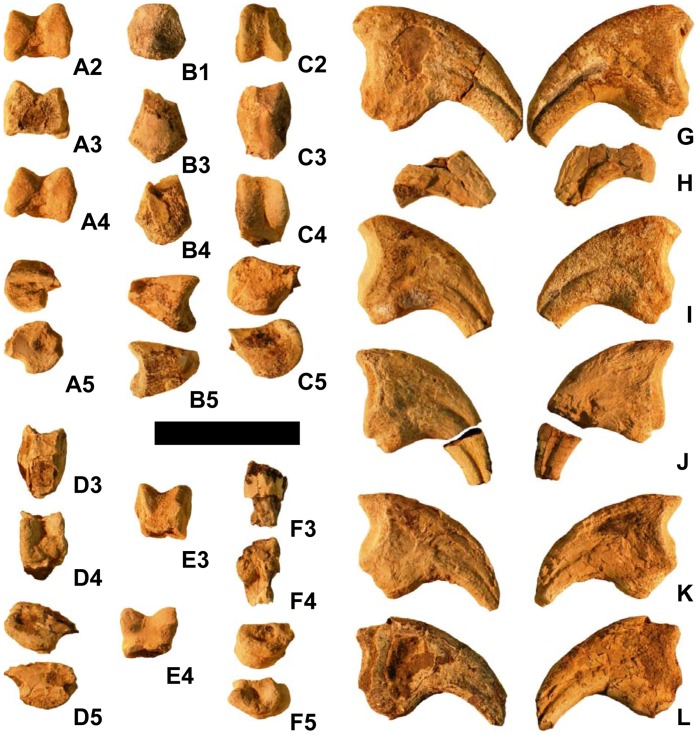
Manual bones of *Martharaptor greenriverensis* (UMNH VP 21400). (A)–Presumed metacarpal I. (B)–Presumed phalanx I-1. (C)–Penultimate phalanx. (D) Penultimate phalanx. (E)–Unidentified phalanx. (F)–Unidentified phalanx. (G)–Ungual of digit I. (H)–Ungual of digit I. (I)–Ungual of digit II. (J)–Ungual of digit II. (K)–Ungual of digit III. (L)–Ungual of digit III. Scale bar = 50 mm. Numbers on sub-figures refer to proximal (1), distal (2), dorsal (3), palmar (4), and side (5) views; for side views, whether the side is medial or lateral cannot be determined.

No other theropod dinosaur exhibits this combination of character states. However, because of the fragmentary nature of the specimen, it is important to be specific about how this combination of character states distinguishes the specimen as a new taxon. Therefore, below we show how these character states distinguish the new specimen from other theropods of the Cedar Mountain Formation and from previously described therizinosauroids.

**Figure 5:**
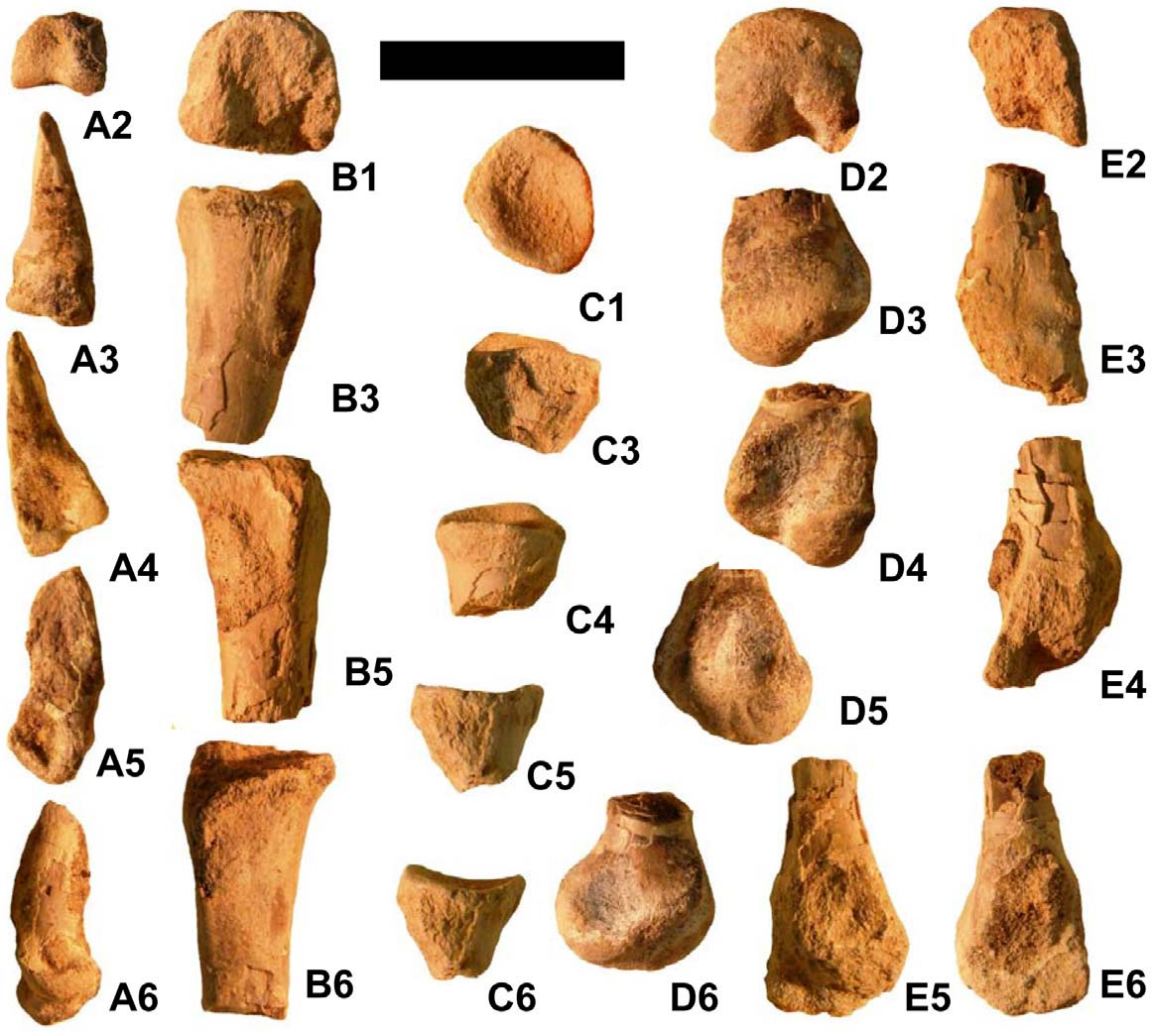
Metatarsals of *Martharaptor greenriverensis* (UMNH VP 21400). (A)–Left metatarsal I. (B)–Left metatarsal II. (C)–Presumed left metatarsal III. (D)–Right metatarsal II. (E)–Right metatarsal IV. Scale bar = 50 mm. Numbers on sub-figures refer to proximal (1), distal (2), dorsal (3), plantar (4), medial (5), and lateral (6) views.

*Martharaptor greenriverensis* can be distinguished from the Cedar Mountain Formation coelurosaur *Nedcolbertia justinhofmanni* by differences in manual and pedal morphology. In *N. justinhofmanni* the manual unguals are nearly straight, and the flexor tubercle of the first manual ungual is strongly pendant and approximately half the height of the articular facet. The proximal surface of metatarsal II is subtriangular, and that of metatarsal III is a craniocaudally elongate rectangle [7]. In contrast, the manual unguals of *M. greenriverensis* are strongly curved, the proximal surface of metatarsal II is nearly square, and the proximal surface of metatarsal III is approximately as wide transversely at it is long craniocaudally.

**Figure 6:**
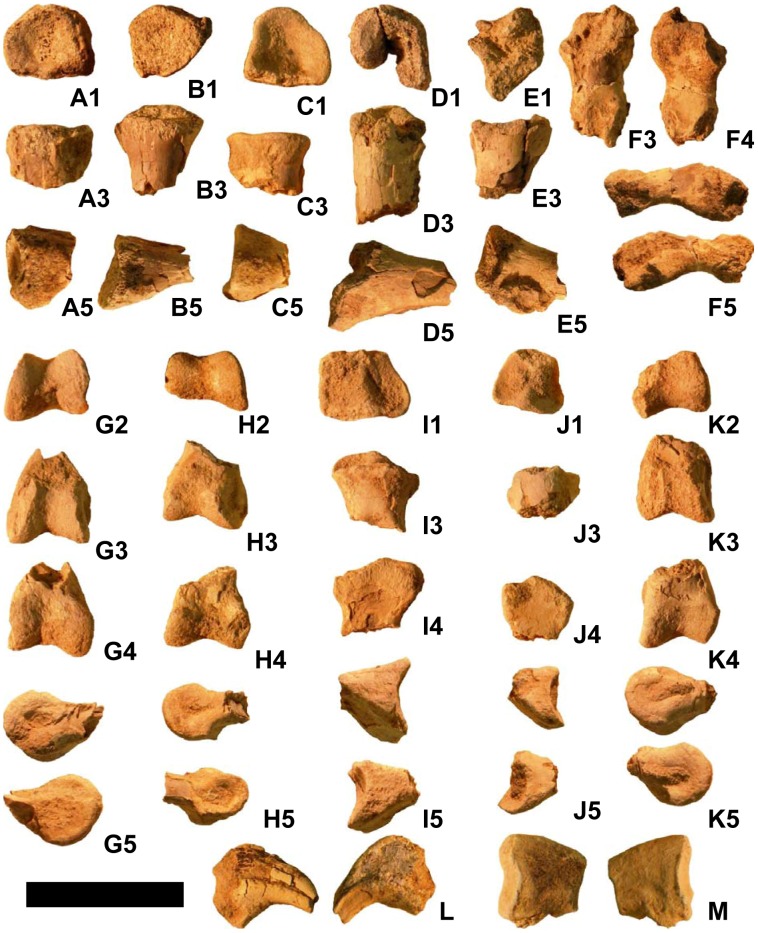
Pedal phalanges of *Martharaptor greenriverensis* (UMNH VP 21400). (A–K)–Unidentified phalanges. (L)–Ungual of digit I. (M)–Ungual of unidentified digit (II, III, or IV). Scale bar = 50 mm. Numbers on sub-figures refer to proximal (1), distal (2), dorsal (3), plantar (4), and side (5) views; for side views, whether the side is medial or lateral cannot be determined. Phalanx H articulates well with phalanx J, and phalanx G articulates well with phalanx I.

In the known material of *M. greenriverensis* there are no preserved bones in common with the troodontid *Geminiraptor suarezarum* [5], the dromaeosaurids *Utahraptor ostrommaysorum* [8] and *Yurgovuchia doellingi* [6], or the unnamed dromaeosaurids from the Cedar Mountain Formation [6]. However, *M. greenriverensis* lacks character states that are present in troodontids and dromaeosaurids, such as flexed cervical prezygapophyses, a distally unexpanded scapula, and strongly pendant flexor tubercles on the manual unguals.

**Figure 7:**
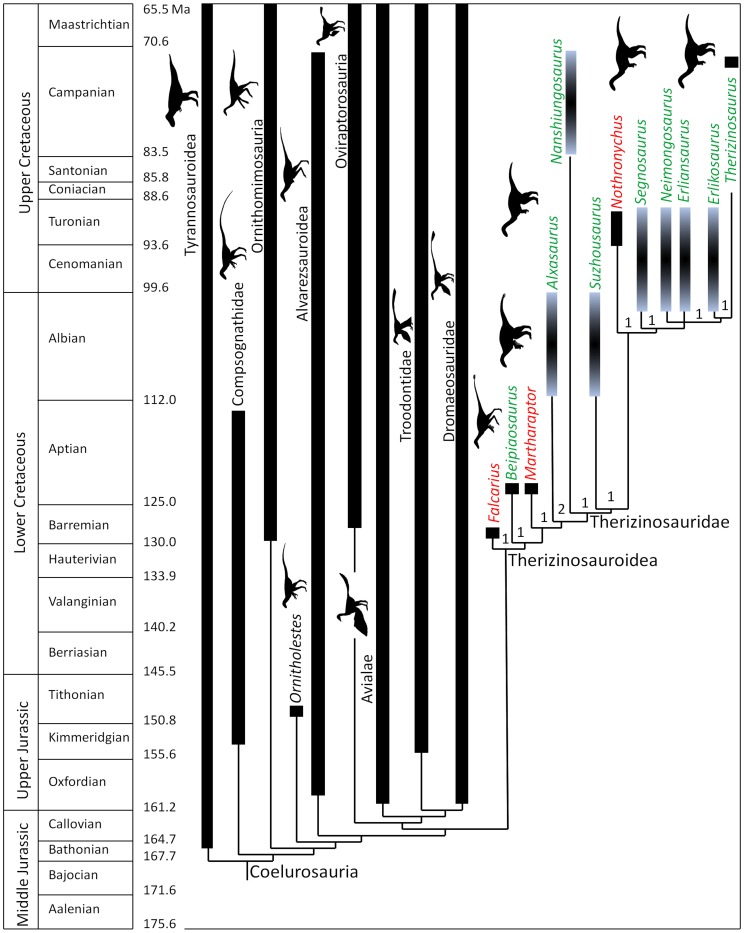
Phylogenetic position of *Martharaptor greenriverensis* within Coelurosauria, as found by this study. Genus names in green are Asian therizinosauroids, and those in red are North American therizinosauroids. Numbers at therizinosauroid clades indicate decay indices (Bremer values).

*M. greenriverensis* can be distinguished from *Falcarius utahensis*, a therizinosauroid from the Cedar Mountain Formation, by the morphology of its manual unguals and fourth metatarsal. In *F. utahensis* the shape of the first manual ungual is markedly different from the others [2], whereas in *M. greenriverensis* all manual unguals resemble each other in shape (Fig. 2). The shaft of the third manual ungual is more gracile in *F. utahensis* [2] and is taller (deeper in the dorso-palmar dimension) in *M. greenriverensis* (Fig. 2). In *M. greenriverensis* metatarsal IV is more strongly beveled distally than it is in *Falcarius* [3].

Other therizinosauroids differ from *M. greenriverensis* in the following ways. In *Beipiaosaurus inexpectus* the manual unguals are more gracile and the third manual is much smaller than the others [9], whereas in *M. greenriverensis* the manual unguals are deep and the third ungual is only slightly smaller than the others (Fig. 2). In *Alxasaurus elesitaiensis* and members of Therizinosauridae other than *Nothronychus* the dorsal surface of each manual ungual arches above the level of the proximal articular facet [12], [19], [21], whereas such is not the case in *M. greenriverensis*. In members of Therizinosauridae the proximal surface of metatarsal I is robust and articulates with the tarsus [16], [20], [23], whereas that of *M. greenriverensis* is attenuated and exhibits a lateral facet for articulation with the medial side of metatarsal II. In members of Therizinosauridae the distal surface of metatarsal IV is perpendicular to the shaft [16], [20], [23], whereas it is beveled in *M. greenriverensis*.

### Specimen Description

Preserved bones of *M. greenriverensis* are described below. Table 1 lists the measurements of these bones.

A partial neural arch from a cervical vertebra preserves the left zygapophyses and the lateral lamina connecting them (Fig. 3A). The prezygapophysis is not flexed, but little else can be discerned on this fragment.

The centrum of one cranial dorsal vertebra is preserved (Fig. 3B–E), and it has been transversely flattened post-mortem. The cranial and caudal articular surfaces of the centrum are shallowly concave. The relatively size of the small hypapophysis at the cranial end of the centrum suggests that this vertebra is the first or last hypapophysis-bearing vertebra in the series. The proportions of the centrum suggest that it is more likely the first than the last such vertebra, upon comparison with *Falcarius* [4] and the oviraptorosaur *Nomingia* [37]. A distinct pneumatopore is present immediately caudodorsal to the hypapophysis.

The ventral part of another centrum with a nearly flat ventral surface is relatively small, suggesting that it is from the tail (Fig. 3F–G).

The distal end of a bone is interpreted as a radius (Fig. 3I). The distal end is incomplete, but the preserved portion is expanded and flattened, as is usual in a derived coelurosaurian radius. The shaft is round in cross-section.

The partial shaft of another long bone is interpreted as an ulna (Fig. 3H). It is round in cross-section and internally hollow and has been split lengthwise down the middle. It appears slightly bowed, but this may be an optical illusion created by its pattern of missing bone chunks. Its diameter is larger than that of the presumed radius, which is consistent with a theropod ulna.

The distal tip of a manual element, interpreted as metacarpal I from the right hand, is preserved (Fig. 4A). It is ginglymoid and asymmetrical, with the medial condyle extending further distally than the lateral condyle.

The proximal tip of a manual element that may be phalanx I-1 is preserved (Fig. 4B). It fits tightly with the presumed right first metacarpal. The distal tips of four other non-ungual manual phalanges are preserved, one much smaller than the other two (Fig. 4C–F). All three are distally ginglymoid and symmetrical. Two are penultimate phalanges, as indicated by the close spacing of the distal condyles; these two phalanges exhibit a tight fit with manual unguals I and II.

Five nearly complete manual ungual phalanges are preserved, each with its tip broken off (Fig. 4G–L). None of the manual unguals of *M. greenriverensis* has the proximodorsal “lip” that is found on the manual unguals of some oviraptorosaurian and paravian coelurosaurs [38]. All of the manual unguals of the specimen are similar in overall size, but one is 13–20% taller proximally than the others (Fig. 4G). It is therefore most likely from the first finger. Two of the other nearly complete unguals have a flexor tubercle that is more bulbous than that of the others. Their articular facets are smaller than those of the other unguals, so they are most likely from the third finger. The other two unguals are therefore likely from the second finger. An isolated piece of an ungual from near the tip appears to be from the second finger. A piece of the palmar curve of another ungual matches the curvature of the largest ungual and is therefore probably from the first finger of the other hand. One of the unguals of digit II is missing a piece from the middle, so that the proximal and distal portions are separated by a gap (Fig. 4J).

The left scapula is preserved, with a piece missing from the middle of its shaft (Fig. 3J–K). Its distal end is moderately expanded, as in the therizinosauroids *Beipiaosaurus* [11] and *Suzhousaurus* [14]. In *Falcarius* and other therizinosauroids, breakage prevents assessment of whether a distal expansion is present [2], [20], [21], [23]. The acromion area juts out prominently, like a short hatchet head. Its shape resembles that of the preserved portion of the acromion of *Falcarius* [4]. As in *Falcarius* [4] and *Beipiaosaurus* [11], there is no extension of the glenoid onto the lateral surface of the scapula.

The proximal end of the ischium is preserved (Fig. 3L). It resembles its counterpart in *Falcarius* [4]. The preserved part of the bone is laterally compressed. Part of the acetabular rim and the cranial edge of the proximal shaft are preserved.

The flattened, expanded end of a bone with a shaft is preserved (Fig. 3). Enough of the shaft is broken off to make it difficult to determine whether the shaft is round or flattened in cross-section. The expanded end is broken into a shape that resembles the deltopectoral crest of a proximal humerus, but this is an artifact. The diameter of the shaft is similar to that of the presumed radius and less than that of the presumed ulna. This is inconsistent with a humerus, the shaft of which is larger in diameter than a radius or ulna. It is possible that this is the distal end of a pubis, in which case the expanded end is a partial pubic boot resembling that of *Falcarius* [4] more than those of other therizinosauroids [15], [23].

The first metatarsal of the left foot is missing its proximal tip (Fig. 5A). As in most other theropods, including the basal therizinosauroids *Falcarius* [4] and *Beipiaosaurus* [9], the proximal end tapers. This differs from the condition in Therizinosauridae, in which the proximal end is robust and articulates with the tarsus [16], [20], [23]. In *M. greenriverensis* the medial surface of metatarsal I exhibits a distinct facet for articulation with the lateral surface of metatarsal II. The condyles are round in medial and lateral view, and the medial condyle extends slightly further distally than the lateral condyle. The articular surface for phalanx I-1 extends onto the dorsal surface of the metatarsal.

The preserved proximal end of the left second metatarsal has a nearly square proximal surface (Fig. 5B). Only the distal end of the right second metatarsal is preserved (fig. 5D). It is a rounded knob, with two distinct condyles only on the plantar surface. Of those, the lateral condyle is much more bulbous than the medial condyle.

The proximal end of another metatarsal (Fig. 5C) exhibits good fit with the proximal end of right metatarsal II, but with so little of the bone preserved it is difficult to be certain that it is the right third metatarsal.

The distal end of the right fourth metatarsal (Fig. 5E) has much of its distal surface eroded, but enough is preserved to tell that the distal end is not ginglymoid and that two distinct condyles are present only on the plantar side. Of these the lateral condyle is more bulbous than the medial condyle. The medial condyle projects much further distally than the lateral condyle, producing a striking asymmetry and a beveling that is much stronger than that of metatarsal II. A small bit of the shaft is preserved, just enough to show that it was constricted distally and that its distal end was not in contact with metatarsal III, both of which character states it shares with *Falcarius* [4] and the oviraptorosaur *Chirostenotes* (CMN [Canadian Museum of Nature, Ottawa, Ontario] FV 8538).

Several partial pedal phalanges are preserved (Fig. 6). Three are proximal ends. In each of the three the articular surface is concave and lacks a vertical ridge, which suggests that they represent phalanx 1 of their respective toes. In each of the three the flattened plantar surface suggests a phalanx rather than a metatarsal. In all three the transverse width is consistent with phalanx II-1 or IV-1, too large to be phalanx I-1, and too small to be phalanx III-1, given the size of metatarsal I and distal metatarsal II. The distal ends of three ginglymoid pedal phalanges show slight asymmetry between the condyles. For two of the three, the corresponding proximal end of another phalanx is present that exhibits good fit (see caption to Fig. 6). The proximal ends of two more pedal phalanges are present.

Only one pedal phalanx preserves its whole length (Fig. 6F). Its dorsal surface and the proximal and distal ends are eroded. Its small size in comparison with the other pedal elements is consistent with its having come from toe IV.

Two pedal unguals are present (Fig. 6L–M); both are laterally compressed and strongly curved, as in other therizinosauroids [12], [18], [22] and unlike other non-paravian theropods. Only the proximal extremity of one is preserved. The other is considerably smaller and exhibits a size consistent with that of the ungual of the first toe. Its tip and its proximal extremity are missing. Its curvature is stronger than that of any of the manual unguals.

In addition to the material described above, the sample includes many bone shards and tiny fragments that are too incomplete to identify.

### Phylogenetic Analysis

The phylogenetic analysis found 1444 trees of 1303 steps. For these trees the consistency index is 0.3761, the homoplasy index is 0.6239, the retention index is 0.8136, and the rescaled consistency index is 0.3059. The strict consensus tree places *M. greenriverensis* within Therizinosauroidea as the sister taxon to *Alxasaurus* + Therizinosauridae (Fig. 7). Outside Therizinosauroidea the topology of the tree is identical to that found by the study that used the previous draft of the present data matrix [6] and nearly identical to those found by several other recent phylogenetic studies of Coelurosauria [23], [39]–[41]. A decay analysis found low decay indices (Bremer values) of only 1 or 2 for therizinosauroid clades (Fig. 7). This is likely due to the large amount of missing data, a phenomenon that lowers decay indices, in *Martharaptor*. Decay indices outside Therizinosauroidea are identical to those from the previous study [6].

Synapomorphies of Therizinosauroidea or its sub-clades that are demonstrably present on *Martharaptor* include: distal scapular expansion; ungual of finger III approximately the same size as that of finger II; and strongly curved hallucal ungual. A full list of synapomorphies of the therizinosauroid clades, as found by this analysis, is given in Appendix S3.

## Discussion

*Martharaptor greenriverensis* adds to the known dinosaurian fauna of the Cedar Mountain Formation. If it is truly therizinosauroid, it also adds to the known diversity of Therizinosauroidea. A greater amount of known skeletal material would yield greater confidence in the phylogenetic placement of *Martharaptor*, and we acknowledge the possibility that a future phylogenetic study with more skeletal material could recover a different phylogenetic placement for the taxon. However, the known material exhibits no character states that are inconsistent with basal therizinosauroid status. Also, the strong curvature and lateral compression of the pedal unguals is unknown in non-therizinosauroid theropods outside Paraves, and the material lacks typical paravian traits such as flexed cervical prezygapophyses, strongly pendant flexor tubercles on all manual unguals, and ginglymoid metatarsals.

## Supporting Information

Appendix S1 **Character List for Phylogenetic Analysis of Coelurosauria.**
(DOC)

Appendix S2 **Phylogenetic Data Matrix for Phylogenetic Analysis of Coelurosauria.**
(DOC)

Appendix S3 Synapomorphies of clades within Therizinosauroidea.
(DOC)
