# Supplementary material for: Martharaptor greenriverensis, a New Theropod Dinosaur from the Lower Cretaceous of Utah
Source: PLoS One. 2012 Aug 29;7(8):e43911. doi: 10.1371/journal.pone.0043911 (PMC3430620; doi:10.1371/journal.pone.0043911)
Supplement: Appendix S2 — Phylogenetic Data Matrix for Phylogenetic Analysis of Coelurosauria. (DOC) [file pone.0043911.s002.doc]

**Appendix S2: Phylogenetic Data Matrix for Phylogenetic Analysis of Coelurosauria**

Dilophosaurus 0 0 0 0 0 0 0 0 0 0 0 0 1 0 0 0 0 0 ? 0 0 0 0 0 0 0 0 ? ? 0 0 0 0 0 0 ? 0 1 1 0 0 2 ? 0 0 1 0 0 1 0 0 0 0 0 0 0 0 0 ? 0 0 00 0 0 ? 0 ? ? ? ? ? ? ? 0 0 0 0 0 0 0 0 0 0 0 0 0 0 1 0 0 0 0 0 0 0 0 ? ? ? ? ? ? ? ? 0 0 0 0 0 0 0 0 0 0 0 0 0 1 0 0 0 0 0 0 0 0 0 0 0 0 0 0 0 0 0 0 0 0 0 0 0 0 0 0 0 0 0 0 1 0 0 1 0 0 0 0 1 3 0 0 0 0 0 0 0 0 0 0 1 1 0 0 1 0 0 1 0 0 0 0 0 0 0 0 0 0 0 0 0 0 ? 0 0 0 ? 0 0 0 0 0 0 0 ? 0 ? ? ? ? ? ? 2 0 0 0 0 0 0 0 0 0 0 0 0 0 0 0 0 0 0 0 1 0 0 0 0 0 1 0 0 ? 1 0 1 0 0 0 0 0 0 0 0 0 0 0 0 0 0 0 1 0 0 0 0 0 0 0 0 1 0 0 0 0 0 0 0 0 1 0 0 0 0 0 0 0 0 0 0 0 0 0 0 0 0 0 0 0 0 0 0 0 0 0 0 0 0 0 0 0 0 0 0 0 0 2 0 0 0 0 0 0 0 0 0 0 0 0 0 0 0 0 0 0 0 0 0 0 0 0 1 0 0 0 0 0 0 0 0 0 0 1 0 0 0 0 1 0 0 0 0 0 0 0 0 0 0 0 0 0 0 0 0 0 0 0 0 0 0 0 0 0 0 0 0 0 0 0 0 ? 0 0 0 0 0

Allosaurus 0 0 0 0 0 0 0 0 0 0 0 0 1 0 0 0 0 1 ? 0 0 0 0 0 0 0 1 0 0 0 0 0 0 0 0 0 0 1 1 0 1 2 0 0 0 1 0 0 1 0 0 0 0 0 0 0 0 0 0 0 1 00 0 0 0 0 2 ? 0 0 0 2 0 0 0 ? 0 0 0 0 0 0 0 0 0 0 0 1 0 0 0 0 0 0 0 0 0 0 0 0 1 0 0 0 0 0 0 0 0 0 0 0 0 0 0 0 0 1 0 0 0 0 0 0 0 0 0 0 0 0 0 0 0 0 0 0 0 0 0 0 0 0 0 0 0 0 0 0 1 0 0 1 0 0 0 0 1 0 0 0 0 0 0 0 0 0 0 0 1 0 0 0 0 0 0 1 0 0 0 0 0 0 0 0 0 0 0 0 0 0 ? 0 0 0 ? 0 0 0 0 0 0 0 1 0 ? ? ? ? 0 0 0 0 0 0 0 0 0 0 0 0 0 0 0 0 0 0 0 0 0 0 0 0 0 0 0 0 1 0 0 0 1 0 1 0 0 0 0 0 0 0 0 0 0 0 0 0 0 0 0 0 0 0 0 0 0 1 0 0 1 0 0 0 0 0 0 0 1 0 0 0 0 0 0 0 0 0 1 0 0 0 0 0 0 0 1 0 0 0 0 0 0 0 0 0 0 1 0 0 0 0 0 1 0 0 0 0 0 0 0 0 0 0 0 0 1 0 0 0 0 0 0 0 0 0 0 0 0 0 1 0 0 0 1 0 0 0 0 0 0 1 0 0 0 0 0 0 0 0 0 0 0 0 0 0 0 0 0 0 0 0 0 0 0 0 0 0 0 0 0 0 0 0 0 0 0 0 0 0 0 0 0 0 0

Sinraptor 0 0 0 0 0 0 0 0 0 0 0 0 1 0 0 0 0 0 ? 0 0 0 0 0 0 ? 1 0 0 0 0 0 0 0 0 0 0 1 1 0 1 2 0 0 0 1 0 0 0 0 0 0 0 0 0 0 0 0 0 0 0 00 0 0 0 0 2 0 0 0 0 2 ? 0 0 ? 0 0 ? 0 0 ? 0 0 0 0 0 1 0 0 0 0 0 0 0 0 0 0 0 0 1 0 0 ? 0 0 0 0 0 0 0 0 0 0 0 0 0 1 0 0 0 1 0 0 0 ? 0 ? 0 0 0 0 0 0 0 0 0 0 0 0 0 0 0 0 0 0 0 0 1 0 0 1 0 0 0 0 1 0 0 0 0 0 0 0 0 0 ? 0 1 1 0 0 0 0 0 1 0 0 0 0 0 0 0 0 0 ? 0 0 0 0 ? ? ? ? ? ? ? ? ? ? ? ? ? ? ? ? ? ? ? ? 0 0 0 0 0 0 ? ? ? ? ? ? 0 ? ? ? ? ? ? ? ? ? ? ? ? ? ? ? ? ? ? ? ? ? 0 ? ? ? 0 0 0 0 0 0 0 0 ? ? ? ? ? ? ? ? ? ? ? ? 1 0 ? ? ? 0 0 1 ? ? ? 0 0 ? ? ? 0 0 1 0 0 0 ? 0 0 0 1 0 0 ? 0 0 0 0 0 0 0 1 0 0 0 0 0 0 0 0 0 0 0 0 0 0 0 0 0 0 1 0 0 0 0 0 0 0 0 0 0 0 0 ? 1 0 0 0 1 0 0 0 0 0 0 1 0 0 0 0 0 0 0 0 0 0 0 0 0 0 0 0 0 0 0 0 0 0 0 0 0 ? 0 0 0 0 0 0 0 0 0 0 0 0 0 0 0 0 0

Dilong 0 0 0 0 0 1 0 0 0 0 0 0 1 0 0 0 0 0 ? 0 0 0 0 0 0 1 1 0 1 0 ? ? 0 0 0 1 0 0 0 0 1 2 1 0 0 1 0 0 0 0 0 0 0 0 0 0 0 0 ? 0 ? ?1 ? 0 ? 0 ? ? 0 0 0 1 1 ? ? ? ? ? ? ? ? 0 ? 0 0 ? ? ? ? 0 ? ? ? ? 0 ? ? ? ? ? ? ? ? ? 0 0 0 0 0 0 ? ? 0 0 0 0 0 1 0 ? ? ? ? ? 0 ? ? ? ? 0 0 0 0 0 1 0 0 1 0 0 0 0 1 0 0 0 0 0 1 1 0 1 0 ? ? ? 1 1 0 0 0 0 0 ? 0 0 0 0 1 1 ? 0 ? 1 0 ? 0 0 0 ? ? ? ? ? ? ? 0 0 ? 0 ? ? ? 0 ? 0 0 0 ? 1 ? 1 ? ? 0 ? ? ? ? ? 1 0 0 0 0 0 ? 0 0 ? ? 0 1 1 1 0 0 0 0 0 1 0 0 0 ? ? 1 0 ? ? ? ? ? 0 1 0 0 ? 0 0 0 ? 0 0 1 0 0 0 0 0 0 1 0 0 0 1 0 0 1 0 0 1 0 0 0 0 0 0 0 0 0 0 0 0 0 ? ? ? ? ? ? 0 0 ? ? 0 0 0 0 ? 1 0 0 0 ? ? 0 0 0 0 0 1 0 1 0 0 ? ? ? 0 ? 0 0 ? 1 0 0 0 0 1 ? 0 1 ? 0 0 0 ? 1 0 0 0 ? 0 0 0 0 ? ? 0 1 0 0 0 1 0 1 0 1 0 0 ? 0 0 0 ? 1 0 0 0 ? ? ? ? 1 0 ? 0 0 0 0 0 0 0 0 ? 0 0 0 ? 0 0 0

Guanlong 0 0 0 0 0 1 0 0 0 0 0 0 1 0 0 0 0 1 ? 0 0 0 0 0 0 1 1 0 1 0 1 0 0 0 0 1 0 0 0 0 ? 2 1 0 0 1 0 1 0 0 0 0 0 0 0 0 0 0 0 0 ? 0? ? 0 ? ? ? ? 0 0 0 1 1 ? ? ? ? ? ? 0 ? 0 0 0 0 0 ? ? 0 0 0 ? 0 0 0 1 ? ? ? ? ? ? ? ? 0 0 0 0 0 0 0 0 0 0 0 1 0 ? 0 0 0 1 0 0 0 ? 0 ? 0 0 0 0 0 0 1 0 0 1 0 0 0 0 1 0 0 0 0 0 1 1 0 1 0 0 0 ? ? ? ? ? ? ? 0 0 ? 0 ? 0 ? ? 0 0 ? 1 0 ? 0 ? ? 0 ? ? 0 0 0 ? 0 ? ? ? ? ? ? 0 ? ? ? ? ? ? 0 0 ? ? ? ? ? ? ? ? 1 ? 0 ? ? 0 ? ? 0 ? ? 0 0 1 1 0 0 0 0 0 1 0 0 0 ? 1 1 ? ? ? 1 1 1 0 1 0 0 1 0 ? 0 ? 0 0 0 ? 0 0 1 0 0 1 0 0 0 1 0 0 0 0 0 1 0 0 0 0 0 0 0 0 0 0 0 0 0 3 1 0 0 0 0 0 0 0 1 0 0 0 0 0 1 0 0 0 1 1 0 0 0 ? ? 1 0 0 0 0 1 0 0 0 0 0 0 ? 1 0 0 ? 0 0 ? 0 1 ? 0 0 0 ? 1 0 0 0 ? 0 ? ? 0 ? ? ? ? 0 0 0 1 ? 1 0 1 0 0 0 0 0 0 ? ? 0 ? 0 ? ? 0 0 1 ? 0 0 0 0 0 0 0 0 0 0 ? ? 0 0 0 0 0

Raptorex 0 0 0 0 0 1 0 0 0 0 ? 0 0 ? ? 0 1 0 ? 0 0 0 0 1 1 1 1 0 1 0 ? 0 0 0 1 1 0 1 0 0 1 2 1 0 1 1 ? ? ? 0 0 0 0 0 0 0 0 ? ? ? ? ?? ? ? ? ? 2 ? 0 0 ? 2 ? ? ? ? ? ? ? ? ? 0 ? ? ? ? ? ? ? ? ? ? ? ? ? ? ? ? ? ? ? ? ? ? 0 0 0 0 0 0 0 ? 0 0 0 0 0 1 1 0 0 1 ? ? 0 ? 0 ? ? 0 0 0 0 0 1 0 0 1 0 0 0 0 0 0 0 0 0 0 1 ? ? 1 ? ? 0 ? 1 1 0 0 ? 0 0 ? ? 0 0 0 1 1 0 1 ? 0 0 ? 0 ? ? ? 0 ? ? 1 ? ? 0 ? ? ? ? 0 ? ? ? ? ? ? ? ? ? ? ? ? ? ? ? ? ? ? 1 0 1 0 0 0 1 0 0 0 0 0 0 0 1 1 0 0 0 0 0 0 0 0 ? 0 1 ? 0 ? ? ? ? 0 1 ? 0 ? 0 ? ? ? ? ? ? ? ? 0 ? ? 0 1 0 ? 0 ? 0 ? ? ? ? ? ? 0 ? 0 ? 0 ? 0 ? 0 ? ? 0 3 1 0 0 ? ? 0 0 0 1 0 0 ? 0 ? 1 1 ? 0 ? ? 0 0 ? ? ? ? ? 0 ? 0 ? 0 0 1 0 0 0 ? 1 0 ? ? 0 1 ? ? 1 ? 0 1 0 ? 1 ? 0 0 0 ? ? ? ? ? ? 0 1 0 0 ? 1 ? ? ? ? ? ? ? ? ? 0 ? 0 1 ? 0 ? ? ? ? ? ? ? ? ? ? ? ? ? ? ? ? 0 0 ? ? ? ? ?

Gorgosaurus 0 0 0 0 0 1 0 0 0 0 0 0 0 0 0 0 1 0 ? 1 0 0 0 0 1 1 1 0 1 0 1 0 0 0 1 1 0 1 0 0 1 2 1 0 1 0 0 0 0 0 1 0 0 0 0 0 0 1 0 0 0 01 0 0 0 0 2 ? 0 0 0 2 1 ? 0 ? ? ? 0 0 ? 0 0 0 0 ? 0 1 0 0 0 ? 0 0 1 1 ? ? 0 0 1 ? ? 0 1 0 0 0 0 0 0 0 0 0 0 0 0 1 1 0 0 1 0 0 0 0 0 0 1 0 0 0 0 0 1 0 0 1 0 0 0 0 0 0 0 0 0 0 1 0 0 1 0 0 0 0 1 0 0 0 0 0 0 0 ? 0 0 0 ? 0 0 1 0 0 0 1 0 0 0 0 0 0 0 1 0 0 0 0 0 0 ? 0 0 0 ? 0 0 0 0 1 0 0 1 0 ? ? ? ? 0 0 1 0 1 0 0 0 1 0 0 0 0 0 0 0 0 1 0 0 0 0 0 1 0 0 0 0 1 0 0 0 0 1 1 0 1 0 0 1 0 0 0 1 0 0 1 0 0 0 1 0 0 1 0 0 0 ? 0 ? ? 1 ? ? ? 0 0 0 0 0 0 0 0 0 0 ? 0 3 1 0 0 0 0 0 0 0 1 0 0 0 0 0 1 1 0 0 1 1 0 0 0 0 0 1 0 0 0 0 1 0 0 1 0 0 0 1 1 0 0 0 0 1 0 1 0 0 0 1 0 0 1 0 0 0 0 0 0 0 0 0 1 0 1 0 0 0 1 0 1 0 1 0 0 0 0 0 0 0 0 2 1 0 0 0 0 0 1 0 0 0 0 0 0 0 0 0 0 0 0 0 0 0 0 0 0

Daspletosaurus 0 0 0 0 1 1 0 0 0 0 0 0 0 0 0 ? 0 0 ? 1 0 0 0 1 1 1 1 0 1 0 1 0 0 0 1 1 0 1 0 0 1 2 1 0 1 1 0 0 0 0 1 0 0 0 0 0 0 1 0 0 0 01 0 0 0 0 2 0 0 0 0 2 1 0 0 ? 0 0 0 0 ? 0 0 0 0 0 0 0 0 0 0 0 0 0 1 1 0 0 0 0 1 0 0 0 1 0 0 0 0 0 0 0 0 0 0 0 0 1 1 0 0 1 0 0 0 0 0 ? 1 0 0 0 0 0 1 0 0 1 0 0 0 0 0 0 0 0 0 0 1 0 0 1 0 0 0 0 1 0 0 0 0 0 1 0 ? 0 0 0 1 0 0 1 0 0 0 1 0 ? 0 0 0 ? 0 ? 0 0 0 0 0 0 0 0 0 0 ? 0 0 0 0 1 ? ? ? ? ? ? ? ? 0 0 1 0 1 0 0 0 1 0 0 0 0 0 0 0 0 1 0 0 0 0 0 1 0 0 0 0 1 0 0 ? 0 1 1 0 1 0 0 1 0 0 0 ? 0 0 1 0 ? ? ? ? ? 1 0 0 ? ? 1 ? ? 1 ? ? ? 0 0 0 ? ? 0 ? 0 ? 0 ? 0 3 1 0 0 0 0 0 0 0 1 0 0 0 0 0 1 1 0 0 1 1 0 0 0 0 0 1 1 0 0 0 1 0 0 1 0 0 0 1 1 0 0 0 0 1 0 1 0 ? 0 1 0 0 1 0 0 0 0 0 0 0 0 0 ? 0 1 0 0 0 1 0 1 0 0 0 0 ? 0 0 0 0 0 2 1 0 0 ? 0 0 1 ? 0 0 0 0 0 0 0 0 0 0 0 0 0 0 0 0 0

Tyrannosaurus 0 0 0 0 1 1 0 0 0 0 0 0 0 0 0 0 1 0 ? 1 0 0 0 1 1 1 1 0 0 0 1 0 0 0 1 1 0 1 0 0 1 2 1 0 1 0 0 0 0 0 1 0 0 0 0 0 0 1 0 0 0 01 0 0 0 0 2 0 0 0 0 2 1 0 0 ? 0 0 0 0 ? 0 0 0 0 0 0 1 0 0 0 2 0 0 1 1 0 0 0 0 1 ? 0 0 1 0 0 0 0 0 0 0 0 0 0 0 0 1 1 0 0 1 0 0 0 0 0 0 1 0 0 0 0 0 1 0 0 1 0 0 0 0 0 0 0 0 0 0 1 0 0 1 0 0 0 0 1 0 0 0 0 0 1 0 0 0 0 0 1 0 0 1 0 0 0 1 0 0 0 0 0 0 0 1 0 0 0 0 0 0 ? 0 0 0 ? 0 0 0 0 1 ? ? 1 ? ? ? ? ? 0 0 1 0 1 0 0 0 1 0 0 0 0 0 0 0 0 1 0 0 0 0 0 1 0 0 0 0 1 0 0 ? 0 1 1 0 1 0 0 1 0 0 0 1 0 0 1 0 0 0 1 0 0 1 0 0 0 ? 0 ? ? 1 ? ? ? 0 0 0 0 0 ? 0 ? 0 ? ? 0 3 1 0 0 0 0 0 0 0 1 0 0 0 0 0 1 1 0 0 1 1 0 0 0 0 0 1 1 0 0 0 1 0 0 1 0 0 0 1 1 0 0 0 0 1 0 1 0 0 0 1 0 0 1 0 0 0 0 0 0 0 0 0 1 0 1 0 0 0 1 0 1 0 0 0 0 0 0 0 0 0 0 2 1 0 0 0 0 0 1 ? 0 0 0 0 0 0 0 0 0 0 0 0 0 0 0 0 0

Tanycolagreus ? ? ? ? ? 0 0 0 0 0 0 ? ? 0 ? ? ? ? ? ? ? ? ? ? ? ? ? ? ? ? ? ? 0 ? 0 0 ? ? 0 0 0 2 ? 0 0 1 0 ? ? ? ? ? ? ? ? ? 0 ? ? ? ? ?0 0 ? ? ? ? ? ? ? ? ? ? ? ? ? ? ? ? ? ? ? ? ? ? ? ? ? ? ? ? ? ? ? ? ? ? ? ? ? ? ? ? ? ? ? ? ? ? ? ? ? ? ? ? ? ? ? ? ? ? ? ? ? ? ? ? ? 0 0 0 0 0 ? 0 0 0 1 ? ? ? ? ? 0 ? ? ? ? ? ? ? 1 ? ? ? ? ? ? ? ? ? ? ? ? ? ? ? ? ? 1 ? 0 0 1 0 1 0 0 0 0 ? ? ? ? ? ? ? 0 0 ? ? ? ? 0 ? ? ? ? ? ? ? ? ? ? ? ? ? ? ? ? 0 0 0 0 0 0 1 0 0 0 0 0 0 1 1 0 0 0 0 0 1 1 0 0 ? 1 1 0 0 ? 1 0 0 0 1 0 0 0 0 0 0 1 0 0 1 0 0 0 1 0 0 1 0 0 1 1 0 0 1 0 0 1 0 0 0 0 1 1 0 0 0 0 0 0 ? ? ? ? ? ? ? ? ? ? ? ? ? ? ? ? ? ? ? ? ? ? 0 ? 0 0 0 1 0 1 ? ? ? ? ? ? ? ? ? ? ? ? ? ? ? ? ? ? 1 0 0 0 0 0 1 0 ? 0 0 0 0 0 0 0 0 0 1 0 0 0 1 0 1 0 1 0 0 ? 0 0 0 0 0 0 0 0 0 0 0 0 1 0 0 0 0 0 0 0 0 0 0 1 0 0 0 0 0 0 0

Coelurus ? ? ? ? ? ? ? ? ? ? ? ? ? ? ? ? ? ? ? ? ? ? ? ? ? ? ? ? ? ? ? ? ? ? ? ? ? ? ? ? ? ? ? ? ? ? ? ? ? ? ? ? ? ? ? ? ? ? ? ? ? ?? ? ? ? ? ? ? ? ? ? ? ? ? ? ? ? ? ? ? ? ? ? ? ? ? ? ? ? ? ? ? ? ? ? ? ? ? ? ? ? ? ? ? ? ? 0 ? 0 0 ? ? 0 1 0 ? ? ? ? ? ? ? ? ? ? ? ? ? ? ? ? ? ? ? ? ? ? ? ? ? 0 ? ? 0 0 ? ? ? ? ? ? ? ? 0 ? ? ? ? 0 ? 0 0 0 0 0 0 ? 1 0 1 ? 0 0 1 ? 1 1 0 0 0 ? ? ? ? ? ? 0 ? ? ? ? ? 0 2 ? ? ? ? ? ? ? ? ? ? ? ? ? ? ? ? ? 0 ? 0 0 0 ? ? ? ? ? ? ? 1 1 0 0 0 0 0 1 1 0 0 ? 1 1 0 0 ? 1 0 1 0 1 ? ? 0 0 ? ? 1 ? ? ? 0 ? 0 1 ? 0 ? 0 0 1 1 0 ? 1 0 ? ? 0 ? ? ? ? ? ? ? ? ? ? ? ? ? ? ? ? ? ? ? ? ? ? ? ? ? ? ? ? ? ? 0 ? 0 0 0 0 0 0 1 0 1 ? ? ? ? ? ? ? ? ? ? ? ? ? ? ? ? ? ? 1 0 0 0 0 1 1 0 0 0 ? ? ? ? ? 0 0 ? 1 1 0 0 1 0 1 0 1 0 ? ? ? ? 0 ? 0 ? 0 0 0 0 0 ? ? ? ? ? ? ? ? ? ? ? ? ? ? ? ? ? ? ? ?

Sinocalliopteryx 0 0 0 ? 0 0 0 0 0 0 0 0 1 1 0 0 1 0 ? 1 0 0 1 1 0 1 0 ? ? ? ? 0 0 0 0 0 0 0 0 0 0 0 ? 0 0 1 1 0 ? 0 0 1 0 0 0 0 ? 0 ? ? ? 0? 0 ? ? 1 ? ? 0 ? 0 ? ? ? ? ? ? ? ? 2 ? ? ? ? ? ? ? ? ? ? ? ? ? ? 0 0 ? ? ? ? ? ? ? 1 0 0 0 0 0 0 ? 0 0 0 0 1 0 0 0 ? 0 ? 1 ? 0 ? ? ? 0 0 0 0 0 0 3 0 0 1 0 0 0 0 1 0 0 0 0 0 1 ? ? 1 ? 0 ? ? ? 1 ? 0 ? 0 0 0 0 0 0 0 0 1 0 0 ? 1 1 ? ? ? 0 ? ? ? ? ? ? 0 0 0 ? 0 ? 0 ? 2 ? 0 0 0 0 0 0 0 0 0 ? ? ? ? ? 0 0 0 0 ? 0 0 0 0 0 ? ? 0 0 1 0 0 ? 0 ? 0 1 0 0 0 0 0 0 ? 0 ? 1 0 1 0 1 0 ? 0 0 ? 0 ? 0 0 1 0 0 0 0 0 1 1 0 0 0 1 0 0 1 0 0 0 0 0 0 0 0 0 0 0 0 0 0 0 0 3 1 0 0 0 ? 0 0 0 1 0 0 ? 0 ? 0 0 0 0 1 ? 0 0 ? ? ? 0 0 ? 0 0 0 0 0 1 0 0 0 ? 1 0 0 0 0 1 0 0 1 ? ? ? ? ? ? ? 0 ? ? 0 ? ? 0 ? ? ? ? 0 ? 0 1 ? 1 0 1 0 0 0 0 0 0 ? ? 0 0 0 ? ? ? 0 ? 0 0 ? 0 0 0 0 0 0 0 ? 0 0 0 0 0 0 0

Huaxiagnathus 0 0 0 ? 0 ? 0 0 0 0 0 0 1 1 0 0 1 0 ? 1 0 0 1 1 0 ? 1 0 ? 1 ? ? ? 0 0 ? 0 0 0 0 0 ? ? 0 0 1 ? 0 ? 0 0 1 0 0 0 ? ? ? ? ? ? ?? ? 1 ? 1 2 ? 0 ? 0 ? ? ? ? ? ? ? ? ? ? ? ? ? ? ? ? ? ? ? ? ? ? ? ? ? ? ? ? ? ? ? ? ? 0 0 0 0 0 0 0 ? 0 0 0 1 ? ? ? ? ? ? 1 ? 0 ? ? ? ? ? 0 ? 0 1 0 0 0 1 0 0 0 0 1 0 0 0 0 0 1 ? 0 1 0 ? 0 ? ? 1 0 0 ? 0 ? ? ? 0 0 0 ? 1 ? ? ? 1 1 ? ? ? 0 0 ? ? ? ? ? ? 0 0 0 0 ? 0 ? 2 ? 0 0 0 0 0 0 0 0 0 ? ? ? ? 0 0 0 0 0 0 0 0 0 0 0 0 0 0 0 1 0 0 ? 0 ? 0 1 0 0 0 0 0 1 ? 0 ? 1 0 1 0 1 0 0 0 0 0 0 1 0 0 1 0 0 0 1 0 1 1 0 0 0 1 0 0 1 0 0 ? ? 0 0 0 0 0 0 0 0 0 0 0 0 0 0 0 0 1 0 0 0 0 ? ? 0 ? 0 1 0 0 ? 0 1 0 0 0 ? ? ? 0 0 1 0 0 ? 0 0 1 0 0 0 ? 1 0 0 0 0 1 0 0 1 ? 0 ? ? ? ? ? 0 0 ? 0 ? 0 0 ? ? 0 1 0 0 0 1 0 1 0 1 0 0 0 0 0 0 0 0 0 0 0 0 ? 0 0 1 0 0 ? 0 0 0 0 0 0 0 0 0 0 0 0 0 0 0

Sinosauropteryx 0 0 0 1 0 ? 0 0 0 0 0 0 1 1 0 0 0 0 ? 1 0 0 1 1 0 1 1 0 1 1 ? ? ? 0 0 0 0 0 0 0 0 0 0 0 0 1 ? 0 ? 0 0 1 0 0 0 ? ? 0 0 ? 0 01 0 1 ? 1 ? ? 0 ? 0 ? ? ? ? ? ? ? ? ? ? ? ? ? ? ? ? ? ? ? ? ? ? ? ? ? ? ? ? ? ? ? ? ? 0 0 0 0 0 0 0 ? 0 ? 0 1 0 ? ? ? ? ? 1 ? 0 ? 0 ? ? ? 0 0 0 1 0 0 0 0 0 0 0 0 1 0 0 0 0 0 1 ? 0 1 0 ? 0 ? ? 1 0 0 ? 0 0 ? 0 0 0 0 0 1 ? 0 ? 1 1 ? 0 ? ? 0 0 ? ? ? ? 0 0 0 ? 0 ? 0 0 2 ? 1 0 0 0 0 0 0 0 0 0 ? ? ? ? ? 1 0 0 0 ? 0 0 0 0 0 0 0 0 0 0 0 ? 0 ? 0 0 0 0 0 0 0 1 ? 0 ? 1 0 1 0 1 0 0 0 0 0 0 ? 0 0 0 0 ? 0 0 0 1 1 0 0 0 1 0 0 1 0 0 0 0 0 0 0 1 0 0 1 1 0 0 ? 0 0 0 0 0 ? ? 0 0 0 ? ? ? ? 0 1 0 2 ? 0 1 0 0 0 ? ? ? 0 0 1 1 0 0 0 0 1 0 0 0 ? 1 0 0 0 0 1 0 0 1 ? 0 0 0 0 ? ? 0 0 ? 0 ? ? 0 0 ? 0 1 0 0 0 1 ? 1 0 1 0 0 0 0 0 0 0 0 0 0 0 0 ? ? 0 1 0 0 0 0 0 0 0 0 0 0 ? 0 0 0 0 0 0 0

Compsognathus 0 0 0 1 0 0 0 0 0 0 0 0 1 1 0 0 0 0 ? 1 0 0 1 1 0 1 1 0 1 1 ? 0 0 0 0 0 0 0 0 0 0 0 0 0 0 1 1 0 1 0 0 1 0 0 0 0 0 ? ? ? ? ?? ? 1 ? 1 2 0 0 ? 0 0 ? ? ? ? ? ? ? ? ? ? ? ? ? ? ? ? ? ? ? ? ? 0 0 ? ? ? 0 ? ? ? ? ? 0 0 0 0 0 0 0 ? 0 0 0 1 0 0 0 0 0 0 1 ? 0 0 0 ? 0 0 0 0 0 1 0 0 0 0 0 0 0 0 1 0 0 0 0 0 1 ? 0 1 0 0 0 ? 0 1 0 0 ? 0 0 0 0 0 0 0 ? 1 0 0 0 1 1 ? 0 ? 0 0 0 ? 0 0 0 0 0 0 ? 0 ? 0 ? 2 ? 1 0 0 0 0 0 0 0 0 ? ? ? ? 0 0 1 0 0 ? 0 ? 0 0 0 0 0 0 0 1 0 0 0 0 0 0 1 1 0 0 0 0 1 ? 0 ? 1 0 ? 0 1 0 ? 0 0 0 0 1 0 0 0 0 0 0 1 0 1 1 0 0 0 1 0 ? 1 0 ? ? ? 1 0 1 0 ? 0 1 1 0 0 ? 0 ? 0 ? 0 ? ? 0 0 ? ? 0 0 ? 0 ? ? ? ? ? 0 0 0 0 ? ? ? 0 0 1 1 0 ? 0 0 1 0 0 0 ? 1 0 0 0 0 1 0 0 1 ? 0 0 0 ? ? ? 0 0 ? ? ? 0 0 0 ? 0 1 0 0 0 1 0 1 0 1 0 0 0 0 0 0 0 0 0 0 0 0 0 0 0 1 0 0 0 0 0 0 0 0 0 0 0 0 0 0 0 0 0 0

Juravenator 3 0 0 1 0 ? 0 0 0 0 0 0 0 1 0 0 0 0 ? 1 0 0 1 1 0 ? 1 0 1 1 ? ? 0 0 0 0 0 0 0 0 ? 0 ? 0 0 1 1 0 ? 0 0 1 0 ? 0 0 ? 0 0 0 0 0? 0 1 ? 1 2 ? 0 0 0 0 ? ? ? ? ? ? ? ? ? 0 ? ? ? ? ? ? ? ? ? ? ? ? ? ? ? ? ? ? ? ? ? ? 0 ? 0 0 0 0 ? 0 0 0 0 1 0 ? ? ? ? ? 1 ? 0 0 0 0 0 0 0 0 0 ? 0 0 0 0 0 0 0 0 1 0 0 0 ? ? 1 ? 0 1 0 ? 0 ? ? 1 ? ? ? ? ? ? ? 0 ? 0 ? ? ? ? ? ? 1 ? ? 0 0 ? ? ? ? ? ? 0 0 0 ? 0 ? 0 ? 2 ? 1 0 0 0 ? 0 0 0 0 ? ? ? ? ? ? 1 0 0 0 0 0 0 0 0 ? ? 0 0 1 0 0 0 0 ? 0 ? 0 0 0 ? 0 0 ? 0 ? ? ? ? ? 1 0 ? 0 ? ? 0 ? ? 0 0 ? 0 0 1 0 0 1 0 0 0 1 0 0 0 0 0 0 0 1 0 0 0 0 0 1 1 0 0 0 0 0 0 0 0 ? ? 0 0 0 1 ? 0 ? ? ? ? ? 0 ? ? ? ? ? ? ? ? ? ? ? ? 0 ? ? ? ? ? ? ? ? ? ? ? ? ? ? ? ? 1 ? 0 0 0 ? 0 0 0 0 ? ? ? ? 0 ? ? ? ? ? 0 0 1 ? 1 0 ? 0 0 0 0 0 0 ? ? ? 0 0 ? ? ? 0 1 0 0 0 0 0 0 0 0 0 0 ? 0 0 0 0 0 0 0

Scipionyx 3 0 0 1 0 0 0 0 0 0 0 0 1 1 0 0 0 0 ? 1 0 0 1 1 0 1 0 ? ? 1 ? ? 0 ? 0 ? 0 0 ? 0 0 0 0 0 0 1 1 0 1 0 0 1 0 0 0 0 0 0 0 0 0 0? 0 1 ? 1 2 0 0 ? 0 ? ? ? ? 0 1 0 ? ? ? 0 ? ? ? ? ? ? ? ? ? ? ? ? ? ? 0 1 0 0 ? 0 ? ? 0 0 0 0 0 0 0 0 0 0 0 1 0 0 0 ? 0 0 1 ? 0 ? 0 ? ? ? 0 0 0 1 0 0 0 0 0 0 0 0 1 0 0 0 0 0 1 ? 0 1 ? 0 0 1 0 3 0 0 ? 0 0 0 0 0 0 0 0 1 0 0 0 1 2 ? 0 ? ? 0 0 0 ? 0 0 ? 0 0 ? ? ? 0 ? ? ? 1 0 0 ? ? 0 0 0 0 ? ? ? ? 0 0 ? 0 0 0 0 0 0 0 0 0 0 0 ? 1 0 0 0 0 ? 0 1 0 0 0 1 0 0 ? 0 ? 1 0 1 0 1 0 0 0 0 0 0 1 1 0 0 0 0 0 1 0 0 1 0 0 0 1 0 0 0 0 0 2 0 1 0 0 0 0 0 1 1 0 0 0 ? 0 0 ? 0 ? ? 0 0 0 1 ? 0 ? ? 1 ? ? ? 0 1 0 0 0 0 0 0 0 0 1 ? 0 ? ? ? 1 0 ? 0 ? ? ? ? ? 0 ? ? 0 ? ? 0 0 0 ? 0 0 0 0 ? 0 0 ? ? ? ? ? ? ? ? ? ? ? ? ? ? ? ? ? ? ? ? ? ? ? ? ? ? ? ? ? ? ? ? ? ? ? ? ? ? ? ? ? ? ? ? ? ? ? ?

Harpymimus 1 0 0 1 0 0 0 0 0 0 2 0 0 1 2 0 2 0 ? 1 0 0 0 1 0 0 ? ? ? 1 ? ? 0 0 0 0 0 0 0 0 0 0 0 0 0 1 1 ? ? 1 0 1 0 ? 0 ? ? ? ? ? 0 0? ? 1 1 1 ? 0 0 ? 0 ? ? ? ? ? ? ? ? ? ? ? ? ? ? ? ? ? ? ? ? ? ? ? ? ? ? ? ? 0 ? ? ? ? 0 0 0 1 0 0 0 ? 0 0 0 1 0 0 0 1 ? ? 0 0 0 0 0 ? ? ? ? 0 1 ? ? ? ? ? 1 2 1 0 2 2 0 0 0 ? ? ? ? ? 1 0 0 ? ? 1 ? 0 ? 0 0 0 1 0 1 0 0 1 0 0 0 1 0 1 0 ? 0 0 1 ? 1 0 0 0 0 0 ? 1 0 ? 0 0 ? ? 0 0 ? 1 ? ? ? ? ? ? ? ? ? ? 0 0 0 0 ? 0 ? 0 ? ? ? ? 0 ? 1 0 0 0 0 0 ? 1 0 0 1 0 1 0 0 0 0 0 1 0 1 0 0 0 0 0 0 1 0 0 0 0 0 0 0 0 0 1 0 0 0 0 0 0 0 0 0 2 0 0 2 1 0 0 0 0 1 0 0 1 ? ? 1 0 0 ? ? 0 0 0 1 ? 0 0 0 ? 0 ? 1 ? ? ? 0 0 0 0 0 ? ? ? ? 0 ? 0 0 ? 0 ? ? ? ? ? ? ? ? ? ? ? ? ? ? ? ? ? ? ? 0 ? ? ? ? ? ? ? ? ? ? ? 0 0 ? 0 1 0 1 0 ? ? ? ? 0 0 0 1 0 0 0 0 0 0 ? ? 0 0 0 0 0 0 0 0 0 0 ? ? 0 ? 0 0 1

Deinocheirus ? ? ? ? ? ? ? ? ? ? ? ? ? ? ? ? ? ? ? ? ? ? ? ? ? ? ? ? ? ? ? ? ? ? ? ? ? ? ? ? ? ? ? ? ? ? ? ? ? ? ? ? ? ? ? ? ? ? ? ? ? ?? ? ? ? ? ? ? ? ? ? ? ? ? ? ? ? ? ? ? ? ? ? ? ? ? ? ? ? ? ? ? ? ? ? ? ? ? ? ? ? ? ? ? ? ? ? ? ? ? ? ? ? ? ? ? ? ? ? ? ? ? ? ? ? ? ? ? ? ? ? ? ? ? ? ? ? ? ? ? ? ? ? ? ? ? ? ? ? ? ? ? ? ? ? ? ? ? ? ? ? ? ? ? ? ? ? ? ? ? ? ? ? ? ? ? ? ? ? ? ? ? ? ? ? ? ? ? ? ? ? ? ? ? ? ? ? ? ? ? ? ? ? ? ? ? ? ? ? ? 0 ? 0 0 0 1 ? 0 ? 0 ? ? 0 ? 1 0 0 0 ? 0 ? 1 0 0 ? 0 1 0 0 ? ? ? ? 0 1 1 1 1 0 0 ? 1 0 0 0 0 1 0 0 0 ? 1 0 0 0 0 0 1 0 0 0 1 0 0 0 1 0 0 0 0 0 0 0 1 ? ? ? ? ? ? ? ? ? ? ? ? ? ? ? ? ? ? ? ? ? ? ? ? ? ? ? ? ? ? ? ? ? ? ? ? ? ? ? ? ? ? ? ? ? ? ? ? ? ? ? ? ? ? ? ? ? ? ? ? ? ? ? ? ? ? ? ? ? ? ? ? ? ? ? ? ? ? ? ? ? ? ? ? ? ? ? ? ? ? ? ? ? ? ? ? ? ? ? ? ? ? ? ? ? ? ? ? ?

Shenzhousaurus 1 0 0 1 0 0 0 0 0 0 2 0 0 1 2 0 2 0 0 ? ? 0 0 1 0 0 1 0 0 ? 1 ? 0 0 0 0 0 ? 0 0 0 0 ? 0 0 ? ? ? 1 ? ? 1 ? 1 0 ? ? 0 1 0 ? ?? ? ? ? 1 ? ? 0 0 ? 0 0 ? ? ? ? ? ? ? ? ? ? ? ? ? ? ? ? ? ? ? ? ? ? ? ? ? ? 0 ? ? 0 ? 0 0 0 1 0 0 0 ? 0 0 0 1 0 ? ? 1 ? ? 0 0 0 ? 0 ? ? ? 0 ? 1 ? ? ? ? ? 1 2 1 0 2 2 0 0 0 ? ? ? ? 1 1 ? ? ? ? ? ? ? ? ? ? ? ? ? ? ? 0 1 ? 0 ? 1 0 ? 0 ? ? ? 0 ? ? ? 0 ? 0 0 0 1 0 0 ? ? ? 0 0 0 ? ? ? ? ? ? ? ? ? ? ? ? ? ? ? ? ? ? ? ? ? ? ? ? ? ? ? ? ? ? ? ? ? 1 ? ? ? ? ? ? ? ? ? ? ? ? ? ? ? ? ? 0 0 ? 0 ? 0 0 ? ? ? ? ? 1 ? 0 ? 0 1 1 0 0 0 2 0 0 2 1 0 0 0 1 0 0 ? 0 0 0 1 0 0 0 ? 0 0 0 1 0 0 ? 0 ? 0 0 1 0 0 0 0 0 0 1 0 0 0 0 0 0 0 0 ? 0 0 0 0 ? 1 0 0 ? 0 ? 0 0 ? ? 0 0 0 0 1 0 0 0 ? 0 ? ? ? ? ? ? ? ? ? ? ? ? ? ? ? ? ? ? ? ? ? ? ? ? ? ? ? ? ? ? ? ? ? ? ? ? ? ? ? ? ? ? ? ? ? ? ? ? ?

Pelecanimimus ? 0 0 1 0 0 0 0 0 0 2 0 0 1 2 0 1 0 0 0 1 0 0 1 0 0 1 0 0 1 ? ? 0 0 0 0 0 0 0 1 0 0 0 0 0 1 ? ? ? 1 0 1 0 1 0 0 ? ? ? ? ? 1? ? 1 1 1 ? ? 0 0 0 ? ? ? ? 1 1 ? ? ? ? 1 ? ? ? ? ? ? 0 0 0 2 ? ? ? ? ? ? ? 0 ? ? ? ? 0 0 0 0 0 0 ? ? 0 0 0 1 0 0 0 1 0 0 0 0 0 ? 0 ? ? ? 0 1 0 1 0 0 0 0 0 1 1 0 2 2 0 0 0 0 ? ? ? 0 1 0 ? ? ? 1 1 0 1 0 0 0 ? 0 ? 0 0 ? 0 ? ? ? ? ? ? ? 0 ? ? ? ? ? ? ? ? ? ? ? ? ? ? ? ? ? ? ? ? ? ? ? ? 1 0 1 0 0 ? ? ? ? ? ? ? 0 ? ? 2 ? ? 0 ? ? 1 2 ? ? ? ? ? 1 0 0 1 0 1 ? 0 ? 0 0 1 0 1 1 1 1 0 0 ? 1 1 1 0 0 1 0 0 0 0 1 0 0 0 0 1 1 0 0 0 2 0 0 2 1 0 0 0 1 2 0 0 1 ? ? ? ? ? ? ? ? ? ? ? ? 0 ? ? ? ? ? ? ? ? ? ? ? ? ? ? ? ? ? ? ? ? ? ? ? ? ? 0 ? ? ? ? ? ? ? ? ? ? ? ? ? ? ? ? ? ? ? ? ? ? ? ? ? ? ? ? ? ? ? ? ? ? ? ? ? ? ? ? ? ? ? ? ? ? ? ? ? ? ? ? ? ? ? ? ? ? ? ? ? ? ? ? ? ? ? ? ? ?

Garudimimus 1 0 0 1 0 0 0 0 0 0 2 0 0 1 2 0 2 0 ? 1 1 0 0 1 0 0 1 0 0 1 1 0 0 0 0 0 0 0 0 1 0 0 0 0 0 1 1 0 ? 1 0 1 0 1 0 0 0 0 ? 0 1 0? ? 1 1 1 0 0 0 0 0 0 0 0 ? ? ? ? ? ? 0 1 0 1 0 1 ? 0 0 0 0 ? 0 0 ? 1 ? 1 0 0 1 ? ? ? 0 0 0 1 0 0 0 0 0 0 0 1 0 0 0 1 0 0 0 0 0 0 0 2 0 0 0 1 1 ? ? ? ? ? 1 ? ? ? ? ? 1 ? ? ? ? ? ? ? ? ? ? 1 0 ? 1 1 ? 0 0 0 ? 0 ? 1 0 1 0 0 0 1 0 ? 0 ? ? 0 1 ? ? 0 ? ? 0 0 ? ? ? 0 ? ? ? ? ? ? ? ? ? ? ? ? ? ? ? ? ? ? ? ? ? ? ? ? ? ? ? 1 ? ? ? ? ? ? ? ? ? ? ? 1 ? 0 ? ? ? ? ? ? ? ? ? ? 1 ? ? ? ? ? ? ? ? ? ? ? ? ? ? ? ? ? ? ? ? ? ? ? ? ? ? ? ? ? ? ? ? ? ? ? ? ? ? ? 0 0 1 0 0 0 0 0 0 0 1 1 0 0 0 0 0 0 1 0 0 0 0 0 ? ? 0 ? ? 0 ? ? ? ? ? ? ? ? ? ? ? ? ? ? ? ? ? ? 1 0 0 0 0 0 1 0 ? 0 0 0 1 0 0 0 ? 0 1 0 0 0 1 0 1 0 1 0 0 ? 0 0 0 0 0 1 0 0 0 0 0 0 1 0 0 0 0 ? ? ? ? 0 0 0 0 0 ? ? 0 0 1

Archaeornithomimus ? ? 0 ? ? ? ? ? ? ? ? ? ? ? ? ? ? ? ? ? ? ? ? ? ? ? ? ? ? ? ? ? ? ? ? ? ? ? ? ? ? ? ? ? ? ? ? ? ? ? ? ? ? ? ? ? ? ? ? ? ? ?? ? ? ? ? ? ? ? ? ? ? ? ? ? ? ? ? ? ? ? ? ? ? ? ? ? ? ? ? ? ? ? ? ? ? ? ? ? ? ? ? ? ? ? ? ? ? ? ? ? ? ? ? ? ? ? ? ? ? ? ? ? ? ? ? ? ? ? ? ? ? ? ? ? ? ? ? ? ? ? ? ? ? ? ? ? ? ? ? ? ? ? ? ? ? ? 1 ? 1 0 0 0 0 1 0 ? ? 0 1 0 0 0 1 0 1 0 0 0 0 1 ? 1 0 0 ? 0 0 0 1 0 0 0 0 ? ? 0 0 ? ? ? ? ? ? ? ? ? ? ? ? ? 0 0 1 1 0 ? 0 2 1 1 0 ? ? 1 2 0 0 0 0 ? 1 0 0 1 0 1 0 0 ? ? ? ? 0 1 1 1 1 0 0 ? 1 0 0 0 0 1 ? ? 0 0 1 0 0 0 ? 1 ? 0 0 0 ? ? 0 1 1 0 0 0 1 2 0 0 ? ? ? ? ? ? 0 0 ? 0 0 1 1 0 0 0 0 0 0 1 0 0 0 0 0 0 1 0 0 0 0 ? 0 0 0 0 0 1 0 0 1 1 0 0 0 0 1 0 0 1 0 0 0 0 0 1 0 0 0 ? 0 0 0 0 0 1 0 1 0 0 0 ? 1 1 0 1 0 1 ? ? ? 0 0 0 1 1 0 0 1 0 0 ? ? ? ? ? ? ? ? ? ? ? 0 ? ? ? ? ? ? ?

Sinornithomimus 1 0 0 1 0 0 0 0 0 0 2 0 0 1 2 0 2 0 ? 1 ? 0 0 1 0 0 1 0 0 1 ? ? 0 0 0 0 0 0 0 1 0 0 0 0 0 1 1 1 1 1 0 1 0 1 0 0 0 0 1 0 1 00 0 1 1 1 0 ? 0 0 0 0 0 ? ? ? ? ? ? ? ? 1 ? ? ? ? ? 0 0 0 0 ? ? ? ? ? ? ? ? ? ? ? 0 ? 0 0 0 1 0 0 0 0 0 0 0 1 0 0 0 1 0 0 0 0 0 0 0 ? 0 0 0 1 1 ? ? ? ? ? 1 ? ? ? ? ? 1 ? ? ? ? ? ? ? ? ? 0 1 0 1 ? 1 1 0 0 0 ? 0 1 1 0 1 0 0 ? ? ? ? ? ? ? ? 1 ? 1 0 ? ? 0 0 0 ? ? 0 0 0 ? ? 0 0 0 1 0 0 ? 0 ? ? ? ? ? ? 0 0 0 1 0 0 1 0 2 1 1 0 1 1 1 2 0 0 0 0 1 1 0 0 1 0 ? ? 0 ? 0 0 1 0 1 3 1 1 1 0 0 ? 1 1 0 ? 1 0 0 0 0 1 0 0 0 0 1 1 0 0 0 2 0 0 1 1 0 0 0 1 1 0 0 1 0 0 1 0 0 0 0 0 0 0 1 1 0 0 0 0 0 0 1 0 0 0 0 0 0 1 0 0 0 0 0 0 0 0 0 0 1 0 0 ? 1 0 0 0 0 1 0 0 1 0 0 0 0 ? 1 0 0 0 ? 0 1 0 0 ? ? 0 1 0 0 0 1 1 1 0 2 0 1 ? ? ? 0 0 0 2 1 0 0 1 0 0 ? ? 0 ? ? 0 0 0 ? 0 0 0 ? ? 0 0 0 0 1

Gallimimus 1 0 0 1 0 0 0 0 0 0 2 0 0 1 2 0 2 0 ? 1 1 0 0 1 0 0 1 2 ? 1 1 0 0 0 0 0 0 0 0 1 0 0 0 0 0 1 1 1 1 1 0 1 0 1 0 0 0 0 1 0 1 10 0 1 1 1 0 0 0 0 0 0 0 0 0 ? 1 0 1 1 0 1 0 1 0 1 0 0 0 0 0 1 0 0 0 1 0 1 0 0 1 ? 0 1 0 0 0 1 0 0 0 0 0 0 0 1 0 0 0 1 0 0 0 0 0 0 0 2 0 0 0 1 1 ? ? ? ? ? 1 ? ? ? ? ? 1 ? ? ? ? ? ? ? ? ? 0 1 0 1 1 1 1 0 0 0 0 0 1 1 0 1 0 0 0 1 0 1 0 0 0 0 1 ? 1 0 0 0 0 0 0 1 0 0 0 0 ? 0 0 0 0 1 0 0 ? ? ? ? ? ? ? ? 0 0 0 1 1 1 ? 0 2 1 1 0 0 1 1 2 0 0 0 0 1 1 0 0 1 0 1 0 0 0 ? 0 1 0 1 1 1 1 1 0 0 1 0 0 0 0 1 0 0 0 0 1 0 0 0 0 1 1 0 0 0 2 0 0 1 1 0 0 0 1 1 0 0 1 0 0 1 0 0 0 0 0 0 0 1 1 0 0 0 0 0 0 1 0 0 0 0 0 0 1 0 0 0 0 0 0 0 0 0 0 1 0 0 1 1 0 0 0 0 1 0 0 1 0 0 0 0 0 1 0 0 0 1 0 1 0 0 0 1 0 1 0 0 0 1 1 1 0 2 0 1 ? ? ? 0 0 0 2 1 0 0 1 0 0 ? ? 0 0 0 1 0 0 0 0 0 0 ? ? 0 0 0 0 1

Struthiomimus 1 0 0 1 0 0 0 0 0 0 2 0 0 1 2 0 2 0 ? 1 1 0 0 1 0 0 1 2 ? 1 1 0 0 0 0 0 0 0 0 1 0 0 0 0 0 1 1 1 1 1 0 1 0 1 0 0 0 0 1 0 1 1? 0 1 1 1 0 0 0 0 0 0 0 0 0 ? 1 0 1 1 0 ? 0 ? 0 ? ? 0 0 0 0 1 0 0 0 1 0 1 ? 0 1 ? ? ? 0 0 0 1 0 0 0 0 0 0 0 1 0 0 0 1 0 1 0 0 0 0 0 2 0 0 0 1 1 ? ? ? ? ? 1 ? ? ? ? ? 1 ? ? ? ? ? ? ? ? ? 0 1 0 1 ? 1 1 0 0 0 0 0 1 1 0 1 0 0 0 1 0 1 0 0 0 0 1 0 1 0 0 0 0 0 0 1 0 0 0 0 ? 0 0 0 0 1 0 0 1 0 ? ? ? ? 0 ? 0 0 0 1 1 1 1 0 2 1 1 0 0 1 1 2 0 0 0 0 1 1 0 0 1 0 1 0 0 0 0 0 1 0 1 1 1 1 1 0 0 1 0 1 0 0 1 0 0 0 0 1 0 0 0 0 1 1 0 0 0 2 0 0 1 1 0 0 0 1 1 0 0 1 0 0 1 0 0 0 0 0 0 0 1 1 0 0 0 0 0 0 1 0 0 0 0 0 0 0 0 0 0 0 0 0 ? 0 0 0 1 0 0 1 1 0 0 0 0 1 0 0 1 0 0 0 0 0 1 0 0 0 1 0 ? 0 0 0 1 0 1 0 0 0 1 1 1 0 2 0 1 ? ? ? 0 0 0 2 1 0 0 1 0 0 1 ? 0 0 0 1 0 0 0 0 0 0 ? ? 0 0 0 0 1

Anserimimus ? ? ? ? ? ? ? ? ? ? ? ? ? ? ? ? ? ? ? ? ? ? ? ? ? ? ? ? ? ? ? ? ? ? ? ? ? ? ? ? ? ? ? ? ? ? ? ? ? ? ? ? ? ? ? ? ? ? ? ? ? ?? ? ? ? ? ? ? ? ? ? ? ? ? ? ? ? ? ? ? ? ? ? ? ? ? ? ? ? ? ? ? ? ? ? ? ? ? ? ? ? ? ? ? ? ? ? ? ? ? ? ? ? ? ? ? ? ? ? ? ? ? ? ? ? ? ? ? ? ? ? ? ? ? ? ? ? ? ? ? ? ? ? ? ? ? ? ? ? ? ? ? ? ? ? ? ? ? ? ? ? ? ? ? ? ? ? ? ? ? ? ? ? ? ? ? ? 0 ? ? ? ? ? ? ? ? ? ? ? ? ? ? ? ? ? ? ? ? 0 ? ? ? ? ? ? ? ? ? ? ? 3 ? ? 1 1 ? ? ? ? 1 1 ? ? ? ? 2 ? 0 ? 0 ? 1 ? ? 1 0 ? ? ? 0 0 0 1 0 1 1 1 1 1 0 ? ? 1 1 0 0 1 0 0 0 ? 1 0 0 0 1 0 ? 0 0 0 1 0 0 1 1 0 0 0 2 2 0 0 1 ? ? ? ? ? ? ? ? ? ? ? 1 ? ? ? ? ? ? ? ? ? ? ? ? ? ? ? ? ? ? ? ? ? ? ? ? ? ? ? ? ? ? ? ? ? ? ? ? ? ? ? ? ? 0 ? ? ? ? ? ? ? ? ? ? ? ? ? ? ? 0 ? ? 1 0 2 0 1 ? ? ? 0 0 0 2 1 ? 0 ? ? 0 ? ? ? ? ? ? ? ? ? ? ? ? ? ? ? ? ? ? ?

Ornithomimus 1 0 0 1 0 0 0 0 0 0 2 0 0 1 2 0 2 0 ? 1 1 0 0 1 0 0 1 2 0 1 1 0 0 0 0 0 0 0 0 1 0 0 0 0 0 1 1 1 1 1 0 1 0 1 0 0 0 0 1 0 1 10 0 1 1 1 0 0 0 0 0 0 0 0 0 ? 1 0 1 1 0 1 0 1 0 1 0 0 0 0 0 1 0 0 0 1 ? ? 0 0 ? 0 ? ? 0 0 0 1 0 0 0 0 0 0 0 1 0 0 0 1 0 1 0 0 0 0 0 ? ? 0 0 1 1 ? ? ? ? ? 1 ? ? ? ? ? 1 ? ? ? ? ? ? ? ? ? 0 1 0 1 ? ? 1 0 0 0 0 0 1 1 0 1 0 0 0 1 0 1 0 0 0 0 1 0 1 0 0 ? 0 0 0 1 0 0 0 0 ? 0 0 0 0 1 0 0 1 0 ? ? ? ? ? ? 3 0 0 1 1 1 1 0 2 1 1 0 0 1 1 2 0 0 0 0 1 1 0 0 1 0 1 0 0 0 0 ? ? 0 1 1 1 1 1 0 0 ? 1 1 0 0 1 0 0 0 0 1 0 0 0 1 1 1 0 0 0 2 0 0 1 1 0 0 0 2 2 0 0 1 0 0 1 0 0 0 0 0 0 0 1 1 0 0 0 0 0 0 1 0 0 0 0 0 0 1 0 0 0 0 0 0 ? 0 0 0 1 0 0 1 1 0 0 0 0 1 0 0 1 0 0 0 0 0 1 0 0 0 1 0 ? 0 0 0 1 0 1 0 0 0 1 1 1 0 2 0 1 ? ? ? 0 0 0 2 1 0 0 ? 0 0 ? ? 0 0 0 1 0 0 0 0 0 0 ? ? 0 0 0 0 1

Ornitholestes 2 0 0 0 0 0 0 0 0 0 0 0 1 1 0 0 1 0 ? 1 0 0 1 1 0 1 1 0 1 1 ? 0 0 ? 0 ? 0 0 0 0 0 0 0 0 0 1 1 0 1 1 0 1 0 1 0 0 0 0 0 0 1 00 1 1 ? 1 ? ? 0 0 ? 1 0 0 ? ? 1 ? ? 0 ? 0 0 ? 0 0 ? ? 1 0 1 ? 0 ? 0 ? ? ? 0 0 1 ? ? ? 0 0 0 0 0 0 0 0 0 0 0 1 0 0 0 0 0 0 0 0 0 0 0 ? 0 0 0 0 0 1 0 0 0 0 0 0 0 0 ? 0 0 0 0 0 1 ? 0 1 0 0 ? ? ? 1 0 1 1 0 0 0 ? 0 ? 0 0 1 1 0 0 1 0 1 0 0 0 0 ? ? 1 0 0 ? 0 0 0 ? ? 0 0 0 ? ? ? ? 1 1 ? ? ? ? ? ? ? ? ? ? ? ? ? ? ? ? ? ? ? ? ? ? ? 1 1 0 0 0 0 0 1 1 0 0 0 0 ? 0 0 ? ? ? ? ? 1 ? ? ? ? ? 0 ? ? ? ? 0 ? 0 ? ? 0 ? ? ? ? ? ? ? ? ? ? ? ? 0 0 0 0 0 0 1 0 0 0 ? 0 0 1 0 0 0 ? 0 0 0 0 1 0 ? 0 0 0 0 1 0 0 0 0 0 0 1 0 ? ? ? 0 0 1 0 0 1 0 0 0 0 1 0 0 0 0 1 0 1 ? 0 0 0 0 ? ? 0 0 ? ? 0 ? ? ? ? ? ? ? ? ? 0 1 0 1 0 1 0 ? ? ? ? 0 0 0 0 0 0 ? 0 0 0 ? ? ? ? ? ? ? ? ? 0 0 1 ? ? ? 0 0 0 0

Falcarius ? ? ? ? ? ? ? ? ? ? ? ? ? ? ? ? ? ? ? ? 1 ? ? ? 0 ? ? ? ? ? ? 0 ? ? 0 0 0 0 ? ? ? ? ? ? ? 1 1 ? ? ? ? ? ? ? ? ? ? ? ? ? ? ?1 ? ? 1 ? ? ? 0 0 ? ? ? ? 0 1 0 1 0 0 0 ? 0 ? 0 1 ? 1 0 0 0 2 0 0 0 1 ? ? ? ? ? ? ? ? ? 2 0 0 0 0 ? ? 0 0 1 ? ? ? ? ? ? ? ? ? ? ? ? ? ? ? ? ? 0 ? 0 ? ? ? 0 1 0 0 0 1 0 0 1 0 1 1 0 0 1 0 ? ? ? 1 1 0 0 0 0 ? 1 0 1 ? 0 1 1 0 0 1 0 1 1 0 0 0 0 ? 1 1 0 ? 0 0 1 ? ? 0 0 2 ? ? 0 0 0 1 ? ? ? ? ? ? ? ? 0 0 ? 1 ? 0 0 0 ? 0 1 0 0 ? 0 ? 1 0 1 0 1 0 ? 1 0 0 ? 1 1 1 0 1 m 1 0 0 1 0 0 ? 0 0 ? 1 0 0 0 0 0 0 1 0 0 1 0 0 0 1 0 0 0 0 0 0 0 0 0 0 0 0 0 0 0 0 0 0 1 0 1 0 0 0 1 0 0 1 0 1 0 1 0 0 0 2 1 0 0 0 0 0 0 1 0 0 0 0 ? 0 1 1 ? 0 2 0 0 0 1 1 0 ? ? 1 ? ? 1 0 0 1 1 0 1 0 0 0 0 0 0 0 0 1 0 0 1 0 0 0 ? 0 2 0 0 0 0 ? 0 0 0 0 0 0 0 0 0 0 0 0 ? ? 0 0 0 0 0 0 ? 0 0 1 0 ? 0 0 1 0 0

Beipiaosaurus 0 0 0 1 0 0 1 0 0 0 0 0 1 0 1 0 2 0 ? 1 ? 0 1 1 0 ? ? ? ? 1 ? 0 0 0 0 ? 0 0 0 0 0 0 0 0 0 1 1 0 ? 1 0 1 0 1 0 0 ? ? ? ? ? 0? ? 1 ? 1 ? ? ? ? 0 ? ? ? ? ? ? ? ? ? ? ? ? ? ? ? ? ? ? ? ? ? ? ? ? ? ? ? ? ? ? ? ? ? 0 ? 2 0 0 0 0 0 1 0 1 0 0 0 0 0 0 0 0 0 0 ? 0 ? ? 0 0 0 0 ? ? ? 0 0 0 1 0 0 0 1 0 0 0 0 0 ? 0 0 ? 0 0 1 0 1 1 0 ? 0 0 ? ? 0 0 0 0 1 ? 0 0 1 0 1 1 ? ? 0 ? ? ? ? ? 1 0 0 ? 0 ? ? ? ? ? ? ? ? ? ? 0 0 ? 0 ? ? ? ? 0 0 3 1 0 0 0 0 1 0 1 0 0 0 0 1 0 0 1 0 1 0 1 1 0 0 1 0 ? ? 0 ? 0 1 ? 0 1 0 0 0 0 0 0 1 0 0 1 0 0 0 1 0 0 1 ? 0 0 1 0 0 0 0 0 ? ? 0 0 0 0 0 0 0 0 ? ? 0 ? 0 1 0 1 ? ? 1 ? ? ? ? ? ? 1 0 0 ? ? ? ? ? ? 0 ? ? ? ? ? ? ? ? ? ? ? ? ? ? ? ? ? ? ? ? ? ? ? ? 1 ? 0 1 1 ? 0 ? 0 0 0 ? ? ? 0 1 0 0 ? ? 0 0 0 0 ? ? 0 0 0 ? 0 0 0 ? ? ? 0 ? ? ? ? ? ? 0 ? ? ? ? ? ? ? ? ? ? ? 1 ? ? ? ? ?

Martharaptor ? ? ? ? ? ? ? ? ? ? ? ? ? ? ? ? ? ? ? ? ? ? ? ? ? ? ? ? ? ? ? ? ? ? ? ? ? ? ? ? ? ? ? ? ? ? ? ? ? ? ? ? ? ? ? ? ? ? ? ? ? ?? ? ? ? ? ? ? ? ? ? ? ? ? ? ? ? ? ? ? ? ? ? ? ? ? ? ? ? ? ? ? ? ? ? ? ? ? ? ? ? ? ? ? ? ? ? ? ? ? ? ? ? ? ? ? ? ? ? ? ? ? ? ? ? ? ? ? ? ? ? ? ? ? ? ? ? ? ? ? ? ? ? ? ? ? ? ? ? ? ? ? ? ? ? ? ? ? ? 0 ? ? ? ? ? ? ? 0 0 ? 1 ? ? ? ? ? ? ? ? ? ? ? ? ? ? ? ? ? ? ? ? ? ? ? ? ? ? ? ? ? ? ? ? ? ? ? ? ? ? ? 3 1 0 0 0 0 ? ? ? ? ? ? ? ? ? ? ? ? ? ? ? ? ? ? ? ? ? ? ? ? ? ? ? ? ? ? ? ? 0 ? ? ? ? ? ? ? ? ? ? ? ? ? ? ? ? ? ? ? ? 0 ? ? ? 0 0 0 0 0 0 0 0 0 0 1 ? ? ? ? ? ? ? ? ? ? ? ? ? ? ? ? ? ? ? ? ? ? ? ? ? ? ? ? ? ? ? ? ? ? ? ? ? ? ? ? ? ? ? ? ? ? ? ? ? ? ? ? ? ? ? ? ? ? ? ? ? ? ? ? ? ? ? ? ? 0 ? ? ? ? ? 0 0 ? 0 0 0 0 ? ? ? ? ? ? ? 0 ? 0 ? ? ? ? ? ? ? ? ? 1 0 1 ? ? 1 0 0

Alxasaurus ? ? ? ? ? ? ? ? ? ? ? ? ? ? ? ? ? ? ? ? ? ? ? ? ? ? ? ? ? ? ? ? ? ? ? ? ? ? ? ? ? ? ? ? ? ? ? ? ? ? ? ? ? ? ? ? ? ? ? ? ? ?? ? ? ? ? ? ? ? ? ? ? ? ? ? ? ? ? ? ? ? ? ? ? ? ? ? ? ? ? ? ? ? ? ? ? ? ? ? ? ? ? ? ? 0 2 2 ? 0 0 ? ? 1 0 1 ? 0 ? ? ? ? ? ? ? ? ? ? ? ? ? ? ? ? ? ? ? ? ? ? 1 0 0 0 1 0 1 1 2 0 0 0 0 1 0 ? ? ? ? ? ? ? ? ? ? ? ? 1 1 0 1 ? 0 0 1 0 1 ? ? 0 0 0 ? 1 ? 0 ? 0 0 ? 0 ? 0 0 2 ? ? 0 0 ? 0 ? ? ? ? ? ? ? ? ? ? ? ? ? 0 ? ? ? ? ? 0 ? ? 0 1 0 0 1 0 ? 0 1 1 0 ? ? 0 ? ? 0 1 0 1 0 0 1 0 0 ? 0 0 ? 1 0 0 1 1 ? ? ? ? 0 ? ? ? ? ? ? ? 0 0 ? ? ? 0 0 0 0 1 1 0 0 0 0 ? 1 0 1 0 1 ? 1 1 0 1 0 1 0 ? 1 1 0 2 ? ? 2 ? ? ? ? ? ? 0 0 ? 0 ? ? 1 ? ? 0 0 0 ? 1 1 1 0 0 1 ? 1 ? ? 0 ? ? ? ? ? ? ? ? 0 0 ? 0 ? ? ? ? ? ? ? 0 ? ? ? ? 0 0 2 ? 1 ? 0 0 0 ? 0 0 ? 0 0 ? ? ? 0 0 0 0 0 0 ? 0 1 ? ? 0 ? 1 0 0

Nanshiungosaurus ? ? ? ? ? ? ? ? ? ? ? ? ? ? ? ? ? ? ? ? ? ? ? ? ? ? ? ? ? ? ? ? ? ? ? ? ? ? ? ? ? ? ? ? ? ? ? ? ? ? ? ? ? ? ? ? ? ? ? ? ? ?? ? ? ? ? ? ? ? ? ? ? ? ? ? ? ? ? ? ? ? ? ? ? ? ? ? ? ? ? ? ? ? ? ? ? ? ? ? ? ? ? ? ? ? ? ? ? ? ? ? ? ? ? ? ? ? ? ? ? ? ? ? ? ? ? ? ? ? ? ? ? ? ? ? ? ? ? ? ? ? ? ? ? ? ? ? ? ? ? ? ? ? ? ? ? ? ? ? ? ? ? ? ? ? 0 ? 1 0 1 1 0 0 1 ? 1 ? ? 0 ? 0 ? ? ? 0 ? ? ? ? ? ? ? ? ? ? ? ? ? ? ? ? ? ? ? ? ? ? ? ? ? ? ? ? ? ? ? ? ? ? ? ? ? ? ? ? ? ? ? ? ? ? ? ? ? ? ? ? ? ? ? ? ? ? ? ? ? ? ? ? ? ? ? ? ? ? ? ? ? ? ? ? ? ? ? ? ? ? ? ? ? ? ? ? ? ? ? ? ? ? ? ? ? ? ? 2 0 1 0 0 ? 2 ? 0 ? ? ? ? ? 1 1 ? ? ? 0 2 0 ? ? ? ? ? ? ? ? ? 0 ? ? 0 0 0 0 0 0 1 1 ? 0 1 1 ? 1 ? ? ? ? ? ? ? ? ? ? ? ? ? ? ? ? ? ? ? ? ? ? ? ? ? ? ? ? ? ? ? 1 ? ? ? ? ? ? ? ? ? ? ? ? ? ? ? ? ? ? ? ? ? ? ? ? ? ? ? ? ?

Suzhousaurus ? ? ? ? ? ? ? ? ? ? ? ? ? ? ? ? ? ? ? ? ? ? ? ? ? ? ? ? ? ? ? ? ? ? ? ? ? ? ? ? ? ? ? ? ? ? ? ? ? ? ? ? ? ? ? ? ? ? ? ? ? ?? ? ? ? ? ? ? ? ? ? ? ? ? ? ? ? ? ? ? ? ? ? ? ? ? ? ? ? ? ? ? ? ? ? ? ? ? ? ? ? ? ? ? ? ? ? ? ? ? ? ? ? ? ? ? ? ? ? ? ? ? ? ? ? ? ? ? ? ? ? ? ? ? ? ? ? ? ? ? ? ? ? ? ? ? ? ? ? ? ? ? ? ? ? ? ? ? ? ? ? ? ? ? ? ? ? ? 0 0 ? 0 0 1 0 1 1 1 0 1 0 0 0 0 0 ? 0 0 1 ? ? 0 ? ? ? ? ? 0 0 ? ? ? ? ? ? ? ? ? ? ? 3 ? ? 0 0 ? ? 0 1 0 ? 0 0 1 0 0 1 0 1 0 ? ? ? ? ? ? ? ? ? ? ? ? ? ? ? ? ? ? ? ? ? ? ? ? ? ? ? ? ? ? ? ? ? ? ? ? ? ? ? ? ? ? ? ? ? ? ? ? ? ? ? ? ? ? 2 0 1 0 1 0 2 1 0 1 0 1 0 1 1 1 0 2 1 0 2 0 1 0 0 1 0 ? ? ? 0 0 0 1 0 0 0 0 1 0 1 1 1 0 0 1 1 ? ? ? 0 1 1 ? ? 0 ? 0 0 0 ? ? ? ? ? ? ? ? ? ? ? ? ? ? ? ? ? ? ? ? ? ? ? ? ? ? ? ? ? ? ? ? ? ? ? ? ? ? ? ? ? ? ? ? ? ? ? ? ?

Nothronychus ? ? ? ? ? ? ? ? ? ? ? ? ? ? ? ? ? ? ? ? ? ? ? ? ? ? ? ? ? ? ? ? ? ? ? ? ? ? ? ? ? ? ? ? ? ? ? ? ? ? ? ? ? ? ? ? ? ? ? ? ? ?? ? ? ? ? ? ? ? ? ? ? ? ? 0 1 ? ? 0 ? ? ? ? ? ? ? ? 0 1 0 0 2 ? 0 0 0 ? ? ? ? ? ? ? ? ? ? ? ? ? ? ? ? ? ? ? ? ? ? ? ? ? ? ? ? ? ? ? ? ? ? ? ? ? ? ? ? ? ? 0 1 0 0 0 1 0 ? ? ? 0 0 0 0 1 0 ? ? ? 1 ? 0 0 0 0 0 1 0 ? 1 0 1 1 0 ? 0 0 ? 1 1 0 0 1 ? 1 ? 0 1 ? 0 1 0 ? 0 ? 2 ? 0 0 0 ? ? ? ? ? ? ? ? ? ? 0 0 ? ? ? 0 ? 0 ? ? 1 0 ? ? ? 1 0 0 1 0 1 0 1 1 0 ? ? 0 1 0 0 ? ? ? ? 0 1 0 ? 0 0 ? 0 ? 0 ? ? ? 0 0 ? 0 0 ? 0 0 ? ? 0 0 ? 0 ? ? ? 0 0 0 0 0 0 0 0 0 0 ? 2 ? 1 0 1 ? ? 1 0 ? 0 ? ? ? 1 ? 0 ? ? 0 2 0 1 0 0 ? ? 1 0 0 0 0 1 1 1 ? 0 0 1 0 1 1 1 0 0 1 1 1 0 ? ? 1 1 ? ? ? 0 0 ? ? ? ? 0 1 0 ? ? ? 0 0 0 ? 2 0 0 0 0 2 1 1 0 0 0 0 0 0 1 ? ? 0 ? ? 0 ? ? ? ? ? ? 0 1 1 ? ? ? ? 1 0 0

Segnosaurus 2 ? ? ? ? ? ? ? ? ? ? ? ? ? ? ? ? ? ? ? ? ? ? ? ? ? ? ? ? ? ? ? ? ? ? ? ? ? ? ? ? ? ? ? ? ? ? ? ? ? ? ? ? ? ? ? ? ? ? ? ? ?? ? ? ? ? ? ? ? ? ? ? ? ? ? ? ? ? ? ? ? ? ? ? ? ? ? ? ? ? ? ? ? ? ? ? ? ? ? ? ? ? ? ? 0 ? 2 ? 0 0 ? ? 1 ? 1 0 ? ? 0 0 ? 0 0 0 0 0 0 ? 0 0 0 0 ? ? ? ? ? ? ? 1 0 0 0 1 0 1 1 2 0 ? 0 0 1 0 ? ? ? ? ? ? ? ? ? ? ? ? ? ? ? ? ? ? ? ? ? ? ? ? ? ? ? ? ? ? 0 ? ? ? ? ? ? ? ? ? ? ? ? ? ? ? ? ? ? ? ? ? ? ? ? ? ? ? 2 ? 1 1 1 ? 1 ? ? 0 ? ? 0 0 1 0 1 0 ? 1 0 ? ? ? ? ? ? ? ? ? ? ? ? ? ? ? ? ? ? ? ? ? ? ? ? ? ? ? ? ? ? ? ? ? ? ? ? 0 ? ? ? ? ? ? ? ? ? ? ? ? ? ? 2 0 1 0 1 ? 2 1 0 1 0 1 0 1 1 1 0 2 1 0 2 0 1 0 ? ? ? 0 0 0 0 0 0 ? ? 0 0 0 1 ? 1 1 1 0 0 1 ? 1 0 ? ? ? ? ? ? ? ? ? ? 0 ? ? 0 ? ? 0 ? 0 ? ? 0 ? 2 0 ? 0 0 2 1 1 0 0 0 0 0 0 1 ? 0 0 1 0 ? 0 0 ? ? ? ? ? 1 1 1 1 ? ? 1 0 0

Erliansaurus ? ? ? ? ? ? ? ? ? ? ? ? ? ? ? ? ? ? ? ? ? ? ? ? ? ? ? ? ? ? ? ? ? ? ? ? ? ? ? ? ? ? ? ? ? ? ? ? ? ? ? ? ? ? ? ? ? ? ? ? ? ?? ? ? ? ? ? ? ? ? ? ? ? ? ? ? ? ? ? ? ? ? ? ? ? ? ? ? ? ? ? ? ? ? ? ? ? ? ? ? ? ? ? ? ? ? ? ? ? ? ? ? ? ? ? ? ? ? ? ? 0 ? ? ? ? ? ? ? ? ? ? ? ? ? ? ? ? ? ? ? ? ? ? ? ? ? ? ? ? ? ? ? ? ? ? ? ? ? ? ? ? ? ? ? ? ? ? ? ? ? 1 0 ? ? ? ? ? ? ? ? ? ? ? ? ? ? ? ? ? ? ? ? ? ? ? ? ? ? ? ? ? ? ? ? ? ? ? ? ? ? ? ? ? ? ? ? ? ? ? ? ? ? 0 1 0 0 1 0 1 1 1 1 0 1 ? 0 1 ? 0 ? ? ? ? 0 1 0 0 0 0 1 0 1 0 0 1 1 0 0 1 0 ? 0 0 1 0 0 0 0 0 0 0 1 0 0 0 0 1 1 1 0 0 0 0 1 ? ? ? ? ? ? ? ? 0 ? ? ? ? ? 1 ? ? ? 1 ? ? ? ? ? ? ? ? ? ? ? ? ? ? ? ? ? ? ? ? ? ? ? ? ? ? ? ? ? 0 0 ? 1 ? ? ? 0 0 ? 0 0 ? 0 0 ? 0 ? ? 0 ? ? ? ? ? ? ? ? ? ? ? ? ? ? ? ? ? ? ? ? ? ? ? ? ? ? ? ? ? ? ? ? ? ? ? ? ? ? ? ? ?

Neimongosaurus ? ? ? ? ? ? ? ? ? ? ? ? ? ? ? ? ? ? ? ? ? ? ? ? ? ? ? ? ? ? ? ? ? ? ? ? ? ? ? ? ? ? ? ? ? ? ? ? ? ? ? ? ? ? ? ? ? ? ? ? ? ?? ? ? ? ? ? ? ? ? ? ? ? ? ? ? ? ? ? ? ? ? ? ? ? ? ? ? ? ? ? ? ? ? ? ? ? ? ? ? ? ? ? ? ? 2 1 0 0 0 ? ? ? 0 ? ? ? ? ? ? ? ? ? ? ? ? ? ? ? ? ? ? ? ? ? ? ? ? ? 1 ? 0 0 ? 0 1 ? ? 0 ? 0 0 1 0 1 ? ? 1 1 0 1 0 0 0 ? 0 1 1 0 1 1 0 0 1 0 1 1 ? ? 0 1 ? ? 2 ? 1 0 0 ? 0 ? 0 0 2 ? 0 0 0 ? 0 ? ? ? ? ? ? ? ? 0 0 ? ? ? 0 0 1 ? 0 1 0 0 0 0 1 0 0 1 0 1 1 1 1 ? 1 ? ? ? ? ? ? ? ? ? ? ? ? ? ? ? ? ? ? ? ? ? ? ? ? ? ? ? ? ? ? ? ? ? ? ? ? ? ? ? ? ? ? ? ? ? ? ? ? ? ? 2 ? 1 0 ? ? 2 ? 0 1 0 1 0 1 1 1 0 2 ? ? ? ? ? ? ? ? ? ? ? ? ? ? ? ? ? ? ? ? ? ? ? ? ? ? ? ? ? ? 0 0 0 1 ? ? 1 0 ? 0 0 0 0 0 ? ? ? ? ? ? 0 0 0 0 2 0 0 0 0 2 1 1 0 0 0 0 0 0 1 ? ? 0 ? 0 0 0 0 0 0 0 0 0 1 ? ? ? ? ? ? ? ?

Erlikosaurus ? 0 0 0 0 0 0 0 0 0 0 0 1 0 1 0 2 0 0 1 1 0 1 1 0 ? 0 ? ? 1 1 0 0 0 0 0 0 0 0 0 0 0 0 0 0 1 1 0 1 1 0 1 0 1 0 0 1 0 0 0 1 0? ? 1 1 1 1 0 0 0 0 1 1 0 ? ? ? ? 0 2 ? 1 0 ? 1 ? 0 0 1 0 0 ? 0 0 0 0 1 1 1 0 ? ? 1 1 0 2 2 0 0 0 0 0 1 0 1 0 0 0 0 0 0 0 0 0 0 0 0 2 0 0 0 0 1 ? ? ? ? 0 0 1 0 0 0 1 0 1 1 2 0 0 0 0 1 0 ? ? ? ? ? ? ? ? ? ? ? ? ? ? ? ? ? ? ? ? ? ? ? ? ? ? ? ? ? ? ? ? ? ? ? ? ? ? ? ? ? ? ? ? ? ? ? ? ? ? ? ? ? ? ? ? ? ? ? ? ? ? ? ? ? ? ? ? ? ? 0 0 1 1 1 1 ? ? ? ? ? ? ? ? ? ? ? ? ? ? ? ? ? ? ? ? ? ? ? ? ? ? ? ? ? ? ? ? ? ? ? ? ? ? ? ? ? ? ? ? ? ? ? ? ? ? ? ? ? ? ? ? ? ? ? ? ? ? ? ? ? ? ? ? ? ? ? ? ? ? ? ? ? ? ? ? ? ? ? ? ? ? ? ? ? ? ? ? ? ? ? ? ? ? ? ? ? ? ? ? ? ? ? ? ? ? ? ? ? ? ? ? ? ? ? ? ? ? ? 0 0 ? 2 0 0 ? 0 2 1 1 0 0 0 ? ? ? ? ? ? ? ? 0 0 0 0 0 0 0 0 0 1 1 1 1 0 0 1 0 0

Therizinosaurus ? ? ? ? ? ? ? ? ? ? ? ? ? ? ? ? ? ? ? ? ? ? ? ? ? ? ? ? ? ? ? ? ? ? ? ? ? ? ? ? ? ? ? ? ? ? ? ? ? ? ? ? ? ? ? ? ? ? ? ? ? ?? ? ? ? ? ? ? ? ? ? ? ? ? ? ? ? ? ? ? ? ? ? ? ? ? ? ? ? ? ? ? ? ? ? ? ? ? ? ? ? ? ? ? ? ? ? ? ? ? ? ? ? ? ? ? ? ? ? ? ? ? ? ? ? ? ? ? ? ? ? ? ? ? ? ? ? ? ? ? ? ? ? ? ? ? ? ? ? ? ? ? ? ? ? ? ? ? ? ? ? ? ? ? ? ? ? ? ? ? ? ? ? ? ? ? ? ? ? ? ? ? ? ? ? ? ? ? ? ? ? ? ? ? ? ? ? ? ? ? ? ? ? ? ? ? ? ? ? ? ? ? 2 0 0 1 ? 0 1 0 0 0 ? ? 0 0 1 1 1 1 ? 0 ? 1 ? 0 ? ? 0 ? 0 1 1 0 1 0 0 0 0 1 ? 1 0 0 1 1 ? ? ? ? ? 0 0 1 ? ? 0 ? ? 0 ? ? ? 0 0 0 ? ? 1 ? 1 0 0 ? ? ? ? ? ? ? ? ? ? ? ? ? ? ? ? ? ? ? ? ? ? ? ? ? ? ? ? ? ? ? ? ? ? ? ? ? ? ? ? ? ? ? ? ? ? ? ? ? ? ? ? ? ? ? ? ? ? ? ? ? ? ? ? ? ? ? ? ? ? ? ? ? ? ? ? ? ? ? ? ? ? ? ? ? ? ? ? ? ? ? ? ? ? ? ? ? ? ? ? ? ? ? ? ? ? ? ? ? ?

Haplocheirus 0 0 0 0 0 0 0 0 0 0 0 0 1 1 1 0 0 ? ? 1 0 0 1 1 0 1 1 0 1 0 1 0 0 0 0 0 0 0 ? ? 0 0 0 0 0 1 1 0 ? 1 0 1 0 1 0 0 0 0 0 0 1 0? ? 1 ? ? ? ? 0 0 0 ? ? 0 ? ? ? ? ? ? ? 0 1 1 0 ? ? 0 1 ? 0 ? ? ? 0 ? 0 1 0 ? ? 0 1 0 0 0 0 0 0 0 1 ? 0 0 0 1 0 ? 0 1 ? 1 0 0 0 1 0 ? 1 0 ? 0 0 1 0 ? 0 ? 0 3 0 0 1 2 0 0 1 ? 1 ? 0 0 0 0 0 ? ? 1 1 ? ? 0 0 ? ? ? 1 0 1 2 1 0 1 1 0 1 1 1 0 ? 0 ? 0 0 0 ? ? 0 0 0 ? 0 ? 2 ? 0 0 0 ? ? 0 ? ? ? ? ? ? ? ? ? 0 ? 0 ? 0 0 ? 0 2 0 0 0 0 0 0 3 0 1 0 0 1 0 ? 0 0 0 2 0 0 ? ? 1 ? 0 1 0 ? 0 0 ? 0 1 0 0 1 0 0 0 1 0 ? 1 0 0 0 1 0 0 0 0 0 1 0 0 1 1 1 0 0 0 0 0 0 0 ? ? ? ? ? ? ? ? 0 0 0 1 ? ? ? 0 0 0 1 0 0 0 0 0 0 1 0 0 0 1 0 0 ? 0 0 0 0 0 0 0 2 ? 0 0 0 1 1 1 1 ? 0 1 0 1 0 0 0 0 0 0 ? 0 0 ? ? ? ? ? 0 0 1 ? 1 0 ? 0 ? ? ? ? 0 0 0 0 0 0 0 ? 0 0 1 ? 0 0 0 0 0 0 0 0 0 ? ? ? 0 0 1 0 0

Alvarezsaurus ? ? ? ? ? ? ? ? ? ? ? ? ? ? ? ? ? ? ? ? ? ? ? ? ? ? ? ? ? ? ? ? ? ? ? ? ? ? ? ? ? ? ? ? ? ? ? ? ? ? ? ? ? ? ? ? ? ? ? ? ? ?? ? ? ? ? ? ? ? ? ? ? ? ? ? ? ? ? ? ? ? ? ? ? ? ? ? ? ? ? ? ? ? ? ? ? ? ? ? ? ? ? ? ? ? ? ? ? ? ? ? ? ? ? ? ? ? ? ? ? ? ? ? ? ? ? ? ? ? ? ? ? ? ? ? ? ? ? ? ? ? ? ? ? ? ? ? ? ? ? ? ? ? ? ? ? ? 1 1 1 ? ? ? ? 0 0 ? 0 0 2 ? ? ? 1 ? ? 1 ? ? ? ? ? 2 ? 1 ? 0 1 2 ? ? 0 0 2 ? 0 0 ? ? 1 ? ? ? ? ? ? ? ? ? ? ? 0 0 ? ? 0 ? 0 ? 0 ? ? ? ? ? ? ? ? ? ? ? ? ? ? ? ? ? ? ? ? ? ? ? ? ? ? ? ? ? ? ? ? ? ? ? ? ? ? ? ? ? ? ? ? ? ? ? ? ? ? ? ? ? ? 1 1 ? 0 ? 1 ? ? ? ? 0 0 ? ? 0 ? 2 0 2 1 0 1 0 1 0 2 0 1 ? ? ? ? ? ? ? ? ? ? ? ? ? ? ? ? ? ? ? ? ? ? ? ? ? ? ? ? ? ? ? ? 1 ? 0 ? ? 0 ? 0 ? ? ? ? 0 ? 0 ? 1 1 0 0 ? ? 1 ? 1 0 ? ? ? ? 0 0 0 0 0 0 ? ? ? 0 ? ? 0 ? ? 0 0 0 0 0 0 0 ? ? 0 ? 0 0 0

Patagonykus ? ? ? ? ? ? ? ? ? ? ? ? ? ? ? ? ? ? ? ? ? ? ? ? ? ? ? ? ? ? ? ? ? ? ? ? ? ? ? ? ? ? ? ? ? ? ? ? ? ? ? ? ? ? ? ? ? ? ? ? ? ?? ? ? ? ? ? ? ? ? ? ? ? ? ? ? ? ? ? ? ? ? ? ? ? ? ? ? ? ? ? ? ? ? ? ? ? ? ? ? ? ? ? ? ? ? ? ? ? ? ? ? ? ? ? ? ? ? ? ? ? ? ? ? ? ? ? ? ? ? ? ? ? ? ? ? ? ? ? ? ? ? ? ? ? ? ? ? ? ? ? ? ? ? ? ? ? ? ? ? ? ? ? ? ? ? ? ? ? ? ? ? 1 ? 0 1 1 1 ? ? ? ? ? ? 1 ? 0 1 2 ? ? ? ? ? ? ? ? ? ? ? ? ? ? ? ? ? ? ? ? ? ? ? ? ? ? 0 ? 0 2 0 0 ? ? ? ? 3 0 1 2 ? ? 0 0 0 ? ? 2 0 1 ? 1 1 0 1 1 ? 0 ? 0 ? ? ? ? ? ? 0 ? 0 ? ? 1 ? ? ? ? ? ? ? ? ? ? ? ? 0 1 1 1 0 ? 1 ? 0 ? ? ? ? ? ? ? ? ? ? ? ? ? 1 ? ? ? ? ? ? 1 ? 1 0 0 0 0 1 0 0 0 0 ? 0 0 ? ? ? ? ? ? ? ? ? ? ? ? ? ? ? ? ? 1 1 0 ? ? ? ? 1 0 0 1 0 ? ? ? 0 1 1 1 ? ? ? 1 ? ? 0 ? ? ? ? ? ? ? 0 0 0 ? ? ? ? ? ? ? ? ? ? ? ? ? ? ? 0 ? 0 ? ? ? ? ?

Parvicursor 0 ? ? ? ? ? ? ? ? ? ? ? ? ? ? ? ? ? ? ? ? ? ? ? ? ? ? ? ? ? 1 ? ? ? ? ? ? ? ? ? ? ? ? ? ? ? ? ? ? ? ? ? ? ? ? ? ? ? ? ? ? ?? ? ? ? ? ? ? ? ? ? ? ? ? ? ? ? ? ? ? ? ? ? ? ? ? ? ? ? ? ? ? ? ? ? ? ? ? ? ? ? ? 1 ? 0 0 0 ? 0 0 ? ? 0 0 0 ? ? ? ? ? ? ? ? ? ? ? 0 ? ? ? ? ? ? ? ? ? ? ? ? ? ? ? ? ? ? ? ? ? ? ? ? ? ? ? 0 ? ? 1 1 ? 1 0 0 ? ? 0 1 0 ? 2 ? 0 ? 1 0 1 1 1 0 ? 1 ? 2 ? 1 0 0 1 2 0 ? 0 0 2 ? ? ? 0 0 ? ? ? ? ? ? ? ? ? ? ? ? ? ? ? ? ? ? ? ? ? ? ? ? ? ? ? ? ? ? ? ? ? ? ? ? ? ? ? ? ? ? ? ? ? ? ? ? ? ? ? 0 ? ? ? ? 0 ? 0 ? ? ? 0 0 ? 0 ? 0 1 0 0 ? ? ? 0 1 1 1 0 0 1 1 0 0 1 ? ? 0 0 ? ? 2 0 2 1 0 1 0 1 0 2 0 1 1 0 3 0 0 0 1 ? ? ? ? 2 0 1 0 0 0 0 0 0 0 0 2 ? ? ? ? ? 2 1 1 ? 1 1 1 1 0 0 ? 1 0 0 1 0 1 1 ? 2 1 1 1 0 1 1 1 0 2 0 0 1 0 0 0 0 0 2 1 1 0 ? 0 0 ? 0 0 0 0 0 0 0 0 0 0 ? 0 0 0 0 0 0 0

Mononykus ? ? ? ? ? ? ? ? ? ? ? ? ? ? ? 0 ? ? ? ? ? ? ? ? ? ? ? ? ? ? ? ? ? ? ? ? ? ? ? ? ? ? ? ? ? ? ? ? ? ? ? ? ? ? ? ? ? ? ? ? ? ?? ? ? ? ? ? ? ? ? ? ? ? ? ? 0 1 1 0 ? ? ? ? ? ? ? ? ? 1 0 0 2 ? ? ? ? ? ? ? ? ? ? ? ? ? ? ? ? ? ? ? ? ? ? ? ? ? ? ? ? ? ? ? ? ? ? ? ? ? ? ? ? ? ? ? ? ? ? ? 0 ? 0 2 ? ? ? 0 ? ? ? ? 0 ? ? ? ? ? 1 ? ? ? 0 0 1 ? 0 1 0 1 2 1 0 1 1 0 0 1 1 0 1 ? ? ? ? 1 ? 0 ? 2 ? ? ? ? ? ? ? ? ? ? ? ? ? ? 1 1 0 0 0 ? ? 0 0 0 0 0 0 ? 0 2 0 0 0 0 0 0 3 0 1 2 1 0 0 0 0 0 0 2 0 1 ? 1 1 2 1 1 2 0 1 0 0 ? 1 1 0 ? 0 ? 0 0 0 1 ? ? ? ? ? ? ? ? ? ? ? ? 0 1 1 1 0 ? 1 ? 0 ? ? 0 ? 0 0 ? ? ? ? 2 ? ? ? 0 ? 0 2 ? 1 1 ? 3 0 0 ? 1 ? ? ? ? ? ? ? ? 0 ? ? 0 0 ? ? ? ? ? ? ? ? ? ? 1 0 1 1 1 1 0 0 0 1 0 1 1 1 1 1 0 2 1 1 1 0 ? ? 1 ? ? 0 ? ? 0 0 0 0 0 2 1 1 0 ? 0 0 ? 0 0 0 0 0 0 0 0 0 0 0 0 0 0 0 0 0 0

Shuvuuia 0 0 0 1 0 0 0 0 0 0 1 0 0 1 1 0 1 0 0 1 0 0 1 1 0 ? 1 0 0 1 ? 0 0 0 0 0 0 0 0 0 0 0 0 0 0 1 1 0 ? 1 0 1 1 1 0 1 ? 0 0 0 1 00 1 1 ? 1 1 ? 0 0 0 0 1 0 1 0 1 1 0 0 0 0 1 1 0 0 ? 0 1 0 ? 2 1 0 0 0 1 ? ? 0 ? ? 1 0 0 0 0 0 0 0 1 0 0 0 0 1 0 0 0 0 ? 0 0 0 0 0 0 2 1 0 0 0 ? ? 0 ? 0 ? 0 1 0 0 2 2 0 0 0 0 ? ? ? 0 1 0 ? 1 0 1 1 1 1 0 0 1 0 0 1 0 1 ? 1 ? 1 ? ? 0 ? 1 0 ? 1 ? 2 0 1 0 0 1 2 0 ? 0 0 2 ? ? 0 0 0 1 0 0 ? 1 1 0 ? 0 ? ? 0 0 0 0 0 0 1 0 2 0 0 0 0 0 0 3 0 1 2 1 0 0 0 0 0 0 2 ? 1 ? 1 1 2 1 1 2 ? 1 0 0 ? ? 1 ? ? 0 ? 0 0 0 1 ? ? ? ? ? ? ? ? ? ? ? ? 0 1 1 1 0 0 1 ? 0 ? ? ? ? 0 0 ? ? 2 0 ? 1 0 1 0 1 0 2 0 1 1 0 3 0 0 0 1 ? ? ? ? 2 0 1 0 0 0 0 0 0 0 0 2 ? ? ? ? ? 2 1 1 ? 1 1 1 1 0 0 0 1 0 1 ? 1 1 1 0 2 1 1 1 0 1 1 1 0 2 0 0 1 0 0 0 0 0 2 1 1 0 ? 0 0 1 0 0 0 0 0 0 0 0 0 0 0 0 0 0 0 0 0 0

Incisivosaurus ? 0 0 0 0 0 0 0 0 0 0 0 1 1 0 0 1 0 ? 1 0 0 1 0 0 0 1 0 0 1 1 0 1 0 0 ? 0 0 0 0 1 0 2 0 0 1 1 0 1 1 0 1 0 1 0 0 0 0 0 0 0 01 0 1 1 1 ? 0 0 0 0 1 1 ? ? ? ? ? ? 1 ? ? 0 0 1 ? ? 1 1 1 0 ? 0 1 0 0 1 1 1 1 1 0 1 1 0 2 0 0 0 0 1 1 0 0 0 1 0 0 0 0 0 0 0 0 0 1 0 1 ? ? 1 0 0 1 3 0 1 ? 0 1 0 0 2 1 0 1 0 0 ? ? ? 0 0 0 ? ? ? ? ? ? ? ? ? ? ? ? ? ? ? ? ? ? ? ? ? ? ? ? ? ? ? ? ? ? ? ? ? ? ? ? ? ? ? ? ? ? ? ? ? ? ? ? ? ? ? ? ? ? ? ? ? ? ? ? ? ? ? ? ? ? ? ? ? ? ? ? ? ? ? ? ? ? ? ? ? ? ? ? ? ? ? ? ? ? ? ? ? ? ? ? ? ? ? ? ? ? ? ? ? ? ? ? ? ? ? ? ? ? ? ? ? ? ? ? ? ? ? ? ? ? ? ? ? ? 0 ? ? ? ? ? ? ? ? ? ? ? ? ? ? ? ? ? ? ? ? ? ? ? ? ? ? ? ? ? ? ? ? ? ? ? ? ? ? ? ? ? ? ? ? ? ? ? ? ? ? ? ? ? ? ? ? ? ? ? ? ? ? ? ? ? ? ? ? ? ? ? ? ? ? ? ? ? ? ? ? ? ? ? ? ? ? ? ? ? ? ? ? ? ? ? ? ? ? ? ? ? ? ? ? ? ? ? ?

Protarchaeopteryx 1 ? 0 ? 0 ? 0 0 0 0 0 ? ? ? ? ? ? ? ? ? ? 0 1 0 0 ? ? ? ? ? ? ? ? ? ? ? ? ? ? ? ? ? ? ? ? ? ? ? ? 1 0 1 0 1 ? ? ? ? ? ? ? ?? ? ? ? ? ? ? ? ? ? ? ? ? ? ? ? ? ? ? ? ? ? ? ? ? ? ? ? ? ? ? ? ? ? ? ? ? ? ? ? ? ? ? 0 ? 0 0 0 ? ? ? 0 0 0 1 ? ? ? ? ? ? ? ? 0 ? ? ? ? ? ? 0 0 1 3 0 1 ? 0 1 ? 0 2 1 0 1 0 0 ? ? ? 0 0 0 ? ? ? 1 ? ? ? 0 0 0 ? ? ? 0 ? 1 ? ? ? 1 0 ? ? ? ? ? ? ? ? ? ? 1 ? 0 ? 0 ? 0 ? 2 ? 0 0 0 ? 1 ? ? ? 1 0 ? ? ? 0 0 0 ? 0 ? ? ? ? ? 1 ? ? 0 ? 1 1 ? 0 0 ? 0 1 1 0 0 1 1 0 ? 0 ? 1 1 1 0 1 0 ? 0 ? 0 0 ? ? ? 0 0 0 0 1 0 0 1 ? 0 0 1 0 0 0 0 0 0 0 0 0 0 0 0 0 0 0 0 1 0 ? 2 0 0 0 ? ? 0 0 1 ? ? 0 ? 0 ? ? ? ? 0 ? ? 0 0 0 ? 0 0 0 ? 1 0 ? ? ? 0 2 ? 0 ? 1 1 0 0 0 1 ? 1 1 ? 1 1 ? ? 0 ? 0 ? ? ? ? ? ? ? ? ? ? ? ? ? 1 ? 1 0 1 0 0 0 0 0 0 ? ? ? ? 0 ? 1 0 0 ? 0 0 ? 0 0 0 0 ? 0 ? ? 0 0 0 0 0 0 0

Caudipteryx 1 0 0 0 0 0 0 0 0 1 0 0 1 1 0 0 1 0 ? 1 0 0 1 1 0 0 1 0 0 1 ? 0 0 0 0 0 0 0 0 0 1 3 2 0 0 1 1 0 1 1 0 1 0 1 0 1 0 0 0 ? ? 0? ? 1 1 1 1 0 0 0 1 1 ? ? ? ? ? ? ? ? ? 0 ? ? ? ? ? ? ? ? ? ? ? ? ? ? ? ? ? ? ? ? ? ? 0 2 1 0 0 0 1 1 0 0 0 1 0 0 0 0 0 0 0 0 1 ? ? ? ? 0 1 0 0 1 3 0 0 ? 1 ? ? ? ? ? 1 ? ? ? ? ? ? 0 ? ? 0 ? 0 1 1 0 ? 0 0 0 0 0 1 0 0 1 1 0 0 1 0 1 1 1 1 0 0 ? ? ? 0 1 0 0 ? 0 ? 0 0 2 ? 0 0 0 0 1 1 1 0 1 0 0 ? ? ? 0 0 1 3 0 0 0 ? 0 1 0 1 0 0 0 1 0 0 0 0 0 1 1 0 0 1 1 0 ? 0 ? 1 1 0 0 1 0 0 0 0 0 0 ? 0 0 0 0 0 0 1 0 0 1 0 0 0 0 0 ? ? 0 1 ? ? 0 0 0 0 0 0 1 1 0 0 ? 0 0 1 0 0 1 ? 0 0 0 0 1 0 ? 0 0 0 2 1 0 1 0 1 0 0 1 0 0 0 0 1 0 0 1 0 0 2 0 0 0 1 1 0 0 0 1 1 1 1 ? 1 1 1 1 0 0 1 1 0 0 ? ? 0 ? ? 0 1 ? 0 0 1 0 1 0 1 0 0 0 0 0 0 0 0 2 0 0 0 1 0 0 1 0 0 0 0 0 0 0 0 0 0 1 0 0 0 0 0 0 0

Microvenator ? ? ? ? ? ? ? ? ? ? ? ? ? ? ? ? ? ? ? ? ? ? ? ? ? ? ? ? ? ? ? ? ? ? ? ? ? ? ? ? ? ? ? ? ? ? ? ? ? ? ? ? ? ? ? ? ? ? ? ? ? ?? ? ? ? ? ? ? ? ? ? ? ? ? ? ? ? ? ? ? ? ? ? ? ? ? ? ? ? ? ? ? ? ? ? ? ? ? ? ? ? ? ? ? ? 2 1 0 0 0 1 ? 0 0 ? ? ? ? ? ? ? ? ? ? 1 ? ? ? ? ? ? ? ? ? ? ? ? ? ? ? ? ? ? ? 1 ? ? ? ? ? ? ? ? ? ? 1 0 1 1 0 ? ? ? ? ? 0 ? 0 ? 1 ? 1 ? 1 0 1 1 1 1 0 ? ? 1 ? ? ? ? 0 1 ? ? ? 0 2 ? ? ? ? ? ? ? ? ? ? ? ? ? ? ? ? ? ? ? ? ? 0 ? ? 1 0 1 0 ? 1 1 0 0 0 0 0 1 1 0 0 ? 1 0 1 0 ? ? ? ? 0 1 ? 0 ? 0 ? ? ? ? ? ? 0 ? ? ? ? ? ? ? ? ? ? ? ? ? 0 ? ? ? 0 0 0 ? ? 0 ? 0 ? 1 ? 0 0 1 0 0 1 1 0 0 ? 0 1 0 1 0 0 0 2 1 0 1 0 1 0 0 1 0 0 0 0 ? ? ? ? ? ? ? ? ? ? ? ? ? ? ? ? ? ? 1 0 0 1 1 1 0 0 1 1 0 0 ? 0 ? 0 1 0 1 1 0 ? ? ? ? ? ? ? 0 ? 0 ? ? ? ? ? ? ? ? ? ? ? ? ? ? ? ? ? ? ? ? ? ? ? ? ? ? ? ? ? ?

Avimimus ? ? ? ? ? 0 0 0 ? 1 ? ? ? ? ? ? ? ? ? ? ? ? ? ? ? ? ? ? ? ? ? ? 1 ? 0 1 0 0 ? ? ? ? ? ? ? 1 1 0 ? ? ? 1 ? ? ? 1 ? ? 0 0 0 00 1 0 1 1 ? ? 0 0 ? 1 1 0 ? ? 0 ? ? 1 0 0 0 0 1 ? 0 0 1 1 0 ? 1 0 0 0 ? ? ? ? ? ? ? ? 0 2 ? ? ? ? ? ? ? 0 ? 1 1 0 0 0 0 ? 0 0 1 ? ? ? 0 1 1 0 0 ? ? ? ? ? ? ? ? ? ? ? 1 ? ? ? ? ? ? ? ? ? ? 1 0 1 1 0 1 0 0 1 1 0 ? 0 0 1 1 0 0 ? 0 1 1 1 ? 0 ? ? 1 0 0 ? ? ? ? ? ? ? ? ? ? ? ? ? ? ? ? ? ? ? ? ? ? ? ? ? ? ? ? 0 0 1 ? 0 ? 0 1 ? 0 1 1 0 0 0 0 0 ? ? ? ? ? 1 0 ? ? ? 1 2 0 1 1 ? ? ? ? ? ? ? 0 ? ? ? ? ? ? ? ? ? ? ? ? ? ? ? ? ? ? ? ? ? ? ? ? ? ? ? ? ? ? ? 0 ? 0 0 ? ? ? 0 0 1 0 1 0 1 ? ? 0 2 1 0 1 0 0 0 0 1 0 0 0 0 ? 0 ? 1 0 0 ? 0 0 0 1 1 0 ? ? ? ? ? 1 0 0 1 1 1 0 0 0 0 0 0 ? 0 ? ? 1 0 1 1 1 1 1 0 1 0 2 1 ? ? ? ? 0 0 0 2 1 0 ? 1 0 0 ? ? 0 0 0 0 1 0 0 0 0 1 ? ? 0 0 0 0 0

Oviraptor ? 0 0 0 0 ? ? ? ? ? ? 1 ? ? 0 0 2 0 ? 1 0 0 1 1 0 ? 1 0 0 1 ? 1 ? ? ? ? 0 0 0 0 1 3 2 0 0 1 1 0 1 1 0 1 1 1 1 1 ? 0 ? ? 0 0? 1 0 ? 1 ? ? 0 ? ? 1 ? ? ? ? 1 ? ? ? ? ? 0 ? ? ? ? 0 ? ? ? ? 1 0 0 0 1 ? ? 1 ? ? ? ? 0 ? 1 0 1 0 2 1 0 0 0 ? 1 0 0 0 0 1 0 1 1 1 0 ? 0 ? 1 0 1 ? ? ? ? ? 1 ? ? ? ? ? 1 ? ? ? ? ? ? ? ? ? 0 ? ? ? 1 0 ? ? ? ? ? ? ? 0 ? 1 ? 1 ? 1 0 ? 1 ? ? 0 ? ? ? ? ? ? ? ? ? ? ? ? ? ? ? ? ? ? ? ? ? 1 ? 1 ? ? ? ? 0 1 0 1 3 0 ? 1 ? 0 ? ? ? 0 0 ? 1 0 ? ? ? ? ? 1 0 0 1 1 ? ? ? ? 1 2 0 1 1 ? ? 0 0 0 ? 1 0 0 0 0 0 0 1 0 0 1 0 0 0 0 0 1 0 0 0 0 0 0 0 0 0 ? 0 0 0 ? 1 0 ? ? ? ? ? ? ? ? 0 1 0 ? ? ? ? ? 0 2 ? ? ? ? ? ? ? ? ? ? ? ? ? ? ? ? ? ? ? ? ? ? ? ? ? ? ? ? ? ? ? ? ? ? ? ? ? ? ? ? ? ? ? ? ? ? ? ? ? ? ? ? ? ? ? ? ? ? ? ? ? ? ? ? ? ? ? ? ? ? ? ? ? ? ? ? ? ? ? ? ? ? ? ? ? ? ? ? ? ? ?

Nemegtomaia 1 1 0 0 0 0 0 0 1 1 0 1 1 1 0 1 2 0 ? 1 0 0 1 1 0 0 1 0 0 1 1 1 1 0 0 1 1 0 0 0 1 3 2 0 0 1 1 0 1 1 0 1 1 1 1 1 1 0 0 0 0 01 ? 0 ? 1 1 ? 0 0 1 1 1 ? 1 ? ? ? ? ? ? 0 0 ? 1 ? ? ? 1 1 0 ? ? ? 0 ? 1 1 1 1 0 ? 1 2 0 2 1 0 1 0 2 1 0 0 0 ? 1 0 0 0 0 1 0 1 1 ? 0 ? 0 1 1 0 1 ? ? ? ? ? 1 ? ? ? ? ? 1 ? ? ? ? ? ? ? ? ? 1 ? 0 1 1 0 ? 0 0 ? 0 1 1 0 0 1 1 1 0 0 0 ? ? ? ? ? 2 ? 1 2 0 ? ? ? ? ? ? ? ? ? ? ? ? ? ? ? ? ? ? ? ? ? ? ? ? ? ? ? ? ? ? ? ? ? ? ? ? ? ? ? 0 0 0 0 0 0 ? 0 ? ? ? ? ? ? ? ? ? ? ? ? ? ? ? ? ? ? ? ? ? ? ? ? ? ? ? ? ? ? ? ? ? ? ? ? ? ? ? ? ? ? ? ? ? ? ? ? ? ? ? ? 0 0 1 0 0 1 1 0 ? ? 0 1 ? 1 0 ? 0 ? ? ? 1 0 ? ? ? ? ? ? ? ? ? 0 0 1 ? 0 ? ? ? ? ? ? ? ? ? ? ? ? ? ? 1 1 1 1 0 ? 0 ? ? ? ? ? ? ? ? ? ? ? ? ? ? ? ? ? ? ? ? ? ? ? ? ? ? ? ? ? ? ? ? ? ? ? ? ? ? ? ? ? ? ? ? ? ? ? ? ? ? ? ?

Rinchenia ? 1 0 ? 0 0 0 0 1 1 0 1 1 ? ? 1 ? 0 ? ? ? 0 1 1 0 ? ? ? ? 1 ? 1 1 ? 0 ? 1 0 0 0 1 3 ? 0 0 1 ? 0 1 1 0 1 1 1 1 1 1 0 ? 0 0 0? ? 0 ? 1 ? ? 0 0 ? ? ? ? ? ? ? ? ? ? 0 ? ? ? ? ? ? ? ? ? 0 ? 1 ? ? ? ? 1 1 1 0 ? ? 2 0 2 1 0 1 0 2 1 0 0 0 ? 1 0 0 0 0 1 0 1 1 1 0 ? ? 1 1 0 1 ? ? ? ? ? 1 ? ? ? ? ? 1 ? ? ? ? ? ? ? ? ? 1 ? ? ? ? ? ? ? ? ? ? 0 ? ? ? ? 1 1 ? ? ? ? ? ? ? ? 1 ? ? ? 0 1 1 ? ? ? ? ? ? ? ? ? ? ? ? ? ? ? ? ? ? ? ? 1 ? 1 ? 1 ? 0 0 1 ? ? ? ? ? ? 0 1 ? 0 ? ? ? ? ? ? ? ? ? ? 0 ? ? ? 1 ? 0 ? ? ? ? ? ? ? ? ? ? ? ? ? ? ? ? ? ? ? ? ? ? ? ? ? ? ? ? ? ? ? 0 ? 0 ? ? ? ? ? ? ? ? ? 1 0 0 ? ? 0 0 1 0 1 ? ? 0 ? 0 ? ? ? ? ? ? ? ? ? ? ? ? ? ? ? ? ? ? ? ? ? ? ? ? ? ? ? ? ? ? ? ? ? ? 1 ? ? ? ? 0 ? ? 0 ? 0 ? ? ? ? ? ? ? ? ? ? ? ? ? 0 ? ? ? ? ? ? ? ? ? ? ? ? ? 0 ? ? ? ? ? ? ? ? ? ? ? ? ? ? ? ? ? ? ?

IGM_100_42 1 1 0 0 0 0 0 0 1 1 0 1 1 1 0 1 2 0 ? 1 0 0 1 1 0 ? 1 0 0 1 1 1 1 0 0 1 1 0 0 0 1 3 2 0 0 1 1 0 1 1 0 1 1 1 1 1 1 ? 0 0 0 0? ? 0 ? 1 1 ? 0 0 1 1 1 ? ? ? ? ? ? ? ? ? 0 ? ? ? ? ? 1 1 0 ? 1 ? 0 ? 1 1 1 1 0 ? 1 2 0 2 1 0 1 ? 2 1 0 0 0 ? 1 0 0 0 0 1 0 1 1 1 0 ? ? 1 1 0 1 ? ? ? ? ? 1 ? ? ? ? ? 1 ? ? ? ? ? ? ? ? ? 1 ? 0 1 ? 0 ? 0 0 ? ? 0 1 0 ? 1 1 1 ? 0 0 1 1 ? ? ? 1 ? ? ? 0 1 1 0 ? 0 ? 0 0 2 ? 0 0 0 ? 1 ? ? ? 1 0 1 ? 1 0 1 0 1 3 0 0 1 ? 0 1 ? 1 0 0 1 0 0 ? 0 0 0 1 1 ? ? ? 1 0 ? ? ? 1 2 0 0 1 0 0 0 0 0 0 ? 0 0 0 ? 0 0 1 0 0 1 0 0 0 0 0 1 0 0 0 0 0 0 0 0 0 0 0 0 0 1 1 1 0 0 1 0 0 1 1 0 0 1 ? 1 0 1 0 0 0 2 1 0 1 0 1 0 ? ? ? ? ? 0 1 0 0 1 0 0 2 0 0 ? 1 1 0 0 0 1 ? 1 1 ? 1 1 ? ? 0 0 0 1 0 0 ? ? ? ? ? ? ? ? ? 0 1 0 1 0 1 0 0 0 0 0 0 0 0 1 0 0 0 1 0 0 1 ? 0 0 0 0 0 0 0 0 0 ? ? ? 0 0 0 0 0

Citipati 1 1 0 0 0 0 0 0 1 1 0 1 1 1 0 1 2 0 ? 1 0 0 1 1 0 0 1 0 0 1 1 1 1 0 0 1 1 0 0 0 1 3 2 0 0 1 1 0 1 1 0 1 1 1 1 1 1 0 0 0 0 01 1 0 ? 1 1 0 0 0 1 1 1 0 ? 1 2 2 0 0 1 0 0 0 1 ? ? 0 1 1 0 1 1 0 0 0 1 1 1 1 0 0 0 2 0 2 1 0 1 0 2 1 0 0 0 ? 1 ? 0 0 0 1 0 1 1 1 0 1 0 1 1 0 1 ? ? ? ? ? 1 ? ? ? ? ? 1 ? ? ? ? ? ? ? ? ? 1 1 0 1 1 0 1 0 0 0 0 ? 1 1 0 ? 1 1 0 ? 0 1 ? 1 1 0 1 ? ? 2 0 1 ? ? ? ? ? ? 0 2 ? ? ? 0 0 ? 1 1 ? 1 0 1 1 1 0 1 0 1 3 0 0 0 ? 0 1 0 ? 0 0 1 0 0 0 0 0 0 1 1 0 0 1 1 0 ? 0 1 1 2 0 0 1 0 0 0 0 0 0 1 0 0 0 0 0 0 1 0 0 0 0 0 0 0 0 1 0 0 0 0 0 0 0 0 1 0 0 0 0 0 1 1 0 0 1 0 ? 1 1 0 0 ? 0 1 0 1 ? ? 0 2 ? 0 1 ? 1 ? 0 0 0 ? ? 0 ? 0 ? 1 0 ? 2 0 0 0 1 1 0 ? ? ? ? 1 1 ? 1 ? 1 1 0 0 0 1 0 0 ? 0 0 0 1 0 ? 1 0 0 1 0 1 0 1 0 0 0 0 0 0 0 0 1 0 0 0 ? 0 0 ? 0 0 1 0 0 0 0 0 0 0 1 0 0 0 0 0 0 0

Chirostenotes ? ? ? ? ? ? ? ? ? ? ? ? ? ? ? ? ? ? ? ? ? ? ? ? ? ? ? ? ? ? ? ? ? ? ? ? ? ? ? ? ? ? ? ? ? ? ? ? ? ? ? ? ? ? ? ? ? ? ? ? ? ?? ? ? ? ? ? ? ? ? ? ? ? ? ? ? ? ? ? ? ? ? ? ? ? ? ? ? ? ? ? ? ? ? ? ? ? ? ? ? ? ? ? ? ? ? ? ? ? ? ? ? ? ? ? ? ? ? ? ? ? ? ? ? ? ? ? ? ? ? ? ? ? ? ? ? ? ? ? ? ? ? ? ? ? ? ? ? ? ? ? ? ? ? ? ? ? ? ? ? ? ? ? ? ? ? ? ? ? ? ? ? ? ? ? ? ? ? ? ? 1 ? 1 2 ? ? 1 ? ? ? ? ? ? ? ? ? ? ? ? ? ? ? ? ? ? ? ? ? ? ? ? ? ? 0 ? 0 ? ? 1 0 1 0 ? ? ? ? ? 0 ? 0 ? ? ? ? ? ? ? ? ? ? ? ? ? ? 1 ? 0 ? 0 ? 0 ? ? ? ? 0 0 0 ? ? ? 0 0 0 1 1 0 0 1 0 0 0 ? 0 0 0 0 ? 1 0 0 1 1 0 ? 0 1 0 0 1 ? 0 0 1 0 1 0 ? 0 0 0 2 1 ? ? ? ? ? ? ? ? ? ? ? ? 0 ? 1 0 ? 2 0 ? 0 1 1 0 0 0 1 1 1 1 ? ? ? ? ? 0 ? 0 ? ? ? ? 0 ? ? ? ? 1 1 0 0 1 0 1 0 1 ? 0 0 0 0 0 0 0 1 0 0 0 ? 0 0 ? 0 0 1 0 0 0 0 0 0 0 1 0 0 0 0 0 0 0

Hagryphus ? ? ? ? ? ? ? ? ? ? ? ? ? ? ? ? ? ? ? ? ? ? ? ? ? ? ? ? ? ? ? ? ? ? ? ? ? ? ? ? ? ? ? ? ? ? ? ? ? ? ? ? ? ? ? ? ? ? ? ? ? ?? ? ? ? ? ? ? ? ? ? ? ? ? ? ? ? ? ? ? ? ? ? ? ? ? ? ? ? ? ? ? ? ? ? ? ? ? ? ? ? ? ? ? ? ? ? ? ? ? ? ? ? ? ? ? ? ? ? ? ? ? ? ? ? ? ? ? ? ? ? ? ? ? ? ? ? ? ? ? ? ? ? ? ? ? ? ? ? ? ? ? ? ? ? ? ? ? ? ? ? ? ? ? ? ? ? ? ? ? ? ? ? ? ? ? ? ? ? ? ? ? ? ? ? ? ? ? ? ? ? ? ? ? ? ? ? ? ? ? ? ? ? ? ? ? ? ? ? ? ? ? ? ? ? ? ? ? ? ? ? ? ? ? ? ? ? ? ? ? ? ? ? ? ? ? ? ? ? ? 1 1 0 0 1 0 0 ? 0 0 ? 1 0 0 1 0 0 0 1 0 ? 0 0 0 1 1 0 0 1 0 0 0 0 0 0 0 0 0 ? 0 0 1 1 ? ? ? ? ? ? ? ? ? ? ? ? ? ? ? ? ? ? ? ? ? ? ? ? ? ? ? ? ? ? ? ? ? ? ? ? ? ? ? ? ? ? ? ? ? ? ? ? ? ? ? ? ? ? ? ? ? 0 ? ? ? ? ? ? ? ? ? ? ? ? ? ? ? ? ? ? ? ? ? ? ? ? ? ? ? ? ? ? ? ? ? ? ? ? ? ? ? ? ? ? ? ? ? ? ? ? ? ? ? ?

Elmisaurus ? ? ? ? ? ? ? ? ? ? ? ? ? ? ? ? ? ? ? ? ? ? ? ? ? ? ? ? ? ? ? ? ? ? ? ? ? ? ? ? ? ? ? ? ? ? ? ? ? ? ? ? ? ? ? ? ? ? ? ? ? ?? ? ? ? ? ? ? ? ? ? ? ? ? ? ? ? ? ? ? ? ? ? ? ? ? ? ? ? ? ? ? ? ? ? ? ? ? ? ? ? ? ? ? ? ? ? ? ? ? ? ? ? ? ? ? ? ? ? ? ? ? ? ? ? ? ? ? ? ? ? ? ? ? ? ? ? ? ? ? ? ? ? ? ? ? ? ? ? ? ? ? ? ? ? ? ? ? ? ? ? ? ? ? ? ? ? ? ? ? ? ? ? ? ? ? ? ? ? ? ? ? ? ? ? ? ? ? ? ? ? ? ? ? ? ? ? ? ? ? ? ? ? ? ? ? ? ? ? ? ? ? ? ? ? ? ? ? ? ? ? ? ? ? ? ? ? ? ? ? ? ? ? ? ? ? ? ? ? ? ? ? ? 0 1 ? 0 ? 0 ? ? ? ? ? ? 0 0 0 ? ? ? 0 0 0 1 1 0 ? 1 0 0 0 0 0 1 0 ? 0 ? 0 ? 1 ? ? ? ? ? ? ? ? ? ? ? ? ? ? ? ? ? ? ? ? ? ? ? ? ? ? ? ? ? ? ? ? ? ? ? ? ? ? ? ? ? ? ? ? ? ? ? ? ? ? ? ? ? ? ? ? ? ? 0 ? ? ? ? ? ? ? ? ? ? ? ? 1 ? 0 1 0 1 0 ? ? ? 0 0 0 0 1 1 0 0 1 ? 0 ? ? 0 1 0 0 0 0 0 0 0 1 0 0 0 0 0 0 0

Conchoraptor ? 1 0 0 0 0 0 0 1 1 0 1 1 1 0 1 2 0 ? 1 ? 0 ? ? ? 0 1 ? 0 1 ? 1 1 0 0 1 0 0 0 0 1 3 2 0 0 1 1 0 1 1 0 1 1 1 1 1 1 0 0 0 0 0? ? 0 ? 1 1 0 0 0 1 1 1 0 ? ? 1 ? ? ? ? ? 0 ? ? ? ? ? ? 1 ? ? 1 ? ? 0 ? ? 1 1 0 ? ? 2 ? 2 1 ? ? ? 2 ? 0 0 0 ? 1 ? ? 0 0 ? 0 1 ? 0 0 ? 0 1 1 0 1 ? ? ? ? ? 1 ? ? ? ? ? 1 ? ? ? ? ? ? ? ? ? ? ? ? ? ? ? ? ? ? ? ? 1 1 ? 0 ? 1 1 0 ? ? ? ? ? ? 0 2 ? 1 1 0 0 1 ? 1 ? ? ? ? ? ? ? ? ? 0 ? 1 1 ? ? ? ? 1 ? 0 1 ? 1 ? ? 0 1 ? 0 1 ? ? ? 0 1 ? 0 0 ? 0 ? ? ? ? ? ? 1 0 ? ? ? ? ? ? ? 1 ? 0 ? 0 0 ? 1 ? ? 0 0 0 0 ? 0 ? ? ? 0 0 0 ? ? 0 ? 0 0 0 0 0 0 1 0 0 1 1 1 1 0 0 0 ? ? 0 ? 1 0 0 1 0 1 0 ? ? ? 0 2 1 0 1 ? 1 ? 0 0 ? ? ? 0 ? 0 ? 1 0 ? 2 0 0 0 ? ? ? ? ? ? ? ? ? ? 1 1 1 1 0 0 ? 1 ? 0 ? 0 ? ? ? ? ? ? 0 0 ? ? 1 0 0 0 0 ? 0 0 0 0 0 0 0 0 ? ? 0 0 1 ? ? ? ? ? ? ? ? ? ? ? ? ? ? ? 0 0 0

Khaan 1 1 0 0 0 0 0 0 1 1 0 1 1 1 0 1 2 0 ? 1 0 0 1 1 0 0 1 0 0 1 ? 1 1 0 0 1 0 0 0 0 1 3 2 0 0 1 1 0 1 1 0 1 1 1 1 1 1 0 0 0 0 0? ? 0 ? 1 1 0 0 0 1 1 1 ? ? ? ? ? ? ? ? ? ? ? ? ? ? ? 1 1 0 ? 1 0 0 ? ? ? ? 1 ? ? ? ? 0 2 1 0 1 0 2 1 0 0 0 ? 1 0 0 0 0 1 0 1 1 ? 0 ? 0 1 1 0 1 ? ? ? ? ? ? ? ? ? ? ? 1 ? ? ? ? ? ? ? ? ? 1 1 0 1 1 0 ? 0 0 ? ? 1 1 0 ? 1 1 1 0 1 0 1 1 1 1 0 ? ? ? ? ? 1 1 0 ? 0 ? 0 0 2 ? 0 0 0 ? ? 1 1 0 1 0 1 ? ? 0 1 0 1 3 0 0 1 ? 0 1 0 1 0 0 1 0 0 0 0 0 0 1 1 0 0 1 1 0 ? 0 1 1 2 0 0 1 0 0 0 0 0 0 1 0 0 0 0 0 0 1 0 0 0 0 0 0 0 0 1 0 0 0 0 0 0 0 0 1 0 0 0 1 1 1 0 0 0 1 0 0 1 1 0 0 1 ? 1 0 1 0 1 0 2 1 0 1 0 1 0 ? ? ? 0 0 ? 1 0 ? 1 0 0 2 0 0 0 1 1 0 0 0 1 ? 1 1 ? 1 1 1 1 0 0 0 1 0 ? ? 0 0 ? ? 0 1 1 0 0 1 0 1 0 0 0 0 0 0 0 0 0 0 0 0 0 0 ? 0 0 1 0 0 0 0 0 0 0 0 0 0 1 0 0 0 0 0 0 0

Heyuannia 1 ? ? ? ? ? ? ? ? ? ? ? ? ? ? ? ? ? ? ? ? ? ? ? ? ? ? ? ? ? ? ? ? ? ? ? 0 ? ? ? ? ? ? ? ? 1 1 ? ? ? ? ? ? ? ? ? ? ? ? ? ? ?? ? ? ? ? ? ? ? ? ? ? ? ? ? ? ? ? ? ? ? ? ? ? ? ? ? ? ? ? ? ? ? ? ? ? ? ? ? ? ? ? ? ? 0 ? 1 ? 1 0 2 1 0 0 0 ? 1 ? ? ? ? ? 0 1 1 1 0 ? ? ? 1 0 ? ? ? ? ? ? ? ? ? ? ? ? 1 ? ? ? ? ? ? ? ? ? 1 ? ? ? ? 0 ? ? ? ? ? ? 1 0 ? ? ? ? ? 1 0 ? 1 ? ? 0 2 ? ? ? ? ? 1 ? ? ? ? ? ? ? ? 0 1 0 ? ? 1 1 ? ? ? ? ? ? 0 1 0 1 3 0 ? ? ? 0 1 ? ? 0 0 ? 0 0 ? 0 ? 0 ? 1 0 0 1 1 0 ? 0 ? 1 2 1 1 1 0 0 ? 0 0 0 ? 0 0 0 0 1 0 1 0 1 0 0 1 0 ? 0 ? ? ? ? ? ? 0 0 0 1 0 ? 0 1 0 ? ? 0 0 1 0 0 ? ? 0 0 1 ? 1 0 ? 0 1 0 2 1 0 1 0 1 0 ? ? ? ? ? ? 0 0 ? 1 0 0 2 0 0 ? 1 1 0 0 0 1 1 1 1 ? 1 1 ? ? 0 0 0 1 0 0 ? 0 0 ? ? ? ? ? ? ? 1 ? 1 0 0 0 0 0 0 0 ? 0 0 0 0 0 ? ? ? 0 ? ? 0 0 0 0 ? 0 0 0 0 ? 0 0 0 0 0 0 0

Ingenia 1 1 0 0 0 0 0 0 1 1 0 1 1 1 ? 1 2 0 ? 1 0 0 1 1 0 0 1 0 0 1 1 1 1 ? 0 ? 0 0 ? ? 1 3 2 0 0 1 ? 0 1 1 0 1 1 1 1 ? 1 ? ? 0 ? 0? ? 0 ? 1 ? ? 0 ? ? 1 ? ? ? ? ? ? ? ? ? ? 0 ? ? ? ? ? 1 1 ? ? 1 0 0 ? ? ? 1 1 ? ? 1 2 0 2 1 0 1 0 2 1 0 0 0 ? 1 0 0 0 0 1 0 1 1 1 0 ? 0 1 1 0 1 ? ? ? ? ? 1 ? ? ? ? ? 1 ? ? ? ? ? ? ? ? ? ? ? ? 1 ? ? ? 0 0 ? ? 0 ? ? ? ? 1 1 ? ? ? ? ? ? ? ? 2 ? ? ? 0 1 1 0 ? 0 ? 0 ? 2 ? 0 1 0 0 ? ? ? 0 1 0 1 1 1 0 1 0 1 3 0 0 1 ? ? 1 0 1 0 0 1 0 0 0 0 0 0 1 1 0 0 ? 1 0 ? 0 ? 1 2 0 0 1 0 0 0 0 0 0 1 0 0 0 0 1 0 1 0 1 0 0 1 0 0 0 1 0 0 0 0 0 0 0 0 1 0 0 0 1 0 1 0 0 0 1 0 0 1 ? 0 0 1 ? 1 0 1 0 ? 0 2 1 0 1 0 1 0 ? ? ? 0 0 0 0 0 0 1 0 0 2 0 0 0 1 1 0 0 0 1 1 1 1 0 1 1 1 1 0 0 0 1 0 0 ? 0 0 0 ? 0 1 1 0 0 1 0 1 0 0 0 0 0 0 0 0 0 0 0 0 0 0 1 0 0 ? 0 0 0 0 ? ? ? ? 0 0 1 ? ? 0 0 0 0 0

Epidendrosaurus 0 ? 0 ? 0 ? ? ? ? ? ? ? ? ? ? ? ? ? ? ? ? ? ? ? ? ? ? ? ? ? ? ? ? ? ? ? 0 0 0 0 0 0 ? 0 0 ? ? ? ? 1 0 1 0 1 0 ? 1 ? ? ? ? ?? ? 1 1 1 0 0 0 ? 0 ? ? ? ? ? ? ? ? ? ? ? ? ? ? ? ? ? ? ? ? ? ? ? ? ? ? ? ? ? ? ? ? ? 0 2 0 0 0 0 0 1 0 0 0 1 0 0 0 0 1 ? 0 0 0 0 0 ? 0 0 0 0 ? ? ? ? ? ? ? ? 0 ? ? 1 0 0 1 ? ? ? ? ? ? ? ? ? ? ? ? ? ? ? ? ? ? ? ? 0 ? 1 ? 0 0 1 0 0 1 ? 1 0 0 0 ? ? ? 1 0 0 ? 2 1 1 0 0 ? 0 0 1 ? 1 ? ? 1 ? ? ? ? ? ? ? 0 ? 0 ? ? 0 ? 1 1 0 1 0 1 2 1 0 0 0 ? 0 2 1 0 0 1 0 0 ? 0 ? 1 2 1 0 1 0 ? 1 0 0 0 ? 0 0 0 0 0 0 1 0 0 1 0 0 0 0 0 1 0 0 0 0 0 0 0 0 0 0 0 0 0 0 0 0 0 1 0 0 1 ? ? 1 1 1 ? 1 0 ? 0 1 0 ? 1 1 1 ? 0 0 ? ? ? 0 0 2 ? 0 0 ? ? ? 2 0 0 ? 2 ? ? ? ? ? ? 1 1 ? 1 1 ? ? 0 ? 0 1 0 ? ? 0 0 ? ? ? ? ? 0 0 1 ? 1 0 1 0 0 1 0 0 0 0 0 0 0 0 ? ? 0 0 ? 1 0 0 0 2 0 0 0 0 0 1 1 0 0 0 1 1 0

Epidexipteryx 0 ? 0 ? 0 ? 1 0 ? ? ? 0 ? 1 ? ? ? 0 ? ? ? 0 1 1 0 ? ? ? ? 1 ? ? 0 ? ? ? ? 0 0 0 ? ? ? 0 0 ? ? ? ? ? ? 1 ? ? 0 ? 0 ? ? ? ? 00 0 1 1 1 0 0 0 ? 0 1 1 ? ? ? ? ? ? ? ? ? ? ? ? ? ? ? ? ? ? ? ? ? ? ? ? ? ? ? ? ? ? ? 0 ? 1 0 0 0 0 ? ? ? 0 1 0 ? ? ? 1 ? 0 0 0 ? ? ? 0 0 0 0 0 1 3 0 0 0 0 2 0 0 2 1 0 0 1 0 ? ? ? 1 ? 0 0 ? ? ? ? ? ? ? ? ? ? 0 1 0 ? 1 0 0 ? 1 0 ? 1 ? ? 0 1 ? ? ? ? 3 ? 0 ? 2 2 1 0 2 ? ? 0 ? ? ? 0 0 ? 0 ? ? ? ? ? ? 0 ? 0 ? ? 0 ? ? 1 ? 1 0 1 2 1 0 0 0 0 0 2 1 0 0 1 1 0 ? ? ? ? ? ? ? 1 ? ? ? ? ? ? ? 0 ? ? ? ? 0 1 ? 0 ? ? ? ? ? ? ? ? 0 ? ? ? 0 0 0 ? 0 0 0 0 ? 0 ? ? 1 0 0 1 ? ? 1 ? 1 ? ? 0 0 0 ? 0 ? ? 1 0 ? 0 0 ? ? ? 0 0 2 ? 0 ? 1 0 0 2 0 0 ? 2 ? ? ? ? ? 1 1 1 ? ? ? ? 1 ? ? 0 ? ? ? ? ? 0 ? ? 0 1 ? 1 1 1 0 1 0 1 0 0 1 0 0 0 ? ? ? 0 0 ? 1 0 ? 1 ? ? ? ? ? ? ? ? ? ? ? ? ? ? ? ? ? ?

Archaeopteryx 0 0 0 1 0 0 1 1 0 0 0 0 1 1 1 0 1 0 0 1 0 0 1 1 0 1 1 0 1 1 ? ? 0 0 0 0 0 0 0 0 0 0 2 0 0 1 1 0 1 1 0 1 0 1 0 0 0 0 ? 0 ? ?0 0 1 1 1 0 0 0 0 0 0 0 ? 0 ? 1 ? 0 ? ? 0 ? 0 0 ? 1 ? 1 0 0 ? ? ? 0 ? 1 ? 0 ? 1 1 0 1 0 0 0 0 0 0 ? ? 0 1 0 1 0 0 0 0 ? 0 1 ? ? 0 0 2 0 0 0 0 0 1 3 0 0 0 0 2 0 0 2 1 0 0 0 0 ? ? ? 0 1 0 0 1 0 1 ? ? ? 0 0 1 0 0 0 1 0 1 0 0 0 1 0 ? 1 ? ? ? 0 ? ? ? 0 1 0 0 1 2 2 1 0 2 ? 1 0 1 1 1 0 0 1 0 ? ? ? ? 0 0 0 1 0 0 1 0 ? 1 1 0 1 1 1 2 1 0 0 0 0 0 3 1 0 0 1 1 0 ? 0 ? 1 2 0 0 1 0 0 0 0 0 1 ? 0 0 0 0 0 0 1 0 0 1 0 0 0 1 0 0 0 0 0 2 0 0 0 0 0 0 1 0 0 0 1 0 0 1 0 0 1 1 ? 1 1 1 0 1 0 ? 0 1 0 2 0 0 1 0 0 0 0 1 1 0 0 1 1 0 0 1 0 ? 0 0 2 ? 1 1 0 1 1 0 ? 1 1 ? 1 1 1 1 0 1 0 1 0 0 ? 0 ? 0 ? 0 1 1 0 0 1 0 1 0 2 0 0 1 0 0 0 0 0 1 0 0 0 ? 1 0 1 1 0 1 0 2 0 0 0 0 0 1 1 1 0 0 1 1 0

Wellnhoferia 0 0 0 1 0 0 1 1 0 0 0 0 ? 1 1 ? 1 0 0 ? ? 0 ? ? ? ? ? ? ? ? ? ? ? ? ? ? ? 0 ? ? ? ? ? ? ? ? ? ? ? ? ? ? ? ? 0 ? ? ? ? ? ? ?? ? ? ? ? ? ? ? ? ? ? ? ? ? ? ? ? ? ? ? ? ? ? ? ? ? ? ? ? ? ? ? ? ? ? ? ? ? ? ? ? ? ? 0 0 0 0 0 0 ? ? 0 1 0 1 ? ? ? ? ? ? 1 ? ? ? ? ? ? ? ? ? 0 1 3 0 0 0 0 2 0 0 2 1 0 0 0 0 ? ? ? 0 ? ? ? ? ? ? ? ? ? ? ? ? ? ? ? ? ? 1 ? ? ? 1 0 ? ? ? ? ? 0 ? ? ? ? 3 0 0 ? 2 2 1 0 2 ? 1 0 1 ? 1 0 0 ? 0 ? ? ? ? ? ? 0 ? 0 0 1 0 ? 1 1 0 1 ? 1 2 1 0 0 0 0 0 3 1 0 0 1 1 0 ? ? ? ? ? ? 0 1 0 0 0 0 0 1 ? 0 0 0 0 0 0 1 1 0 1 0 0 0 1 0 0 0 0 0 2 0 0 0 0 0 0 1 0 0 0 1 0 0 ? 0 0 1 1 ? 1 1 1 ? ? 0 ? 0 1 0 ? ? 0 1 0 0 0 ? ? 1 0 0 1 1 0 0 1 ? ? 0 ? 2 ? ? ? ? ? ? ? ? 1 1 ? 1 1 ? ? 0 1 0 1 0 ? ? ? 0 ? ? 0 1 ? 0 0 1 ? 1 0 2 ? 0 1 0 0 0 ? ? 1 ? 0 ? ? ? 0 1 1 0 ? 0 2 0 0 0 0 0 1 1 1 0 ? ? 1 ?

Jeholornis 0 0 0 ? 0 0 1 0 0 0 0 0 ? 1 1 ? 1 0 0 ? ? 0 1 1 0 ? ? ? ? 1 ? 0 ? 0 ? 0 0 0 ? ? ? 0 ? 0 0 ? ? ? ? 1 0 1 0 1 0 ? ? ? ? ? ? ?? ? 1 ? 1 ? ? 0 ? 0 ? ? ? ? ? ? ? ? ? ? ? ? ? ? ? ? ? ? ? ? ? ? ? ? ? ? ? ? ? ? ? ? ? 0 0 2 0 0 0 ? 0 0 1 0 1 0 0 0 0 1 ? 1 ? 0 0 0 2 0 0 0 0 1 ? ? ? ? ? 1 2 1 ? 2 1 0 0 ? ? ? ? ? ? 1 ? 0 ? ? ? ? ? ? 0 0 ? ? ? 1 1 0 1 ? ? ? 1 0 ? 1 ? ? 0 1 ? ? ? ? 1 0 0 1 2 2 1 0 2 ? 0 0 0 1 1 ? ? ? 1 ? 1 ? 1 ? ? 0 ? 0 0 1 0 1 ? 3 0 1 1 1 2 1 0 0 0 0 0 3 1 0 0 1 1 0 ? 0 ? 1 2 0 1 1 0 0 0 0 0 1 ? 0 0 0 0 0 1 1 1 0 0 1 1 0 0 0 0 1 0 0 0 1 0 0 0 0 0 0 0 0 0 0 0 0 1 0 0 1 1 ? 1 1 1 ? 1 0 ? 0 1 0 2 ? 0 2 0 0 0 0 1 1 0 0 ? ? 0 0 1 0 0 0 1 0 ? ? ? ? ? ? ? ? ? 1 ? ? ? ? ? ? ? 0 1 0 0 ? 0 0 ? ? 0 1 1 0 1 1 0 1 0 1 0 0 1 0 0 0 ? ? 0 0 0 0 ? 1 0 1 1 0 0 0 2 0 0 0 0 0 1 1 1 0 0 1 1 0

Sapeornis 0 0 0 ? 0 0 1 0 0 0 0 0 0 1 1 0 1 0 0 ? ? 0 1 1 0 ? ? ? ? 1 ? ? 0 0 0 0 0 0 0 0 0 0 2 0 0 1 1 0 1 1 0 1 1 1 0 0 0 ? ? ? ? 0? 0 1 ? 1 ? 0 0 ? 0 0 1 ? ? ? ? ? ? ? ? ? ? ? ? ? ? ? ? ? ? ? ? ? ? ? ? ? ? ? ? ? ? ? 0 0 0 0 0 0 ? 0 0 1 0 1 0 0 0 0 1 0 1 ? 0 ? 0 ? 0 0 0 0 0 1 3 0 0 ? ? ? ? ? ? 1 0 ? ? ? ? ? ? 0 ? ? 0 ? 0 1 ? ? ? ? ? ? ? 0 1 1 0 1 ? 1 0 1 0 0 1 ? 1 0 2 ? ? ? 0 2 0 0 1 ? ? ? ? ? ? 0 0 0 ? ? 0 1 ? ? ? ? ? ? 0 1 0 1 0 0 1 0 1 1 1 0 0 1 1 2 1 0 0 0 1 0 3 1 1 0 1 1 0 ? 0 ? 1 2 2 1 1 0 ? 0 0 0 1 1 1 0 0 0 0 0 1 1 0 0 1 1 1 1 0 ? 1 0 ? ? ? 0 0 0 0 0 0 0 0 0 0 ? 0 1 0 0 1 1 ? 1 1 1 0 1 0 ? 0 1 0 2 1 0 2 0 0 0 0 1 1 0 0 1 1 0 0 1 0 0 0 1 0 ? 2 ? ? ? ? ? ? 1 1 ? ? 1 ? ? 0 ? 0 1 0 ? ? 0 0 ? ? ? ? 1 1 1 1 0 1 0 1 0 0 1 0 0 0 0 0 0 0 0 0 1 0 0 1 1 ? ? ? ? ? ? ? 0 0 1 1 1 0 0 1 1 0

Confuciusornis 0 0 0 0 0 0 1 1 0 0 ? 0 0 1 1 ? ? 0 0 ? 0 0 ? ? ? ? ? ? ? ? ? 0 0 0 0 0 0 0 0 0 ? 0 2 0 0 1 1 0 1 1 0 1 1 1 0 0 ? ? 0 0 0 0? ? 1 1 1 ? ? 0 ? 0 0 1 ? ? ? ? ? ? ? ? ? ? ? ? ? ? ? 1 ? ? ? ? ? 0 ? ? ? ? ? ? ? ? ? 0 0 0 0 0 0 1 1 0 0 0 1 0 0 1 0 1 0 0 0 0 ? 0 ? 0 0 0 0 1 ? ? ? ? ? 1 ? ? ? ? ? 1 ? ? ? ? ? ? ? ? ? 0 ? ? 1 ? 1 ? 1 0 ? ? 0 1 1 0 1 ? 1 0 1 0 0 1 1 1 0 2 ? ? ? 0 2 ? 0 1 ? ? ? ? ? ? ? 0 ? ? ? 1 1 ? 1 1 1 0 1 0 0 0 ? 0 0 1 1 1 1 3 0 0 1 1 2 1 0 0 0 1 0 2 1 1 0 1 1 0 1 0 1 1 2 0 1 1 0 0 0 0 0 1 1 0 0 0 0 0 0 1 0 0 0 1 0 0 1 0 0 1 0 0 0 1 0 0 0 0 1 1 0 0 1 1 1 0 1 0 0 1 ? ? 1 1 1 ? 1 0 1 0 ? 0 2 ? 0 3 0 0 0 0 1 1 0 0 2 1 0 ? 1 ? 0 0 1 0 0 2 ? ? ? ? ? 2 1 1 1 1 1 ? 1 0 ? 0 1 ? 0 ? 1 1 1 ? 2 1 1 1 1 1 0 1 0 1 1 0 1 0 0 0 0 0 1 0 0 0 ? 1 0 1 1 0 0 0 2 0 0 0 0 0 1 1 1 0 0 1 1 0

Protopteryx 0 0 0 1 0 ? 1 1 0 0 ? ? ? 1 1 ? ? 0 0 ? ? ? ? ? ? ? ? ? ? 1 ? ? ? 1 0 0 0 0 0 0 ? ? ? 1 ? ? ? ? ? ? ? 1 ? 1 0 ? ? ? ? ? ? 1? ? 1 ? 1 ? ? ? ? 0 ? ? ? ? ? ? ? ? ? ? ? ? ? ? ? ? ? ? ? ? ? ? ? ? ? ? ? ? ? ? ? ? ? 0 0 0 0 0 0 ? ? ? ? ? 1 0 ? ? ? ? ? ? ? ? ? ? ? ? ? 0 0 0 1 3 ? 0 ? ? 2 ? ? 2 1 0 0 ? ? ? ? ? 0 ? ? ? ? ? ? ? ? ? ? ? ? ? ? 1 1 0 ? ? ? ? ? ? ? ? ? 1 ? 2 ? ? ? ? 2 ? 0 ? ? ? ? ? ? ? ? 0 ? ? ? 1 1 ? 1 1 1 ? 1 1 1 0 ? 0 ? ? 0 ? 1 3 0 ? 1 1 2 1 0 0 0 ? 0 3 1 1 0 1 1 0 ? 0 ? 1 2 2 1 ? 0 ? 0 0 0 1 ? 0 0 0 ? 0 0 1 1 0 0 1 1 0 0 0 ? 1 0 1 ? 1 0 0 0 0 1 1 0 0 0 0 ? ? ? 0 0 ? ? ? 1 ? ? ? ? ? ? ? ? ? ? ? ? ? ? ? ? ? ? ? 0 0 ? 1 0 ? ? ? ? ? ? ? ? ? ? ? ? ? ? ? 1 1 ? ? 1 ? ? ? ? ? 1 ? ? ? ? 1 ? ? ? ? ? ? ? 1 ? 1 0 1 1 0 1 0 0 0 1 1 2 0 ? ? ? ? 0 ? 1 0 ? ? 2 0 0 0 0 0 1 1 1 0 ? 1 1 0

Yanornis 0 0 0 0 0 0 1 1 0 1 0 0 ? 0 1 ? 1 0 0 ? ? 0 1 1 0 ? ? ? ? ? ? ? 0 1 0 0 0 0 0 0 ? ? ? 1 ? 1 ? ? ? 1 0 1 1 1 0 ? ? ? ? 0 ? 1? 0 1 ? 1 ? ? ? ? ? ? ? ? ? ? ? ? ? ? ? ? ? ? ? ? ? ? ? ? ? ? ? ? ? ? ? ? ? ? ? ? ? ? 0 0 0 0 0 0 ? ? 0 1 0 1 0 0 0 ? 1 ? 1 2 ? ? 0 ? ? ? ? ? 0 1 3 0 0 0 0 2 0 0 2 1 0 0 0 0 ? ? ? 0 1 0 0 ? 0 1 1 1 ? 1 0 1 ? 0 1 1 ? 1 ? 1 0 1 0 0 1 ? 1 0 2 ? 0 ? 0 ? ? 0 ? ? ? ? ? ? ? ? 0 ? ? ? 1 1 ? 1 1 ? ? ? 1 0 0 ? ? ? ? 0 ? 1 3 0 0 1 1 2 1 0 0 0 1 0 3 1 1 0 1 1 0 ? 0 ? 1 2 2 1 1 0 ? 0 0 0 1 ? 0 ? 0 0 0 1 1 1 0 0 1 1 0 ? 0 ? ? ? 1 ? ? 0 0 0 0 1 1 1 1 ? 0 ? ? ? ? ? ? ? ? ? ? ? ? ? ? ? ? ? ? ? ? 0 3 0 0 0 ? ? 1 ? ? 2 1 0 ? ? ? ? 0 ? ? ? 2 ? ? ? ? ? 2 1 1 ? ? ? ? ? 0 1 0 1 0 ? ? 1 1 ? ? ? ? 1 1 1 1 ? 1 0 1 2 0 1 0 0 0 ? 0 ? ? ? ? ? ? 0 1 1 0 0 0 2 0 0 0 0 0 1 ? ? 0 0 0 1 0

Anchiornis 0 0 0 1 0 0 0 0 0 0 0 0 1 1 0 0 1 0 ? 1 0 0 1 1 1 1 1 0 1 1 ? ? 0 0 0 0 0 0 0 ? 0 2 2 0 0 1 1 0 ? 1 0 1 0 1 0 0 0 ? ? ? ? 0? 1 1 ? 1 ? ? 0 0 0 1 ? ? 0 ? ? ? ? ? ? ? ? ? ? ? ? ? ? ? ? ? ? ? ? ? ? ? ? ? ? ? ? ? 0 0 0 0 0 0 0 0 0 1 0 1 0 0 0 0 0 ? 0 0 0 ? 1 ? ? ? 0 ? 0 1 0 0 0 0 0 0 0 0 2 1 0 0 0 1 ? ? ? ? ? ? 0 1 ? 1 0 1 ? 1 0 ? 0 0 1 0 0 1 ? 0 0 1 1 ? 1 ? 1 0 0 1 ? ? ? 1 0 0 1 2 1 1 0 2 ? 0 0 0 ? 1 0 1 1 0 ? ? ? ? 0 0 0 1 0 0 1 0 1 1 1 0 1 0 1 1 1 0 0 0 0 0 2 1 0 0 1 1 0 ? 0 ? 1 2 0 0 1 0 0 0 0 0 0 1 0 0 0 0 0 0 1 0 0 1 0 0 0 1 0 0 0 0 0 2 0 0 0 0 0 0 0 0 0 0 1 0 0 ? 0 0 ? 1 1 0 1 1 0 1 0 ? 0 ? 0 2 ? 0 1 0 0 0 0 1 0 0 0 1 1 0 ? 1 0 0 0 0 0 ? 1 1 0 0 0 1 ? 1 1 ? ? 1 1 ? ? ? 0 1 0 0 ? ? 0 ? ? 0 1 1 ? 0 1 0 1 0 2 0 0 1 0 0 0 0 1 0 0 0 0 ? ? 0 1 0 0 1 0 2 0 0 ? 1 0 1 0 0 1 0 1 1 0

Sinovenator 2 ? 0 1 0 0 0 0 0 0 0 0 1 1 0 0 1 2 0 ? 0 0 1 1 1 1 1 0 1 ? 1 ? 0 ? 0 0 0 0 ? ? ? ? ? ? ? ? ? ? ? ? ? ? ? ? ? ? ? ? ? ? ? 01 ? 1 1 1 0 0 0 0 ? 1 1 1 1 0 1 1 0 2 ? 0 1 0 0 0 1 0 1 0 0 2 1 1 0 ? ? ? ? ? ? ? ? ? ? 0 0 0 0 0 ? ? 0 1 0 ? 0 ? ? ? ? ? ? ? ? ? ? ? ? 0 0 0 0 1 0 0 0 0 0 0 0 0 2 1 0 0 0 1 ? ? ? 0 ? 0 ? ? ? 1 0 1 1 1 0 ? 1 0 ? 0 0 1 ? 0 0 1 0 1 1 1 1 0 0 0 1 0 0 ? ? ? 1 2 1 ? ? ? ? ? ? ? 1 ? ? ? ? ? ? ? ? ? ? ? 0 ? 0 0 1 0 1 1 1 0 1 0 ? ? ? 0 ? ? ? ? ? ? ? ? ? ? ? ? ? ? ? ? ? ? ? ? ? ? ? ? ? ? ? ? ? 0 ? 0 ? ? ? ? ? ? ? ? ? ? ? ? ? ? ? ? 0 0 0 0 0 0 0 0 0 0 ? ? ? ? ? 1 1 ? 1 1 0 1 0 ? 0 1 0 2 1 0 1 0 0 0 0 1 0 0 0 1 1 0 0 1 0 ? 0 0 0 0 1 1 0 0 1 1 1 1 1 ? 1 1 1 ? 0 1 0 1 0 0 ? 0 0 ? ? 0 1 ? 0 0 1 0 1 1 2 0 ? ? ? 0 0 1 0 0 0 0 0 1 1 0 ? ? 0 1 0 0 0 0 1 ? 0 ? ? ? 1 0 ? ? 0

Sinusonasus ? 0 0 1 0 0 0 0 0 0 1 0 1 ? 0 0 1 2 0 1 0 0 1 1 1 1 1 0 1 ? ? ? 0 ? 0 0 ? ? 0 1 0 ? ? ? ? ? ? ? ? ? ? ? ? ? ? ? ? ? ? ? ? ?? ? ? ? ? ? ? ? ? ? ? ? ? ? ? ? ? ? ? ? ? ? ? ? ? ? ? ? ? ? ? ? ? ? ? ? ? ? ? ? ? ? ? ? 0 0 0 0 0 ? ? 0 1 0 ? ? ? ? ? ? ? ? ? 0 ? ? ? ? ? ? ? 0 ? 0 0 0 0 0 0 0 0 1 1 0 0 0 1 1 ? 0 0 ? ? ? ? ? ? ? ? ? ? ? ? ? ? ? ? ? ? ? ? ? ? ? ? ? ? ? ? ? ? ? ? ? 1 0 0 1 2 1 1 1 2 ? 0 0 0 1 1 ? ? ? ? ? ? ? ? ? ? ? ? ? ? ? ? ? ? ? ? ? ? ? ? ? ? ? ? ? ? ? ? ? ? ? ? ? ? ? ? ? ? ? ? ? ? ? ? ? ? ? ? ? ? ? ? ? ? ? ? ? ? ? ? ? ? ? ? ? ? ? ? ? ? ? ? ? ? ? ? ? ? ? ? ? ? ? ? ? ? ? ? ? ? ? ? ? ? ? ? ? 2 ? ? ? ? ? ? ? ? ? ? ? ? ? 0 ? 1 0 ? 0 0 0 ? 1 1 ? 0 1 1 ? 1 1 ? ? 1 1 ? ? ? 0 1 ? ? ? 0 0 ? ? ? 1 1 1 1 1 1 1 ? 2 0 0 1 0 0 0 0 1 1 1 0 0 ? 1 1 1 0 0 1 0 1 1 0 1 1 0 1 ? 0 1 0 1 1 0

Geminiraptor ? ? ? ? ? ? ? ? ? ? ? ? ? ? ? 0 ? ? ? 1 0 0 1 1 1 1 1 1 1 ? 1 0 ? ? ? ? ? ? ? ? ? ? ? ? ? ? ? ? ? ? ? ? ? ? ? ? ? ? ? ? ? ?? ? ? ? ? ? ? ? ? ? ? ? ? ? ? ? ? ? ? ? ? ? ? ? ? ? ? ? ? ? ? ? ? ? ? ? ? ? ? ? ? ? ? ? ? ? ? ? ? ? ? ? ? ? ? ? ? ? ? ? ? ? ? ? ? ? ? ? ? ? ? ? ? ? ? ? ? 0 ? ? ? ? 1 ? ? ? ? ? ? ? ? ? 0 ? ? ? ? ? ? ? ? ? ? ? ? ? ? ? ? ? ? ? ? ? ? ? ? ? ? ? ? ? ? ? ? ? ? ? ? ? ? ? ? ? ? ? ? ? ? ? ? ? ? ? ? ? ? ? ? ? ? ? ? ? ? ? ? ? ? ? ? ? ? ? ? ? ? ? ? ? ? ? ? ? ? ? ? ? ? ? ? ? ? ? ? ? ? ? ? ? ? ? ? ? ? ? ? ? ? ? ? ? ? ? ? ? ? ? ? ? ? ? ? ? ? ? ? ? ? ? ? ? ? ? ? ? ? ? ? ? ? ? ? ? ? ? ? ? ? ? ? ? ? ? ? ? ? ? ? ? ? ? ? ? ? ? ? ? ? ? ? ? ? ? ? ? ? ? ? ? ? ? ? ? ? ? ? ? ? ? ? ? ? ? ? ? ? ? ? ? ? ? ? ? ? ? ? ? ? ? ? ? ? ? ? ? ? ? ? ? ? ? ? ? ? ? ? ? ? ? ? ? ? ? ? ? ? ? ? ? ? ?

Byronosaurus ? 0 0 1 0 0 0 0 0 0 1 0 1 1 1 0 2 2 0 1 0 0 2 1 1 ? 1 1 1 ? 1 0 0 ? 0 0 0 ? 0 1 0 2 2 ? ? ? ? ? ? ? ? ? ? 1 0 ? ? ? ? ? ? ?? ? ? ? ? 0 ? ? ? ? ? ? ? 1 0 1 1 1 ? ? ? 1 1 0 1 ? 0 1 0 0 0 1 1 ? 0 ? ? ? ? ? ? ? ? 0 0 0 0 0 0 0 0 0 1 0 1 0 0 0 ? ? ? ? ? ? ? 1 ? ? ? ? ? 0 1 0 0 0 0 0 0 0 0 2 1 0 ? 0 1 ? 1 ? 0 0 1 ? ? 0 ? ? ? ? ? ? ? ? ? ? ? 0 ? 1 0 0 ? ? 1 ? 1 ? ? ? ? ? ? ? ? ? 0 ? 2 ? ? 1 ? ? ? ? ? ? ? ? ? ? ? ? ? ? ? ? ? ? ? ? ? ? ? ? ? ? ? ? ? ? ? ? ? ? ? ? ? ? ? ? ? ? ? ? ? ? ? ? ? ? ? ? ? ? ? ? ? ? ? ? ? ? ? ? ? ? ? ? ? ? ? ? ? ? ? ? ? ? ? ? ? ? ? ? ? ? ? ? ? ? ? ? ? ? ? ? ? ? ? ? ? ? ? ? ? ? ? ? ? ? ? ? ? ? ? ? ? ? ? ? ? ? ? ? ? ? ? ? ? ? ? ? ? ? ? ? ? ? ? ? ? ? ? 1 ? ? ? 0 ? ? 0 ? 0 ? 0 ? ? ? ? ? ? ? ? ? ? ? ? ? ? ? ? ? ? ? ? ? ? ? ? ? ? ? ? ? ? ? ? 1 0 1 ? ? ? ? ? ? ? ? ? ?

Mei 2 0 0 1 0 0 0 0 0 0 1 0 1 1 ? ? 2 2 0 ? ? 0 2 ? ? ? ? ? ? 1 ? ? 0 ? 0 0 0 0 0 1 0 2 2 0 0 ? ? ? ? 1 0 1 0 1 0 0 ? ? 0 ? ? 01 ? 1 1 1 1 0 0 0 0 1 1 ? ? ? ? ? ? ? ? ? ? ? ? ? ? ? 1 1 0 ? ? ? ? ? ? ? ? ? ? ? ? ? 0 0 0 0 0 ? ? 0 0 1 0 ? ? ? ? 0 ? ? ? ? 0 ? 1 ? 0 0 0 0 0 ? 0 ? 0 ? 0 3 0 0 2 1 0 ? ? ? ? ? ? 0 ? ? 0 1 1 1 0 1 1 1 0 1 ? 0 1 0 0 1 1 0 1 1 0 0 1 1 1 0 0 1 ? ? ? ? 0 0 1 2 1 1 1 2 ? 0 0 0 1 1 ? ? ? 0 ? ? ? ? 0 0 0 1 0 0 1 0 ? 1 1 0 1 0 1 1 1 0 0 0 ? 0 1 1 0 0 ? ? 0 1 ? ? 1 ? 0 0 1 0 ? ? 0 0 ? ? ? ? 0 0 0 0 ? 0 0 ? 0 ? 0 0 0 0 0 0 0 0 0 0 0 0 0 0 0 0 0 0 1 0 ? ? ? ? ? ? ? ? ? 1 0 1 ? ? 0 1 0 2 1 ? 1 0 0 ? 0 1 0 ? ? ? ? 0 ? 1 ? ? ? ? ? ? 1 1 0 ? 1 1 0 1 1 0 1 1 1 0 0 1 ? 1 0 ? ? ? 0 1 ? 0 1 1 ? 1 1 1 1 1 2 0 0 1 0 0 1 0 1 ? ? 0 0 1 1 1 1 0 0 1 0 1 1 0 1 1 0 1 0 0 1 ? 1 1 0

Sinornithoides 2 0 0 1 0 0 0 0 0 0 ? ? ? ? ? 0 2 2 0 1 0 0 2 1 1 1 1 1 1 1 ? ? 0 ? ? ? ? 0 0 1 ? 2 2 0 0 1 1 ? ? 1 0 1 0 ? 0 ? ? ? ? ? ? ?? ? 1 ? 1 ? 0 0 ? ? ? ? ? ? ? ? ? ? ? ? ? ? ? ? ? ? ? ? ? ? ? ? ? ? ? ? ? ? ? ? ? ? ? 0 0 0 0 0 0 0 0 0 1 0 1 0 ? ? ? ? 0 0 0 0 ? 1 ? 0 ? 0 0 0 1 0 0 0 0 0 3 0 0 1 1 0 0 0 1 1 ? 0 0 0 ? ? ? ? ? ? ? ? ? 0 1 0 0 1 1 0 ? 1 ? ? ? ? ? ? ? ? ? ? ? ? ? 0 ? 0 0 1 2 1 1 1 2 ? 0 0 0 1 1 ? ? 1 0 ? ? ? ? ? ? ? ? 0 0 1 0 ? 0 1 0 1 0 ? 1 1 0 0 0 0 0 1 1 0 0 1 1 0 ? 0 ? 1 2 0 0 1 0 0 0 0 0 0 ? 0 0 0 0 0 0 1 0 0 1 0 0 0 0 0 0 0 0 0 0 0 0 0 0 0 0 0 0 0 0 0 0 ? ? ? ? ? ? ? ? ? ? 0 1 ? 1 0 ? 0 2 1 0 1 0 0 0 0 1 0 ? ? ? ? 0 0 1 0 ? 0 0 ? ? 1 1 0 ? 1 1 ? ? 1 ? 1 1 1 0 0 1 0 1 ? ? 0 0 0 ? ? 0 1 1 1 1 1 1 1 1 2 0 0 1 0 0 1 0 1 2 ? 0 0 ? 1 1 1 0 0 1 0 1 1 0 1 1 0 1 0 0 1 0 1 1 0

Xixiasaurus ? ? 0 1 1 1 0 0 0 0 2 0 0 1 1 0 ? 2 1 1 0 0 2 1 1 1 1 1 0 1 1 0 0 0 0 0 0 ? 0 1 0 2 ? ? ? ? ? ? ? ? ? ? ? ? ? ? ? ? ? ? ? ?? ? ? ? 1 ? ? ? ? ? ? ? ? ? ? ? ? ? ? ? ? ? ? ? ? ? ? ? ? ? ? ? ? ? ? ? ? ? ? ? ? ? ? ? 1 0 0 0 ? ? ? 0 1 ? ? ? ? ? ? ? ? ? ? ? ? ? ? ? ? ? ? 0 1 0 0 0 0 0 3 ? 0 2 1 0 0 0 ? ? ? ? 0 ? 1 ? ? ? ? ? ? ? ? ? ? ? ? ? ? ? ? ? ? ? ? ? ? ? ? ? ? ? ? ? ? ? ? ? ? ? ? ? ? ? ? ? ? ? ? ? ? ? ? ? ? ? ? ? ? ? ? ? ? ? ? ? ? ? ? ? ? ? ? ? ? ? ? ? ? ? ? ? ? 0 0 ? ? ? ? ? ? ? ? ? ? ? ? ? ? 0 0 ? ? 0 0 0 ? ? 0 ? ? ? ? ? ? ? ? ? ? ? ? ? ? ? 0 0 0 ? 0 ? 0 ? 0 ? ? ? ? ? ? ? ? ? ? ? ? ? ? ? ? ? ? ? ? ? ? ? ? ? ? ? ? ? ? ? ? ? ? ? ? ? ? ? ? ? ? ? ? ? ? ? ? ? ? ? ? ? ? ? ? ? ? ? ? ? ? ? ? ? ? ? ? ? ? ? ? ? ? ? ? ? ? ? ? ? ? ? ? ? ? ? ? ? ? ? ? ? ? ? ? ? ? ? ? ? ? ? ? ? ? ? ? ? ? ?

IGM_100_44 ? ? ? ? ? ? ? ? ? ? ? ? ? ? ? ? ? ? ? ? ? ? ? ? ? ? ? ? ? ? ? ? ? ? ? ? ? ? ? ? ? ? ? ? ? ? ? ? ? ? ? ? ? ? ? ? ? ? ? ? ? ?1 ? ? ? ? ? ? ? ? ? ? ? ? ? 0 ? ? 1 2 ? ? 1 ? ? ? ? ? ? ? ? ? 1 ? 0 ? ? ? ? ? ? ? ? ? 0 ? ? ? ? ? ? ? ? 1 ? ? ? 0 0 0 ? ? ? ? ? ? 1 ? ? ? ? 0 ? ? ? ? 0 ? ? 3 0 0 1 1 ? ? ? ? 0 ? ? ? ? ? ? ? 0 ? ? ? ? ? ? ? ? ? ? ? ? ? ? ? ? ? ? ? ? ? ? ? ? ? ? ? ? ? ? ? ? ? ? ? ? ? ? ? ? ? ? ? ? ? ? ? ? ? ? ? ? ? ? ? ? ? ? ? ? ? ? ? ? ? ? ? ? ? ? ? ? ? ? ? ? ? ? ? ? ? ? ? 1 2 0 0 1 0 0 ? 0 0 ? 1 0 0 0 0 ? ? 1 0 ? ? 0 0 0 ? ? ? ? 0 ? ? ? 0 0 0 0 0 0 0 0 0 0 ? ? ? ? ? ? ? ? ? ? ? ? ? ? ? ? ? ? ? ? ? ? ? ? ? ? ? ? ? ? ? ? ? ? ? ? ? ? ? ? ? ? ? ? ? ? ? ? ? ? ? ? ? ? ? ? ? ? ? ? ? ? ? ? ? ? ? ? ? ? ? ? ? 1 ? ? ? 0 ? 0 ? 1 0 1 ? ? ? 0 ? ? 1 ? ? 0 1 0 1 1 0 1 1 0 ? 0 ? 2 0 0 0 0

Borogovia ? ? ? ? ? ? ? ? ? ? ? ? ? ? ? ? ? ? ? ? ? ? ? ? ? ? ? ? ? ? ? ? ? ? ? ? ? ? ? ? ? ? ? ? ? ? ? ? ? ? ? ? ? ? ? ? ? ? ? ? ? ?? ? ? ? ? ? ? ? ? ? ? ? ? ? ? ? ? ? ? ? ? ? ? ? ? ? ? ? ? ? ? ? ? ? ? ? ? ? ? ? ? ? ? ? ? ? ? ? ? ? ? ? ? ? ? ? ? ? ? ? ? ? ? ? ? ? ? ? ? ? ? ? ? ? ? ? ? ? ? ? ? ? ? ? ? ? ? ? ? ? ? ? ? ? ? ? ? ? ? ? ? ? ? ? ? ? ? ? ? ? ? ? ? ? ? ? ? ? ? ? ? ? ? ? ? ? ? ? ? ? ? ? ? ? ? ? ? ? ? ? ? ? ? ? ? ? ? ? ? ? ? ? ? ? ? ? ? ? ? ? ? ? ? ? ? ? ? ? ? ? ? ? ? ? ? ? ? ? ? ? ? ? ? ? ? ? ? ? ? ? ? ? ? ? ? ? ? ? ? ? ? ? ? ? ? ? ? ? ? ? ? ? ? ? ? ? ? ? ? ? ? ? ? ? ? ? ? ? ? ? ? ? ? ? ? ? ? ? ? ? ? ? ? ? ? ? ? ? ? ? ? ? ? ? ? ? ? ? ? ? ? ? ? ? ? ? ? ? ? ? ? ? ? ? ? ? ? ? ? ? ? ? ? ? ? 0 ? ? 0 1 1 0 ? ? ? 1 ? ? ? ? ? ? ? 0 0 0 2 ? 0 ? ? ? 1 ? ? 0 1 0 1 1 1 1 ? ? 1 ? ? 2 0 0 0 0

Troodon ? ? ? ? ? 1 ? ? ? ? ? ? ? ? 1 0 ? 2 1 ? 0 0 2 1 1 1 1 1 0 ? ? 0 0 ? ? ? 0 ? 0 1 0 2 2 ? ? ? 1 ? ? ? ? ? ? ? ? ? ? 0 ? 0 ? ?1 ? ? 1 1 0 0 0 0 0 2 1 1 1 1 0 0 1 2 ? 1 0 1 0 1 0 0 1 0 0 0 0 0 1 0 ? ? ? ? ? ? 0 ? ? 1 0 0 0 0 ? ? 0 1 0 ? ? ? ? ? ? ? ? ? ? ? ? ? ? ? ? ? ? 0 0 ? 0 ? 0 3 0 0 1 1 0 0 0 1 0 1 1 0 0 1 ? ? ? 1 1 ? 1 1 0 1 0 0 1 ? 0 1 1 0 0 1 ? 1 1 1 1 1 1 ? 1 0 0 ? ? 0 1 2 ? ? 1 2 ? ? 0 ? 1 ? ? ? ? ? ? ? ? ? ? ? ? ? ? 0 ? ? ? ? ? ? ? ? ? ? ? 0 ? ? ? ? ? 1 0 0 ? 1 0 ? ? ? ? 2 ? 0 1 ? 0 ? 0 ? ? ? ? ? ? ? ? ? ? ? ? ? ? ? ? ? ? ? ? ? ? ? ? ? ? ? ? ? ? ? ? ? ? ? ? ? ? ? ? ? ? ? ? 1 ? ? ? ? ? ? ? ? ? ? 1 ? 0 ? ? ? ? ? ? ? ? ? ? ? ? ? ? ? 0 ? ? ? ? ? ? ? ? ? ? 0 1 ? 1 ? 0 ? ? 1 0 ? ? ? ? ? ? 0 1 ? 0 1 ? 1 1 1 1 0 0 1 ? 0 1 0 1 2 1 0 0 1 1 1 ? 0 0 1 0 1 1 1 1 1 0 1 0 0 2 0 0 0 0

Saurornithoides ? 0 0 1 1 1 0 0 0 0 2 0 0 1 1 0 2 2 1 1 0 0 2 1 1 0 1 1 0 1 1 0 0 0 0 0 0 0 ? ? ? 2 ? 0 0 ? ? ? ? 1 0 1 0 1 0 ? ? ? ? ? ? ?? ? 1 1 1 ? ? ? ? ? ? ? ? 1 ? ? ? 1 ? ? ? ? 1 0 1 ? ? ? ? ? 0 ? ? ? 0 0 1 0 0 1 ? 0 ? 0 1 0 0 0 0 ? ? 0 1 0 ? ? ? ? ? ? ? ? ? 0 ? 1 ? ? ? ? ? 0 0 0 0 0 0 0 3 0 0 1 1 0 0 0 1 0 ? 1 0 0 1 ? ? ? ? ? ? ? ? ? ? ? ? ? ? 0 1 ? 0 0 ? ? ? 1 ? ? ? 1 ? 1 0 0 ? 0 0 1 ? ? ? ? ? ? 0 0 0 ? ? ? ? ? ? ? ? ? ? ? ? ? ? ? ? ? ? ? ? ? ? ? ? ? ? ? ? ? ? ? ? ? ? ? ? ? ? ? ? ? ? ? ? ? ? ? ? ? ? ? ? ? ? ? ? ? ? ? ? ? ? ? ? ? ? ? ? ? ? ? ? ? ? ? ? ? ? ? ? ? ? ? ? ? ? ? ? ? ? ? ? ? ? ? ? ? ? ? ? 0 ? ? ? ? 0 1 0 0 ? 0 1 ? ? ? ? ? 0 0 1 0 ? 0 0 0 0 1 1 0 0 1 1 0 1 ? ? 1 1 1 1 0 1 0 1 ? ? ? ? ? ? ? ? ? ? ? ? ? ? 1 ? ? 0 0 1 0 0 ? ? 1 2 1 ? 0 ? ? 1 ? 0 0 1 0 1 1 0 1 ? 0 1 0 0 2 0 ? ? 0

Zanabazar ? 0 0 1 1 1 0 0 0 0 2 0 0 1 1 0 2 2 1 1 0 0 2 1 1 0 1 1 0 1 1 ? 0 0 0 0 0 0 0 1 0 2 2 0 0 1 1 0 1 1 0 1 0 1 0 0 ? ? 0 0 0 ?? ? 1 1 1 0 ? 0 0 0 2 1 1 1 ? 0 ? 1 2 ? 1 1 1 0 1 0 0 1 0 0 0 0 1 1 0 ? ? ? 0 ? ? ? ? ? 1 0 0 0 0 ? 0 0 1 0 ? ? ? ? ? ? ? ? ? ? ? 1 ? ? ? ? ? 0 0 0 0 0 0 0 3 0 0 1 1 0 0 0 1 0 ? 1 0 0 1 ? ? ? ? ? ? ? ? ? ? ? ? ? ? ? ? ? ? ? ? ? ? ? ? ? ? 1 ? 1 0 0 ? ? 0 1 2 ? ? 1 2 ? 0 0 ? 1 1 ? ? ? ? ? ? ? ? ? ? ? ? ? ? ? ? ? ? ? ? ? ? ? ? ? ? ? ? ? ? ? ? ? ? ? ? ? ? ? ? ? ? ? ? ? ? ? ? ? ? ? ? ? ? ? ? ? ? ? ? ? ? ? ? ? ? ? ? ? ? ? ? ? ? ? ? ? ? ? ? ? ? ? ? ? ? ? ? ? ? ? ? ? ? ? ? ? ? ? ? ? ? ? ? ? ? ? 0 ? ? ? ? ? ? ? ? ? ? ? ? ? ? ? ? ? ? ? ? ? ? ? ? ? ? ? ? ? ? ? ? ? ? ? ? ? ? ? ? ? 0 1 1 0 ? ? ? 1 ? ? ? ? ? ? ? ? ? ? ? 1 0 ? ? ? 1 ? ? ? ? ? ? ? ? ? ? ? ? ? ? ? ? ? ? ?

Xiaotingia 2 0 0 4 ? 0 0 0 0 0 0 0 ? 1 ? 0 1 0 ? 1 0 0 ? 1 1 1 1 0 1 1 ? ? 0 0 0 ? 0 0 0 1 0 2 2 0 ? ? ? ? ? ? ? ? 0 ? 0 0 ? ? ? ? ? 0? ? 1 ? 1 ? ? 0 ? 0 ? ? ? ? ? ? ? ? ? ? ? ? ? ? ? ? ? ? ? ? ? ? ? ? ? ? ? ? ? ? ? ? ? 0 0 0 0 0 0 ? ? 0 1 0 1 0 0 1 0 ? ? 0 0 0 ? ? ? 0 0 0 0 ? ? ? ? ? ? 0 2 0 0 2 1 0 0 0 0 ? ? ? 0 ? ? 0 ? ? 1 ? ? ? ? ? ? ? ? ? 0 ? ? ? 1 ? ? ? ? ? ? 1 ? 0 ? ? ? 0 ? ? 0 1 ? ? ? ? ? ? ? ? ? ? ? 0 1 ? ? ? ? ? ? 0 0 0 1 0 0 1 0 1 1 1 ? ? 0 1 1 1 0 0 0 ? 0 2 1 1 0 1 1 ? ? 0 ? 1 2 0 0 ? 0 0 0 0 ? 0 ? 0 0 0 0 0 0 1 0 0 1 0 0 0 1 0 0 0 0 0 2 0 0 0 0 0 1 1 0 0 1 1 0 0 ? 0 ? 0 1 ? 1 1 1 0 1 0 1 0 ? 0 ? ? ? ? ? ? ? ? ? ? ? ? ? 1 0 0 1 0 0 0 0 0 0 1 1 0 0 ? ? ? ? 1 ? 1 1 1 ? 0 ? 0 1 ? ? ? ? ? ? ? ? ? ? ? ? ? 0 1 0 ? ? 0 1 0 0 0 ? 1 ? ? ? 0 ? ? 0 ? 0 0 0 0 0 0 0 1 1 0 1 0 1 1 0 1 1 0

Mahakala ? ? ? ? ? ? ? ? ? ? ? ? ? ? ? ? ? ? ? ? ? ? ? ? ? ? ? ? ? ? ? ? ? ? ? ? 0 ? ? ? ? ? ? ? ? ? ? ? ? ? ? ? ? ? ? ? ? ? ? ? ? ?? ? ? 1 1 1 0 0 0 ? 1 1 ? ? ? 1 0 ? ? ? ? ? ? ? ? ? ? 0 0 1 1 1 0 0 ? ? ? ? ? ? ? ? ? ? ? ? ? ? ? ? ? ? ? ? ? ? ? ? ? ? ? ? ? ? ? ? ? ? ? ? ? ? ? ? ? ? ? ? ? 0 ? ? ? ? ? ? ? ? ? ? ? ? ? ? ? 0 ? ? 1 ? 0 0 ? 0 ? ? ? ? ? ? ? ? ? ? ? ? ? ? ? 1 1 1 0 0 ? ? 0 1 2 0 0 0 2 ? ? ? ? 0 1 ? ? ? ? ? ? ? ? ? ? ? ? ? 0 ? ? ? ? ? ? ? ? ? ? ? ? ? ? ? ? 1 1 ? ? ? 1 ? 1 ? 1 1 2 0 0 ? 0 ? ? ? ? ? ? ? ? ? ? ? ? ? ? ? ? ? ? ? ? ? ? ? 0 ? ? ? ? ? ? ? ? ? ? ? ? ? ? ? ? 0 0 1 1 2 ? 1 1 0 1 0 1 0 1 0 2 1 ? ? ? ? ? ? ? ? ? ? ? ? ? ? ? ? ? ? ? ? ? ? ? ? ? ? ? ? ? 1 ? 1 1 1 1 1 1 ? 1 0 0 ? 0 ? ? ? 0 1 ? 0 0 1 ? 1 0 1 0 0 ? 0 0 0 1 1 0 ? 0 ? ? 0 0 ? ? ? ? ? ? 1 0 1 ? 0 ? ? ? 1 0 ? ? 0

Austroraptor 3 0 1 ? 0 ? ? ? ? ? ? ? ? ? ? 0 1 ? ? 1 0 0 1 1 0 1 1 ? ? ? ? ? ? ? ? ? 0 0 0 1 0 2 2 0 0 1 1 ? ? ? ? ? ? ? ? ? ? ? ? ? ? ?? ? ? 1 1 0 0 0 0 ? ? ? ? ? ? ? ? ? ? ? ? ? ? ? ? ? ? ? ? ? ? ? ? ? ? ? ? ? ? ? ? ? ? ? 0 0 0 0 0 0 0 0 1 0 ? ? ? ? ? ? ? ? ? ? ? ? ? ? ? ? ? ? ? ? ? ? ? 0 2 0 0 2 1 0 0 0 0 ? ? ? 1 ? 0 ? ? ? 1 0 1 1 0 0 1 0 0 ? 0 0 1 1 ? ? ? ? 1 1 1 1 1 ? ? ? ? ? ? ? ? ? ? ? ? ? ? ? ? ? ? ? ? ? ? ? ? ? ? ? ? ? ? ? ? ? ? ? ? ? ? ? ? ? ? ? 0 1 0 ? 0 0 0 ? ? ? ? ? ? ? ? ? ? ? ? ? ? ? ? ? ? ? ? ? ? ? ? ? ? ? ? ? ? ? ? ? ? ? ? ? ? ? ? ? ? ? ? ? ? ? ? ? ? 0 ? ? ? ? ? ? ? ? ? ? ? ? ? ? ? ? ? ? ? ? ? ? ? ? ? ? ? ? ? ? ? ? ? ? ? ? ? ? ? ? ? ? ? ? ? ? ? ? ? ? ? 1 ? 1 1 ? ? 0 ? 0 1 ? ? ? ? ? ? ? 0 ? ? ? ? 1 ? ? ? ? 0 ? ? ? ? ? ? ? ? ? ? ? ? ? ? ? ? ? ? ? ? 1 0 1 ? ? ? ? ? ? ? ? ? ?

Buitreraptor 3 0 1 ? 0 ? ? ? ? ? ? ? ? ? ? ? 1 ? ? 1 ? 0 1 1 0 ? 1 0 1 ? ? ? 0 0 0 0 0 0 ? ? ? ? ? 0 0 1 1 0 ? 1 0 1 0 ? 0 0 ? ? ? ? ? 01 1 1 ? 1 ? 0 0 0 0 ? 0 ? ? ? ? ? ? ? ? ? ? ? ? ? ? ? ? ? ? ? ? ? 0 ? ? ? ? ? ? ? ? ? 0 0 0 ? 0 0 ? ? 0 1 0 1 ? ? ? ? ? ? ? ? 0 ? ? ? ? ? ? ? ? 1 ? ? ? ? 0 0 0 0 2 2 0 ? 0 0 ? ? ? 1 ? 0 0 1 0 1 0 1 1 0 0 1 0 0 1 1 0 1 1 0 1 1 0 1 1 1 0 0 ? ? 1 0 0 ? 0 0 1 2 1 1 0 2 ? ? 0 0 1 1 ? ? ? ? ? ? ? ? 0 0 0 1 0 0 1 0 ? 1 1 0 1 0 1 1 1 0 0 0 0 0 2 1 0 0 ? 1 0 1 ? ? 1 ? 0 0 ? ? ? ? ? ? ? ? ? ? ? ? ? ? ? ? ? ? ? ? ? ? ? ? ? 0 ? ? ? ? ? ? ? ? ? ? ? ? ? ? 0 ? ? ? 1 ? ? ? 1 1 0 1 1 1 ? ? 0 2 1 0 ? ? 0 ? 0 1 ? ? ? 1 1 0 0 1 2 ? 2 1 0 0 1 1 ? 1 0 1 1 1 1 ? ? ? 1 ? 0 1 0 1 0 0 0 0 0 ? ? 0 1 1 0 ? 1 0 1 0 2 0 0 ? 0 0 0 1 1 0 0 0 0 ? 1 0 ? ? ? ? ? 0 0 0 1 ? ? ? 0 1 1 ? ? ? 0

Rahonavis ? ? ? ? ? ? ? ? ? ? ? ? ? ? ? ? ? ? ? ? ? ? ? ? ? ? ? ? ? ? ? ? ? ? ? ? ? ? ? ? ? ? ? ? ? ? ? ? ? ? ? ? ? ? ? ? ? ? ? ? ? ?? ? ? ? ? ? ? ? ? ? ? ? ? ? ? ? ? ? ? ? ? ? ? ? ? ? ? ? ? ? ? ? ? ? ? ? ? ? ? ? ? ? ? ? ? ? ? ? ? ? ? ? ? ? ? ? ? ? ? ? ? ? ? ? ? ? ? ? ? ? ? ? ? ? ? ? ? ? ? ? ? ? ? ? ? ? ? ? ? ? ? ? ? ? ? ? ? ? ? ? ? ? ? ? ? ? ? 0 1 1 1 1 1 ? 1 1 1 ? 0 1 ? 1 1 ? ? 0 0 1 2 1 1 0 2 ? 0 0 0 1 1 ? ? ? ? ? ? ? ? ? ? 0 1 0 0 1 ? ? ? ? ? ? ? 1 ? ? ? ? ? ? ? 3 1 0 0 ? 1 0 1 0 ? ? ? ? ? ? ? ? ? ? ? ? ? ? ? ? ? ? ? ? ? ? ? ? ? ? ? ? ? ? ? ? ? ? ? ? ? ? ? ? ? ? ? ? ? 0 1 0 0 1 1 1 1 1 1 0 1 1 1 0 1 0 2 1 0 1 0 0 0 0 ? 0 0 0 ? 1 0 0 1 1 ? 2 1 ? 0 1 1 ? 1 ? ? ? 1 1 1 1 1 1 ? 0 ? 0 1 0 0 ? ? 0 ? ? ? 1 1 0 0 1 0 1 0 1 0 0 1 0 0 0 1 1 0 0 0 0 1 1 0 ? 0 1 1 0 0 0 0 2 1 0 ? ? 1 1 0 ? ? 0

Unenlagia ? ? ? ? ? ? ? ? ? ? ? ? ? ? ? ? ? ? ? ? ? ? ? ? ? ? ? ? ? ? ? ? ? ? ? ? ? ? ? ? ? ? ? ? ? ? ? ? ? ? ? ? ? ? ? ? ? ? ? ? ? ?? ? ? ? ? ? ? ? ? ? ? ? ? ? ? ? ? ? ? ? ? ? ? ? ? ? ? ? ? ? ? ? ? ? ? ? ? ? ? ? ? ? ? ? ? ? ? ? ? ? ? ? ? ? ? ? ? ? ? ? ? ? ? ? ? ? ? ? ? ? ? ? ? ? ? ? ? ? ? ? ? ? ? ? ? ? ? ? ? ? ? ? ? ? ? ? ? ? ? ? ? ? ? ? ? ? ? ? 1 ? 1 1 0 0 1 1 1 0 0 ? ? ? ? ? ? ? ? ? ? ? ? ? ? ? ? 0 0 ? ? ? ? ? ? ? ? ? ? ? ? 0 1 0 0 1 0 ? ? ? ? ? ? 1 1 1 0 0 0 0 0 ? ? ? ? ? ? ? ? ? ? ? ? ? ? ? ? ? ? ? ? ? ? ? ? ? ? ? ? ? ? ? ? ? ? ? ? ? ? ? ? ? ? ? 0 ? ? ? 1 ? 0 ? ? ? ? 0 2 0 0 1 1 1 1 1 1 0 1 1 1 0 ? 0 2 1 0 1 0 0 0 0 1 0 1 0 1 ? 0 0 1 2 0 ? 1 ? 0 1 0 0 ? ? ? ? ? 1 1 1 1 ? 1 0 1 0 1 0 0 ? 0 ? ? ? ? ? ? 0 ? ? ? ? ? ? ? ? ? ? ? ? ? ? ? ? ? ? ? ? ? ? ? ? 1 0 ? ? ? ? ? ? ? ? ? ? ? ? ? ?

Shanag ? ? ? ? ? ? ? ? ? ? ? ? ? ? 1 0 1 0 ? 1 0 ? 1 0 0 ? 1 3 1 ? 0 ? ? ? ? ? ? ? ? ? ? ? ? ? ? ? ? ? ? ? ? ? ? ? ? ? ? ? ? ? ? ?? ? ? ? ? ? ? ? ? ? ? ? ? ? ? ? ? ? ? ? ? ? ? ? ? ? ? ? ? ? ? ? ? ? ? ? ? ? ? ? ? ? ? ? 0 0 0 0 0 ? ? ? 0 ? ? ? ? ? ? ? ? ? ? ? ? ? ? ? ? ? ? ? ? ? ? ? ? 0 0 0 0 1 0 0 0 0 0 1 ? ? 2 0 0 ? ? ? ? ? ? ? ? ? ? ? ? ? ? ? ? ? ? ? ? ? ? ? ? ? ? ? ? ? ? ? ? ? ? ? ? ? ? ? ? ? ? ? ? ? ? ? ? ? ? ? ? ? ? ? ? ? ? ? ? ? ? ? ? ? ? ? ? ? ? ? ? ? ? ? ? ? ? ? ? ? ? ? ? ? ? ? ? ? ? ? ? ? ? ? ? ? ? ? ? ? ? ? ? ? ? ? ? ? ? ? ? ? ? ? ? ? ? ? ? ? ? ? ? ? ? ? ? ? ? ? ? ? ? ? ? ? ? ? ? ? ? ? ? ? ? ? ? ? ? ? ? ? ? ? ? ? ? ? ? ? ? ? ? ? ? ? ? ? ? ? ? ? ? ? ? ? ? ? ? ? ? ? ? ? ? ? ? ? ? ? ? ? ? ? ? ? ? ? ? ? ? ? ? ? ? ? ? ? ? ? ? ? ? ? ? ? ? ? ? ? ? ? ? ? ? ? ? ? ? ? ? ? ? ? ? ? ? ?

Tianyuraptor 0 0 0 ? 0 ? ? ? 0 0 0 0 ? ? ? ? 1 0 ? ? 0 1 1 1 0 1 1 3 1 ? ? ? 0 0 0 0 0 ? ? ? ? ? ? 0 0 1 1 ? ? ? ? 1 ? ? 0 ? ? ? ? 0 ? ?? ? ? 1 1 ? ? 0 ? 0 ? 1 ? ? ? ? ? ? ? ? ? ? ? ? ? ? ? ? ? ? ? ? ? ? ? ? ? ? ? ? ? ? ? 0 ? ? ? ? ? ? ? ? ? ? ? ? ? ? ? ? ? ? ? ? ? ? ? ? ? ? ? 0 0 ? 0 0 0 0 0 0 0 0 0 0 ? 0 0 1 1 0 1 ? ? ? ? ? ? ? ? ? 0 0 ? ? ? ? 0 ? 1 ? 0 ? 1 0 ? 1 ? ? 0 0 ? 1 ? ? 1 ? 0 ? 2 1 1 0 1 0 ? 0 ? ? 1 ? ? ? 1 ? 1 ? 1 0 0 0 ? 0 0 1 0 ? 1 1 0 ? 0 1 1 1 0 0 0 0 0 1 0 0 0 ? 1 0 1 0 ? 1 2 1 0 1 0 0 0 0 1 0 ? 0 ? 0 0 0 1 1 0 0 1 0 0 0 1 0 0 0 0 0 2 1 0 0 0 0 1 1 0 0 1 1 0 ? 2 1 0 1 1 1 1 1 1 0 1 0 ? 0 1 ? ? ? 0 1 0 0 0 0 ? ? 0 0 1 1 0 ? 1 ? 0 0 ? 0 ? 1 1 ? 1 0 1 1 1 1 ? 1 1 ? ? 0 1 0 1 ? ? ? ? ? ? ? ? ? ? ? 0 1 0 1 0 1 0 ? ? ? ? 0 ? 1 2 0 0 ? ? 1 0 0 ? ? ? ? ? ? ? ? ? 0 1 ? ? ? ? 1 1 0

Hesperonychus ? ? ? ? ? ? ? ? ? ? ? ? ? ? ? ? ? ? ? ? ? ? ? ? ? ? ? ? ? ? ? ? ? ? ? ? ? ? ? ? ? ? ? ? ? ? ? ? ? ? ? ? ? ? ? ? ? ? ? ? ? ?? ? ? ? ? ? ? ? ? ? ? ? ? ? ? ? ? ? ? ? ? ? ? ? ? ? ? ? ? ? ? ? ? ? ? ? ? ? ? ? ? ? ? ? ? ? ? ? ? ? ? ? ? ? ? ? ? ? ? ? ? ? ? ? ? ? ? ? ? ? ? ? ? ? ? ? ? ? ? ? ? ? ? ? ? ? ? ? ? ? ? ? ? ? ? ? ? ? ? ? ? ? ? ? ? ? ? ? ? ? ? ? ? ? ? ? ? ? ? ? ? ? ? ? ? ? ? ? ? ? ? ? ? ? ? ? ? ? ? ? ? ? ? ? ? ? ? ? ? ? ? ? ? ? ? ? ? ? ? ? ? ? ? ? ? ? ? ? ? ? ? ? ? ? ? ? ? ? ? ? ? ? ? ? ? ? ? ? ? ? ? ? ? ? ? ? ? ? ? ? ? ? ? ? ? ? ? ? ? ? ? ? ? ? ? ? ? ? ? ? ? ? ? ? ? ? ? ? 1 ? ? 1 1 0 1 0 1 0 1 0 2 1 0 1 0 3 1 0 1 0 ? 0 3 ? 0 ? ? ? ? ? ? ? ? ? ? ? ? ? ? ? ? ? ? ? ? ? ? ? ? ? ? ? ? ? ? ? ? ? ? ? ? ? ? ? ? ? ? ? ? ? ? ? ? ? ? ? ? ? ? ? ? ? ? ? ? ? 1 0 1 0 0 1 ? ? 1 ? ? ? 0 ? ? 0

Cryptovolans 0 ? 0 ? ? ? ? ? ? ? ? ? ? ? ? ? ? ? ? ? ? ? ? ? ? ? ? ? ? ? ? ? ? ? ? ? ? ? ? ? ? ? ? ? ? ? ? ? ? ? ? ? ? ? ? ? ? ? ? ? ? ?? ? ? ? ? ? ? ? ? ? ? ? ? ? ? ? ? ? ? ? ? ? ? ? ? ? ? ? ? ? ? ? ? ? ? ? ? ? ? ? ? ? ? 0 ? ? ? ? ? ? ? ? ? ? ? ? ? ? ? ? ? ? ? ? ? ? ? ? ? ? ? ? ? ? ? 0 ? 0 0 ? 1 1 0 0 ? ? ? 1 ? 0 1 0 ? ? ? ? ? ? ? ? ? ? ? ? ? ? ? ? 1 ? ? ? ? ? ? ? ? ? ? ? ? ? ? ? ? ? 0 1 2 2 1 ? 1 0 ? 0 ? ? 1 1 ? ? 1 1 1 ? 1 0 0 0 ? 0 ? ? ? 1 1 ? ? ? 0 1 1 1 0 ? 0 ? 0 2 1 1 0 1 1 0 ? 0 ? ? ? ? ? ? 0 ? 0 ? ? 0 ? ? ? 0 0 0 ? 1 1 0 0 1 0 0 0 0 0 0 0 0 0 1 0 0 0 0 0 1 0 0 0 m 0 ? ? ? ? 1 ? ? ? ? ? ? ? ? ? ? ? ? ? ? 0 ? ? 3 ? ? ? ? 0 0 3 ? 0 ? ? ? ? ? ? ? ? ? ? ? ? ? ? ? ? 1 ? ? ? ? ? ? ? ? 1 ? ? ? ? ? ? ? ? ? ? ? ? 1 ? 1 0 2 ? ? ? ? ? 0 ? ? ? ? 0 ? ? ? ? 0 0 ? ? ? ? ? ? ? 1 0 1 ? ? 1 0 1 1 0

Sinornithosaurus 1 0 0 0 1 0 0 0 0 0 0 ? 0 0 1 ? 0 1 0 ? 1 0 1 1 1 0 1 1 3 1 1 ? ? 0 0 0 0 0 0 0 1 0 1 ? 0 0 1 1 ? ? ? ? 1 ? 1 0 ? 1 ? ? ? 0 ?0 1 1 ? 1 1 0 0 1 0 1 1 ? ? ? ? ? ? ? ? 0 ? ? ? ? ? ? ? ? ? ? ? ? ? ? ? ? ? ? ? ? ? ? 0 0 0 0 0 1 0 0 0 0 0 1 0 0 ? 0 0 0 0 0 0 ? 1 ? ? 0 0 0 0 1 2 0 0 1 0 0 0 1 1 0 0 0 0 0 1 1 0 1 0 0 ? ? ? ? 0 1 1 0 0 ? ? 0 ? 0 0 1 ? 0 1 1 0 ? ? ? ? 0 0 ? 1 ? 0 ? ? 0 1 2 ? 1 ? 1 ? ? 0 0 1 1 ? ? ? 1 0 1 ? 1 0 0 0 1 0 0 1 0 1 1 3 0 1 0 1 1 1 0 ? ? ? ? 2 1 1 0 1 1 0 ? 0 ? ? ? ? 0 1 0 0 0 ? ? 0 1 0 0 0 0 0 1 1 1 0 0 1 0 0 0 0 0 0 0 0 0 1 0 0 0 0 0 1 0 0 0 0 0 0 ? 1 0 ? ? 1 0 1 1 0 1 ? 1 0 ? 0 2 ? 0 2 0 3 1 0 1 0 0 0 3 1 0 0 1 3 ? 0 0 2 ? 1 1 0 1 1 1 ? 1 1 ? ? ? ? ? ? ? 0 1 ? ? ? ? ? ? ? 0 1 1 0 0 1 ? 1 0 2 0 0 1 0 0 0 1 1 2 0 0 0 ? 1 0 0 0 1 ? ? 2 0 0 1 1 0 1 0 1 1 0 1 1 0

Graciliraptor ? ? ? ? ? ? ? ? ? ? ? ? ? ? ? ? ? ? ? ? ? ? ? ? ? ? ? ? ? ? ? ? ? ? ? ? ? ? ? ? ? ? ? ? ? ? ? ? ? ? ? ? ? ? ? ? ? ? ? ? ? ?? ? ? ? ? ? ? ? ? ? ? ? ? ? ? ? ? ? ? ? ? ? ? ? ? ? ? ? ? ? ? ? ? ? ? ? ? ? ? ? ? ? ? ? ? ? ? ? ? ? ? ? ? ? ? ? ? ? ? ? ? ? ? ? ? ? ? ? ? ? ? ? ? ? ? ? ? 0 0 ? 1 1 0 ? ? ? ? 1 1 0 1 ? 0 ? ? ? ? ? ? ? ? ? ? ? ? ? ? ? ? ? ? ? ? ? ? ? ? ? ? ? ? ? ? ? ? ? ? ? ? ? ? ? 1 ? ? ? ? 1 1 ? ? ? ? ? ? ? ? ? ? ? ? ? ? ? ? ? ? ? ? ? ? ? ? 1 0 0 0 0 0 ? 1 1 0 ? ? 0 ? 0 ? 1 2 1 0 1 0 0 0 0 0 ? 1 0 0 0 0 0 1 1 1 ? 0 ? 0 0 ? 0 0 0 0 0 ? 1 0 0 0 0 0 1 0 ? 0 0 0 ? ? ? ? ? ? ? ? ? ? ? ? ? ? ? ? ? ? ? ? ? ? ? ? ? ? ? ? ? ? ? ? ? ? ? ? ? ? ? ? ? ? ? ? ? ? ? ? ? ? ? ? ? ? ? ? ? ? ? ? ? 0 0 ? ? 0 1 1 1 ? ? 0 1 ? ? 0 ? ? ? ? 0 1 0 2 ? ? ? ? ? 0 ? ? ? 1 0 2 0 0 1 ? 0 1 ? ? 1 0 1 ? 0

CAGS_“Microraptor_zhaoianus” ? ? ? ? ? ? ? ? ? ? ? ? ? ? ? ? ? ? ? ? ? ? ? ? ? ? ? ? ? ? ? ? ? ? ? ? ? ? ? ? ? ? ? ? ? ? ? ? ? ? ? ? ? ? ? ? ? ? ? ? ? ?? ? ? ? ? ? ? ? ? ? ? ? ? ? ? ? ? ? ? ? ? ? ? ? ? ? ? ? ? ? ? ? ? ? ? ? ? ? ? ? ? ? ? ? 0 0 0 0 1 ? ? 0 0 0 ? ? ? ? ? ? ? ? ? 0 ? ? ? ? ? ? ? ? ? ? ? ? ? ? 0 ? 0 1 0 0 ? ? ? 1 ? 0 0 ? ? ? ? ? 1 0 ? ? 0 0 1 0 0 1 0 0 1 0 0 1 1 0 1 1 ? 1 ? 0 1 ? ? ? 1 ? 0 1 2 2 1 ? 1 0 ? 0 ? 1 1 1 1 ? 1 0 1 ? ? 0 0 0 1 0 0 ? 1 1 1 ? 0 ? 0 1 1 1 0 0 0 0 0 2 1 1 0 1 1 0 ? 0 ? 1 2 1 0 1 0 ? 0 ? ? 0 ? 0 0 0 ? 0 1 0 1 0 0 1 1 0 0 0 ? 0 0 0 0 1 0 0 0 ? 1 1 0 0 1 1 ? 0 0 0 0 1 1 ? 0 1 1 ? 1 0 1 0 ? 0 2 ? 0 2 ? ? 1 0 1 0 0 0 3 1 0 0 1 3 ? 0 0 2 ? 1 1 0 1 1 1 ? 1 1 ? 1 1 1 ? 1 1 0 1 ? ? ? ? 0 ? ? 0 1 1 1 1 1 0 1 0 2 0 0 1 0 0 0 1 1 ? ? 0 0 ? 1 0 0 ? 1 1 0 2 0 0 1 1 0 1 0 1 1 0 1 1 0

Microraptor_zhaoianus 0 0 ? ? ? 0 0 0 0 0 ? ? ? ? ? ? ? ? ? ? ? ? ? ? ? ? ? ? ? ? 1 0 ? ? ? ? ? ? ? ? ? ? ? ? ? ? ? ? ? ? ? ? ? ? ? ? ? ? ? ? ? ?? ? ? ? ? ? ? ? ? ? ? ? ? ? ? ? ? ? ? ? ? ? ? ? ? ? ? ? ? ? ? ? ? ? ? ? ? ? ? ? ? ? ? 0 ? ? ? ? ? ? ? ? ? ? ? ? ? ? ? ? ? ? ? ? ? ? ? ? ? ? ? 0 1 ? ? 0 1 0 0 ? 0 2 0 0 ? ? ? ? ? ? 0 0 ? ? ? ? ? ? ? ? ? ? ? ? ? ? ? ? ? ? ? ? ? ? ? ? ? ? ? ? ? 1 ? ? ? ? 0 ? ? ? ? ? 1 0 ? 0 ? 1 ? 1 ? ? ? ? ? ? ? ? ? ? ? ? ? ? ? ? ? ? ? ? ? ? ? ? ? ? ? ? ? 2 1 1 0 1 1 0 ? 0 ? 1 2 ? 0 ? ? ? ? ? ? ? ? ? ? ? ? ? ? ? ? 0 ? ? ? ? ? ? ? ? ? ? ? ? ? ? ? ? ? ? ? ? ? ? ? ? ? ? ? ? ? ? ? ? 1 ? ? ? ? ? ? ? ? ? ? ? ? ? 1 0 1 ? ? ? ? ? 0 ? ? ? ? ? ? ? ? ? ? ? ? ? ? ? 1 1 ? ? ? ? ? ? ? 0 1 ? ? ? ? ? ? ? ? ? ? ? ? 1 ? 1 ? ? ? 0 1 ? 0 0 ? ? ? ? 0 ? ? 1 0 0 0 ? ? ? 2 0 0 ? 1 0 1 0 1 1 0 1 1 0

Microraptor_gui 0 ? 0 ? ? ? ? ? ? ? ? ? ? ? ? ? ? ? ? ? ? ? ? ? ? ? ? ? ? ? ? ? ? ? ? ? 0 ? ? ? ? ? ? 0 0 1 1 ? ? ? ? 1 ? ? ? ? ? ? ? 0 ? ?? ? 1 ? 1 ? ? ? ? 0 ? ? ? ? ? ? ? ? ? ? ? ? ? ? ? ? ? ? ? ? ? ? ? ? ? ? ? ? ? ? ? ? ? ? ? ? 0 0 1 ? ? ? ? ? ? ? ? ? ? ? ? ? ? ? ? ? ? ? ? ? ? ? ? ? ? ? ? ? 0 0 0 2 0 ? 0 0 0 ? ? ? 0 ? ? 0 ? ? 1 ? ? ? ? ? ? ? ? 1 0 0 1 ? 0 ? 1 0 ? 1 ? ? ? ? ? ? ? ? 1 0 0 1 2 2 1 ? 1 0 0 0 1 ? 1 1 1 ? 1 1 1 1 1 ? ? 0 ? 0 ? ? 1 1 1 ? ? 1 0 1 1 1 0 0 0 0 0 2 1 1 0 1 1 0 ? 0 ? 1 2 1 0 1 0 0 0 ? ? 0 ? 0 0 0 0 0 1 1 1 0 0 1 1 0 0 0 0 0 0 0 0 1 0 0 0 0 1 1 0 0 1 1 0 ? ? ? ? ? ? ? ? ? ? ? ? ? ? 0 ? ? ? ? 0 2 0 3 1 ? ? ? 0 0 3 1 0 ? 1 3 ? 0 0 2 ? 1 1 0 1 1 1 1 1 1 ? 1 1 ? ? 0 ? 0 1 ? ? ? ? ? ? ? ? 1 ? ? 1 1 0 1 0 2 0 0 1 ? 0 0 ? ? 2 0 0 0 ? 1 0 0 ? 1 ? ? 2 0 0 1 1 0 1 ? ? 1 0 1 1 0

Bambiraptor 0 0 0 0 0 0 0 0 0 0 0 0 ? 1 ? 0 0 1 ? 1 0 0 1 1 0 1 1 3 1 ? ? 0 0 ? 0 0 0 0 0 1 0 1 ? 0 0 1 1 0 ? 1 0 1 0 1 0 0 1 ? ? ? ? 00 1 1 1 1 ? ? ? 1 0 1 ? ? ? ? ? ? ? ? ? 0 ? ? 0 ? 1 ? ? ? ? 1 ? ? 0 ? ? ? ? ? ? ? ? ? 0 0 0 0 0 1 ? ? 0 0 ? 1 0 1 1 0 ? ? ? ? 0 ? ? ? 0 0 0 0 0 0 ? ? 0 ? 0 0 0 0 1 0 0 0 0 0 1 1 0 1 ? 0 ? 1 1 2 0 1 1 0 0 ? 0 0 ? 0 0 1 1 1 1 0 0 1 1 1 0 ? 0 ? ? 1 0 1 0 0 1 2 1 1 ? 1 1 0 0 0 1 1 ? ? ? 1 0 ? 1 1 0 0 0 1 0 0 1 0 ? 1 1 0 1 0 1 1 1 0 0 0 0 0 2 1 0 0 ? 1 0 1 0 ? 1 2 1 0 1 0 1 0 0 1 0 1 0 0 0 0 0 0 1 0 0 1 0 0 0 0 0 0 0 0 0 1 1 0 0 0 0 1 1 0 0 0 1 ? 0 ? 1 0 1 1 1 ? 1 1 0 1 0 1 0 ? 0 2 1 0 1 0 0 0 0 1 0 0 0 1 1 0 0 1 2 ? 0 0 0 0 1 1 0 ? 1 1 1 1 1 0 1 1 ? ? 0 1 0 1 0 0 0 0 0 0 0 0 1 1 0 0 1 0 1 0 1 0 0 ? 0 0 0 1 1 0 0 0 0 1 1 0 0 0 1 1 0 0 1 0 1 1 0 1 0 1 1 0 0 1 0

Adasaurus ? ? ? ? ? ? ? ? ? ? ? ? ? ? ? ? ? ? ? ? ? ? ? ? ? ? ? ? ? ? ? ? ? ? ? ? 0 ? 0 1 0 1 ? 0 0 1 1 0 ? 2 0 1 0 ? ? ? ? ? ? ? ? 0? ? 1 ? 1 ? ? ? ? ? 1 ? ? 0 ? ? ? ? ? ? ? ? ? ? ? ? ? 0 ? ? ? ? ? ? ? ? ? ? ? ? ? ? ? ? ? ? 0 ? ? ? ? ? ? ? ? ? ? ? ? ? ? 0 0 0 ? ? ? ? ? 0 0 0 ? ? ? ? ? 0 0 0 ? ? ? ? ? 0 ? ? ? ? ? ? ? 0 ? 1 2 ? 1 ? ? ? 0 ? ? ? 0 0 1 1 1 ? ? 0 1 1 ? ? ? 0 1 1 1 0 ? ? ? ? 2 ? 2 ? 1 ? ? ? ? ? 1 ? ? ? ? ? ? ? ? ? ? 0 ? 0 ? 1 1 ? ? 1 0 ? ? ? ? ? ? ? ? ? ? ? ? ? ? ? ? ? ? ? ? ? ? ? ? ? ? ? ? ? ? ? ? ? ? ? ? ? ? ? ? ? ? ? ? ? ? ? ? ? ? ? ? ? ? ? ? ? ? ? ? ? ? ? ? 0 2 0 0 1 1 1 0 ? 1 0 1 ? 1 0 ? 0 2 1 0 ? ? 0 0 ? ? ? 0 0 1 ? 0 0 1 2 ? ? 0 0 ? ? ? ? ? ? ? ? ? 1 ? 1 1 ? ? 0 ? 0 1 0 ? ? ? ? ? ? ? ? ? ? 1 1 0 1 0 1 0 0 ? 0 0 0 1 1 0 0 0 0 ? ? 1 1 0 1 1 0 0 1 0 1 1 0 1 0 1 ? ? 0 1 0

Tsaagan 0 0 0 0 0 0 0 0 0 0 2 0 0 1 2 0 1 0 ? 1 0 0 0 1 1 1 1 3 0 1 1 0 0 0 0 ? 0 0 0 1 0 1 2 0 0 1 1 0 1 2 0 1 0 1 0 0 1 0 0 1 0 00 1 1 ? 1 1 1 1 1 0 1 0 0 0 1 2 0 0 0 1 0 0 0 0 0 1 1 0 0 1 1 0 1 0 0 ? ? ? 0 1 ? ? ? 0 0 0 0 0 1 0 ? 0 0 0 1 0 1 1 0 0 0 0 0 0 ? 1 1 1 0 0 0 0 0 ? 1 0 ? 0 0 0 0 1 0 0 0 0 0 1 ? 0 1 0 0 0 1 1 2 0 1 1 0 0 ? ? 0 1 0 0 ? ? ? ? ? ? ? ? ? ? ? ? ? ? ? ? ? 0 0 1 2 1 2 ? 1 1 0 0 0 ? ? 1 1 1 1 1 ? ? ? ? ? 0 1 0 0 ? ? 1 ? ? 0 1 0 1 1 1 0 ? 0 ? 0 1 0 ? ? ? ? ? ? ? ? ? ? ? ? 1 ? ? 0 ? ? ? ? ? ? ? ? ? ? ? ? ? ? ? ? ? ? ? ? ? ? ? ? ? 0 0 0 ? ? ? ? ? ? ? ? ? ? ? ? ? ? ? ? ? ? ? ? ? ? ? ? ? ? ? ? ? 1 0 0 0 1 0 ? ? 1 ? 0 ? ? ? ? ? ? ? ? ? ? ? ? ? ? ? ? 1 ? 1 1 1 0 0 1 0 1 ? ? 0 0 0 ? ? ? 1 1 1 0 1 ? 1 0 1 0 0 0 ? 0 0 ? ? 0 0 0 0 ? ? 0 ? 0 ? ? ? ? 1 0 1 ? 0 1 0 0 1 0 0 0 0

Velociraptor 0 0 0 0 0 0 0 0 0 0 2 0 0 1 2 0 1 0 ? 1 0 0 0 1 1 1 1 3 1 1 1 0 0 0 0 0 0 0 0 1 0 1 2 0 0 1 1 0 1 2 0 1 0 1 0 0 1 0 0 1 0 00 1 1 1 1 1 ? 1 1 0 1 0 0 0 1 2 0 0 0 1 0 0 0 0 0 1 1 0 0 1 1 0 0 0 0 0 1 0 0 1 1 0 1 0 0 0 0 0 1 0 0 0 0 0 1 0 1 1 0 0 0 0 0 0 1 1 1 1 0 0 0 0 0 2 1 0 0 0 0 0 0 1 0 0 0 0 0 1 1 0 1 0 0 0 1 1 2 0 1 1 0 0 0 0 ? 1 0 0 1 1 0 1 0 0 1 1 1 0 1 0 1 1 0 0 0 0 0 1 2 1 2 0 1 1 0 0 0 1 1 1 1 1 1 0 1 0 1 0 0 0 1 0 0 1 1 1 1 1 0 1 0 1 1 1 0 0 0 0 0 1 0 0 0 ? 1 0 1 0 1 1 2 0 0 1 0 0 0 0 0 0 1 0 0 0 0 0 0 1 0 0 1 0 0 0 0 0 0 0 0 0 2 0 0 0 0 0 1 1 0 0 0 0 0 0 2 0 0 1 1 1 0 1 1 0 1 0 1 0 1 0 2 1 0 1 1 0 0 0 1 0 0 0 1 1 0 0 1 2 ? 0 0 0 0 1 1 0 0 ? ? 1 1 1 1 1 1 1 1 0 1 0 0 0 0 0 0 0 0 0 0 1 1 0 0 1 0 1 0 1 0 0 0 0 0 0 1 1 0 0 0 0 1 0 0 ? 0 1 1 0 0 1 0 1 1 0 1 0 0 1 0 0 0 0

Deinonychus ? ? 0 0 0 0 0 0 0 0 0 0 0 ? ? 0 0 1 ? 1 0 0 1 1 1 1 1 3 1 ? ? 0 0 ? 0 0 0 0 0 1 0 1 2 0 0 1 1 0 1 2 0 1 0 1 0 0 1 0 0 1 0 ?? ? 1 ? ? ? ? ? ? ? ? ? ? ? ? ? ? ? ? 1 ? 0 ? ? ? ? 1 0 0 ? 1 0 0 ? ? 0 1 0 0 1 1 0 ? 0 0 0 0 0 0 ? ? 0 0 0 ? 0 1 1 0 0 ? 0 0 0 1 1 ? 1 0 0 0 0 0 2 ? 0 ? 0 0 0 0 1 0 0 0 0 0 1 1 0 1 0 0 ? 1 1 2 0 1 1 0 0 0 0 0 1 0 0 0 1 1 1 0 0 1 1 1 0 1 ? ? ? ? 0 0 0 0 1 2 1 2 0 1 1 ? 0 ? 1 1 ? 1 ? ? ? ? ? 1 ? ? ? 1 0 0 1 0 ? 1 1 0 1 0 1 1 1 0 0 0 0 0 1 1 0 0 ? 1 0 1 0 1 1 2 0 0 1 0 0 0 0 0 0 1 0 0 0 0 0 0 1 0 0 1 0 0 0 0 0 0 0 0 0 2 0 0 0 0 0 1 1 0 0 0 1 0 0 2 1 0 1 1 1 0 1 1 0 1 0 1 0 1 0 2 1 0 ? 0 0 0 0 1 0 0 0 1 1 0 ? 1 2 0 0 0 0 0 1 1 0 0 0 1 1 1 1 1 1 1 1 1 0 1 0 1 0 0 0 0 0 0 0 0 1 1 0 0 0 0 1 0 0 0 0 0 0 0 0 1 1 0 0 0 0 1 0 0 1 0 1 1 1 0 1 0 1 1 0 1 0 1 1 1 0 1 0

Achillobator ? ? ? ? 0 ? ? ? ? ? ? ? ? ? ? 0 0 1 ? 1 0 0 1 1 1 1 1 3 1 ? ? 0 ? ? ? ? ? ? ? ? ? ? ? ? ? ? ? ? ? ? ? ? ? ? ? ? ? ? ? ? ? ?? ? ? ? ? ? ? ? ? ? ? ? ? ? ? ? ? ? ? ? ? ? ? ? ? ? ? ? ? ? ? ? ? ? ? ? ? ? ? ? ? ? ? ? ? ? ? ? ? ? ? ? ? ? ? ? ? ? ? ? ? ? ? ? ? ? ? ? ? ? ? ? ? ? ? ? ? 0 0 ? 0 0 0 ? ? 0 ? 1 1 0 1 ? ? ? ? ? 2 0 ? 0 ? ? 0 0 0 ? 0 0 0 ? 1 1 0 0 1 ? 1 0 1 ? ? ? ? ? ? ? ? ? ? ? ? 0 3 ? ? ? ? 1 ? ? ? ? ? ? ? ? ? ? ? ? ? 0 ? ? 0 1 ? 1 0 1 0 ? ? ? ? ? ? ? ? 1 1 ? ? ? ? ? ? ? ? ? ? ? ? ? ? ? ? ? ? ? ? ? ? ? 0 ? 0 ? ? ? ? ? ? ? ? ? ? ? 0 ? ? ? 0 0 0 ? 1 ? 0 ? 0 ? ? 0 2 1 0 1 1 1 0 1 1 0 1 0 ? 0 1 0 2 1 0 1 0 0 0 ? ? 0 0 0 0 0 0 0 1 0 0 0 0 0 ? 1 0 0 0 0 1 1 1 0 ? 1 1 1 ? 0 1 0 1 ? 0 0 0 ? ? ? 0 ? ? 0 0 0 ? 1 ? 0 0 ? ? ? ? ? ? 1 0 ? ? ? ? 0 ? ? ? ? ? ? ? 1 0 1 ? ? ? ? ? 1 ? ? ? 0

Dromaeosaurus ? ? 0 0 ? 0 0 0 0 0 ? 0 ? ? ? 0 0 1 ? ? 0 ? ? 1 1 ? ? ? ? ? ? 0 ? ? ? ? 0 0 ? ? ? ? ? 0 0 ? ? 0 1 2 0 1 0 1 0 0 1 ? 0 1 ? 00 1 1 1 1 1 1 1 1 0 ? ? 0 0 1 0 0 0 0 0 0 0 0 0 0 0 0 0 0 1 1 0 ? 1 0 0 ? 0 0 1 0 ? ? 0 0 0 ? 0 0 0 0 0 0 0 1 0 1 1 0 ? 0 0 0 0 1 1 1 1 0 0 0 0 0 2 ? 0 0 0 0 0 0 0 0 0 0 0 0 1 0 0 1 0 0 ? ? ? ? ? ? ? ? ? ? ? ? ? ? ? ? ? ? ? ? ? ? ? ? ? ? ? ? ? ? ? ? ? ? ? ? ? ? ? ? ? ? ? ? ? ? ? ? ? ? ? ? ? ? ? ? ? ? ? ? ? ? ? ? ? ? ? ? ? ? ? ? ? ? ? ? ? ? ? ? ? ? ? ? ? ? ? ? ? 0 ? ? 0 ? 0 ? ? ? ? ? ? ? ? ? ? ? ? ? ? ? ? ? ? ? ? ? ? ? ? ? ? ? ? ? ? ? ? ? ? ? ? ? ? ? ? ? ? ? ? ? ? ? ? ? ? ? ? ? ? ? ? ? ? ? ? ? ? ? ? ? ? ? ? ? ? ? ? ? ? ? ? ? ? ? ? ? ? ? ? ? ? ? ? ? ? ? ? ? ? ? ? ? ? ? ? ? ? ? ? ? ? ? ? ? ? ? ? ? ? 0 0 1 1 ? ? ? ? ? ? ? ? ? ? 1 1 0 1 0 1 ? 0 ? ? ? ? ? ? ? ?

Utahraptor ? ? ? ? ? 0 0 0 0 0 0 ? ? ? ? 0 ? 1 ? ? ? ? ? ? ? ? ? ? ? ? ? ? ? ? ? ? ? ? ? ? ? ? ? ? ? ? ? ? ? ? ? ? ? ? ? ? ? ? 0 ? ? ?? ? ? ? ? ? ? ? ? ? ? ? ? ? ? ? ? ? ? ? ? ? ? ? ? ? ? ? ? ? ? ? ? ? ? ? ? ? ? ? ? ? ? ? ? ? ? ? ? ? ? ? ? ? ? 0 1 1 0 0 ? 0 0 ? ? ? ? ? ? ? ? 0 0 2 1 0 0 ? 0 ? 0 0 ? ? ? ? ? 1 0 0 1 ? ? ? ? ? ? 0 ? ? ? ? ? ? 0 1 ? ? 0 ? ? 1 0 0 1 1 1 ? ? ? ? ? ? ? ? ? ? ? ? ? ? 0 3 ? ? ? ? ? 1 ? ? ? ? ? ? ? ? ? ? ? ? ? ? ? ? ? ? ? ? ? ? ? ? ? ? ? ? ? ? ? ? ? ? ? ? ? ? ? ? ? ? ? ? ? ? ? ? ? 0 ? ? ? ? ? 0 ? ? ? ? ? ? ? ? ? ? ? ? ? 0 ? ? ? ? ? ? ? ? ? ? ? ? ? ? ? ? ? ? ? ? ? ? ? ? ? ? ? ? ? ? ? ? ? ? ? ? ? ? ? ? ? ? ? ? ? ? ? ? ? ? ? ? ? ? ? ? ? ? ? ? ? ? ? ? ? ? ? ? ? ? ? ? ? ? 0 0 ? ? ? 0 1 1 0 ? ? ? ? ? ? ? ? ? ? ? ? ? ? ? ? ? ? ? ? ? ? ? ? ? ? ? ? ? ? ? ? 1 ? ? 1 1 0 1 0

Yurgovuchia ? ? ? ? ? ? ? ? ? ? ? ? ? ? ? ? ? ? ? ? ? ? ? ? ? ? ? ? ? ? ? ? ? ? ? ? ? ? ? ? ? ? ? ? ? ? ? ? ? ? ? ? ? ? ? ? ? ? ? ? ? ?? ? ? ? ? ? ? ? ? ? ? ? ? ? ? ? ? ? ? ? ? ? ? ? ? ? ? ? ? ? ? ? ? ? ? ? ? ? ? ? ? ? ? ? ? ? ? ? ? ? ? ? ? ? ? ? ? ? ? ? ? ? ? ? ? ? ? ? ? ? ? ? ? ? ? ? ? ? ? ? ? ? ? ? ? ? ? ? ? ? ? ? ? ? 1 ? ? 0 1 0 0 0 0 0 0 ? 0 0 0 1 ? 1 ? 0 1 1 1 0 ? ? ? ? ? ? ? ? 0 ? 2 ? 2 0 3 ? ? ? ? ? ? ? ? ? ? ? ? ? ? ? ? ? ? ? ? ? ? ? ? ? ? ? ? ? ? ? ? ? ? ? ? ? ? ? ? ? ? ? ? ? ? ? ? ? ? ? ? ? ? ? ? ? ? ? ? ? ? ? ? ? ? ? ? ? ? ? ? ? ? ? ? ? ? ? ? ? ? ? ? ? ? ? ? ? ? ? ? ? ? ? ? ? ? ? ? ? ? ? ? ? ? ? ? ? ? 1 0 ? ? 0 1 0 ? ? ? ? ? ? ? ? ? ? ? ? ? ? ? ? ? ? ? ? ? ? ? ? ? ? ? ? ? ? ? ? ? ? ? ? ? ? ? ? ? ? ? ? ? ? ? ? ? ? ? ? ? ? ? ? ? ? ? ? ? ? ? ? ? ? ? ? ? ? ? ? ? ? ? ? ? ? ? ? ? ?
