# Supplementary material for: Martharaptor greenriverensis, a New Theropod Dinosaur from the Lower Cretaceous of Utah
Source: PLoS One. 2012 Aug 29;7(8):e43911. doi: 10.1371/journal.pone.0043911 (PMC3430620; doi:10.1371/journal.pone.0043911)
Supplement: Appendix S3 — Synapomorphies of clades within Therizinosauroidea. (DOC) [file pone.0043911.s003.doc]

**Appendix S3: Synapomorphies of clades within Therizinosauroidea**

For each synapomorphy, the change of state is given in parentheses after the number by which the character is catalogued in the character list (Appendix I). For example, "32(0🡪1)" refers to a change from state 0 to state 1 for character 32. Bold face indicates synapomorphies recovered in both ACCRAN and DELTRAN lists. ACCTRAN “synapomorphies” of clades with only two terminal OTUs are artifacts and are not reported here.

Therizinosauroidea:

**21(0🡪1),** **116(0🡪1),** **144(2🡪0),** **154(0🡪1),** **166(0🡪1),** **228(0🡪1),** **230(0🡪1),** **286(0🡪1),** **358(1🡪2), 360(1🡪0)**

ACCTRAN only: 14(1🡪0), 17(1🡪2), 27(1🡪0), 57(0🡪1), 78(1🡪0), 83(0🡪1), 87(0🡪1), 94(1🡪0), 117(1🡪0), 148(0🡪1), 162(1🡪0), 212(0🡪3), 242(1🡪0), 288(0🡪1), 339(1🡪0), 340(0🡪1), 353(1🡪0), 356(1🡪0), 363(1🡪2), 389(0🡪1)

DELTRAN only: 107(0🡪2), 141(0🡪1), 349(0🡪1), 392(0🡪1)

*Beipiaosaurus* + (*Martharaptor* + (*Alxasaurus* + Therizinosauridae)):

**108(0🡪2),** **114(0🡪1),** **150(1🡪0),** **226(1🡪0),** **256(0🡪1),** **290(0🡪1),** **293(0🡪1), 300(0🡪1)**

ACCTRAN only: 81(0🡪2), 86(0🡪1), 151(1🡪0), 201(1🡪0), 237(1🡪0), 239(1🡪0), 273(0🡪1), 306(0🡪2), 308(0🡪1), 328(0🡪1)

DELTRAN only: 14(1🡪0), 17(1🡪2), 117(1🡪0), 212(0🡪3), 242(1🡪0), 356(1🡪0), 389(0🡪1)

*Martharaptor* + (*Alxasaurus* + Therizinosauridae):

**285(0🡪1)**

ACCTRAN only: 1(0🡪2), 134(0🡪1), 147(0🡪1), 149(0🡪2), 156(0🡪1), 235(0🡪1), 257(0🡪1), 263(1🡪0), 267(1🡪0), 301(0🡪1), 334(1🡪0)

*Alxasaurus* + Therizinosauridae:

**169(0🡪1),** **365(0🡪1)**

ACCTRAN only: 214(0🡪2), 279(0🡪1), 280(0🡪1), 364(0🡪1), 388(0🡪1)

DELTRAN only: 147(0🡪1), 148(0🡪1), 149(0🡪2), 201(1🡪0), 257(0🡪1), 301(0🡪1), 306(0🡪2), 328(0🡪1), 363(0🡪2)

Therizinosauridae:

**286(1🡪2), 292(1🡪2)**

ACCTRAN only: 244(0🡪1), 251(0🡪1), 372(0🡪1), 386(0🡪1)

*Suzhousaurus* + (*Nothronychus* + (*Segnosaurus* + (*Erliansaurus* + *Neimongosaurus* (*Erlikosaurus* + *Therizinosaurus*)))):

ACCTRAN only: 171(1🡪0), 181(0🡪1), 184(1🡪0)

DELTRAN only: 308(0🡪1), 324(0🡪1)

*Nothronychus* + (*Segnosaurus* + (*Erliansaurus* + *Neimongosaurus* (*Erlikosaurus* + *Therizinosaurus*)))

**182(0🡪1)**

ACCTRAN only: 185(0🡪2), 320(0🡪1)

DELTRAN only: 334(1🡪0), 364(0🡪1), 372(0🡪1), 386(0🡪1)

*Segnosaurus* + (*Erliansaurus* + *Neimongosaurus* (*Erlikosaurus* + *Therizinosaurus*))

**217(0🡪1)**

ACCTRAN only: 162(0🡪1), 265(0🡪1), 278(0🡪1)

DELTRAN only: 214: (0🡪2), 388(0🡪1)

*Erliansaurus* + *Neimongosaurus* (*Erlikosaurus* + *Therizinosaurus*)

**231(0🡪1)**

DELTRAN only: 235(0🡪1)

*Erlikosaurus* + *Therizinosaurus*

**229(0🡪1)**
